# Large-scale phosphoproteome analysis in seedling leaves of *Brachypodium distachyon* L.

**DOI:** 10.1186/1471-2164-15-375

**Published:** 2014-05-16

**Authors:** Dong-Wen Lv, Xin Li, Ming Zhang, Ai-Qin Gu, Shou-Min Zhen, Chang Wang, Xiao-Hui Li, Yue-Ming Yan

**Affiliations:** College of Life Science, Capital Normal University, Beijing, 100048 China

**Keywords:** Bd21, Leaf, Phosphoproteome, Transcription factors, Phosphorylation motif, Protein kinases

## Abstract

**Background:**

Protein phosphorylation is one of the most important post-translational modifications involved in the regulation of plant growth and development as well as diverse stress response. As a member of the *Poaceae*, *Brachypodium distachyon* L. is a new model plant for wheat and barley as well as several potential biofuel grasses such as switchgrass. Vegetative growth is vital for biomass accumulation of plants, but knowledge regarding the role of protein phosphorylation modification during vegetative growth, especially in biofuel plants, is far from comprehensive.

**Results:**

In this study, we carried out the first large-scale phosphoproteome analysis of seedling leaves in *Brachypodium* accession Bd21 using TiO_2_ microcolumns combined with liquid chromatography-tandem mass spectrometry (LC-MS/MS) and MaxQuant software. A total of 1470 phosphorylation sites in 950 phosphoproteins were identified, and these phosphoproteins were implicated in various molecular functions and basic cellular processes by gene ontology (GO) and Kyoto Encyclopedia of Genes and Genomes (KEGG) pathway analyses. Among the 950 phosphoproteins identified, 127 contained 3 to 8 phosphorylation sites. Conservation analysis showed that 93.4% of the 950 phosphoproteins had phosphorylation orthologs in other plant species. Motif-X analysis of the phosphorylation sites identified 13 significantly enriched phosphorylation motifs, of which 3 were novel phosphorylation motifs. Meanwhile, there were 91 phosphoproteins with both multiple phosphorylation sites and multiple phosphorylation motifs. In addition, we identified 58 phosphorylated transcription factors across 21 families and found out 6 significantly over-represented transcription factor families (C3H, Trihelix, CAMTA, TALE, MYB_related and CPP). Eighty-four protein kinases (PKs), 8 protein phosphatases (PPs) and 6 CESAs were recognized as phosphoproteins.

**Conclusions:**

Through a large-scale bioinformatics analysis of the phosphorylation data in seedling leaves, a complicated PKs- and PPs- centered network related to rapid vegetative growth was deciphered in *B. distachyon.* We revealed a MAPK cascade network that might play the crucial roles during the phosphorylation signal transduction in leaf growth and development. The phosphoproteins and phosphosites identified from our study expanded our knowledge of protein phosphorylation modification in plants, especially in monocots.

**Electronic supplementary material:**

The online version of this article (doi:10.1186/1471-2164-15-375) contains supplementary material, which is available to authorized users.

## Background

Protein phosphorylation is one of the common reversible post-translational modifications involved in the regulation of plant growth and diverse processes [1]. Various techniques have been developed for specific enrichment of phosphopeptides, among which Fe^3+^-IMAC (Immobilized metal affinity chromatography) [2, 3], TiO_2_-MOAC (Metal oxide affinity chromatography) [4, 5] and SCX (Strong cation exchange) chromatography [6, 7] are the three most common used methods for large-scale phosphoproteome studies. In recent years, through these phosphopeptide enrichment techniques combined with high-accuracy mass spectrometry (MS) and related bioinformatics, many large-scale phosphoproteomic analyses were performed in different plant species, such as *Arabidopsis thaliana* [8–11], *Oryza sativa* [10], *Medicago truncatula* [12, 13], *Glycine max* [11], *Zea mays* [14, 15] and *Triticum aestivum* [16, 17]. However, knowledge of protein phosphorylation modification in other plant species, especially for members of the *Poaceae*, is far from comprehensive.

Vegetative growth rapidly increases the photosynthetic capacity and size of plants, and is vital for biomass accumulation, especially in biofuel plants. *Brachypodium distachyon* L., a member of the *Pooideae* subfamily and a temperate wild annual grass endemic to the Mediterranean and Middle East [18], has rapidly become a model plant, especially for potential biofuel grasses such as switchgrass (*Panicum virgatum* L.). It possesses many attractive attributes such as a small diploid genome of 272 Mbp, short growth cycle, self-fertility and simple nutrient requirements [19] as well as competence to be efficiently transformed [20].

Leaf expansion is a major aspect of plant vegetative growth. It increases light capture, which powers photosynthesis and thus biomass production. Considerable works have investigated the mechanisms of leaf expansion [21–24], but little is known about post-translational phosphorylation modification of proteins during leaf expansion in the period of rapid vegetative growth. During this period the leaf cell wall undergoes dynamic changes to allow cells to expand, but at the same time cells must maintain the mechanical strength required to resist the forces of turgor pressure [25]. During plant growth and development, leaf cell numbers and size are rapidly increasing and cell walls must adapt to these changes. The components and mechanisms of underlying signaling systems to achieve this process remain largely unknown, but emerging evidences have implicated several receptor-like kinases as regulators of cell wall function [26–30].

Interactions of protein kinases (PKs) with their substrates are to a large extent determined by residues surrounding the phosphorylation sites and the pattern of residues is named as a phosphorylation motif [31, 32]. Motif-X analysis of large phosphorylation site data sets can detect significantly enriched phosphorylation motifs and predict the corresponding PKs [33]. Schwartz *et al.* [34] used Motif-X to determine phosphorylation motifs in yeast, fly, mouse, and man. Trost *et al.* [35] found eight specific phagosomal phosphorylation motifs induced by IFN-γ. Bennetzen *et al.* [36] used Motif-X to analyze phosphorylation sites during DNA damage responses and identified a novel [sxxQ] motif, confirming that Motif-X is a powerful program for revealing significant and novel phosphorylation motifs.

Recently, a large-scale proteomic and phosphoproteomic study of *B. distachyon* leaves under salt stress was performed in our laboratory [37]. In the present study, we focused on phosphorylation modification during seedling leaf growth and development at the omics level for the first time in *B. distachyon* and revealed a complicated phosphorylation signal transduction network during rapid leaf growth and development.

## Results and discussion

### Identification of phosphorylation sites and phosphoproteins

**Figure 1:**
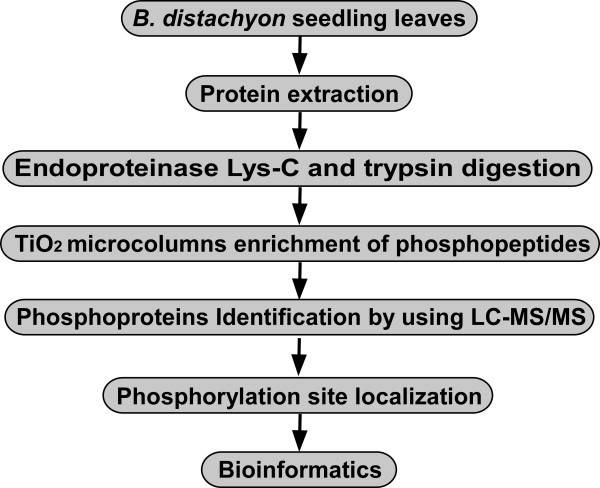
**Strategy for a large-scale phosphoproteomics study on seedling leaves of** ***B. distachyon*** **.**

**Figure 2:**
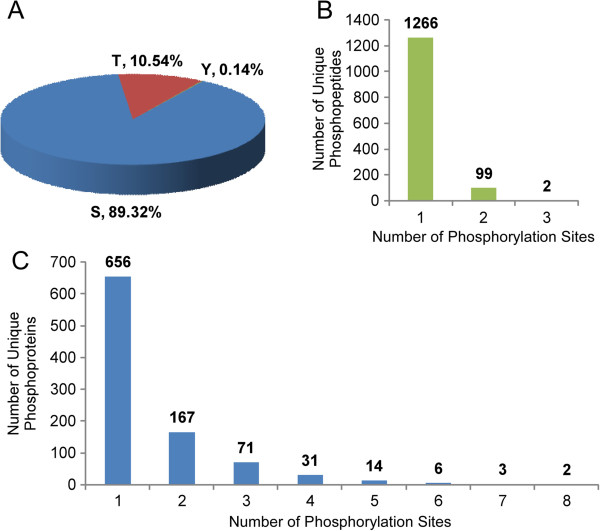
**Analysis of identified phosphorylated sites. (A)** Distribution of the phosphorylated amino acids. **(B)** Distribution of phosphorylation sites in the phosphopeptides. **(C)** Distribution of phosphorylation sites in the phosphoproteins.

In this study, a large-scale phosphoproteome analysis in *B. distachyon* seedling leaves was performed to explore the complex protein phosphorylation network of signaling and regulatory events. The strategy used in this study is shown in Figure 1. To increase the number of identified phosphopeptides and eliminate false positives, three biological replicates were used for phosphopeptide enrichment and LC-MS/MS analysis. A total of 1937 phosphopeptides containing 2449 phosphorylation sites were identified (Additional file 1: Table S1). The raw mass spectrometry proteomics data have been deposited to the ProteomeXchange Consortium (http://proteomecentral.proteomexchange.org) via the PRIDE partner repository [38] with the dataset identifier PXD000868. Only the phosphopeptides with unambiguous (class I) phosphorylation sites identified from three biological repeats were used for further analysis. Finally, the 1470 unambiguous phosphorylation sites distributed in 1367 phosphopeptides, corresponding to 950 phosphoproteins, were screened and used for further analysis (Additional file 1: Table S1, sheet A). Among the 1470 unambiguous phosphorylation sites, 1313 Ser, 155 Thr, and 2 Tyr phosphorylation sites were identified, accounting for 89.32%, 10.54%, and 0.14% of the total sites, respectively (Figure 2, panel A). For the lower identification of phosphorylated tyrosine, there are three possible reasons: first, we didn’t perform focused analysis during MS runs in this study, which may lead to the reduced discovery of the low abundant Tyr phosphopeptides; second, the recovery rate of phospho-Tyr peptides could be different because of the enrichment techniques and data processing softwares; third, different organs, development stages or stress treatments can also lead to the variation of the phospho-Tyr identification. The review by Ghelis [39] also gave similar explanations for the lower phospho-Tyr proportion in plants. The distributions of phosphorylation sites in phosphopeptides and phosphoproteins were shown in Figure 2, panel B and C, respectively. Among the 1367 phosphopeptides, 1266 (92.61%) of them each contained one phosphorylation sites, only 99 (7.24%) contained two and 2 (0.15%) contained three phosphorylation sites (Figure 2, panel B). From the phosphoprotein perspective, 656 (69.05%) and 167 (17.58%) of the 950 phosphoproteins were detected to have 1 and 2 phosphorylation sites, respectively. One hundred and twenty seven phosphoproteins contained more than two phosphorylation sites, of which 25 had at least five phosphorylation sites. Two phosphoproteins (Bradi1g66870.1 and Bradi3g53907.1) each possessed 8 phosphorylation sites (Figure 2, panel C). The representative mass spectra of all the seven phosphopeptides of Bradi1g66870.1 containing the 8 phosphorylated sites were shown in Additional file 2: Figure S1.

**Figure 3:**
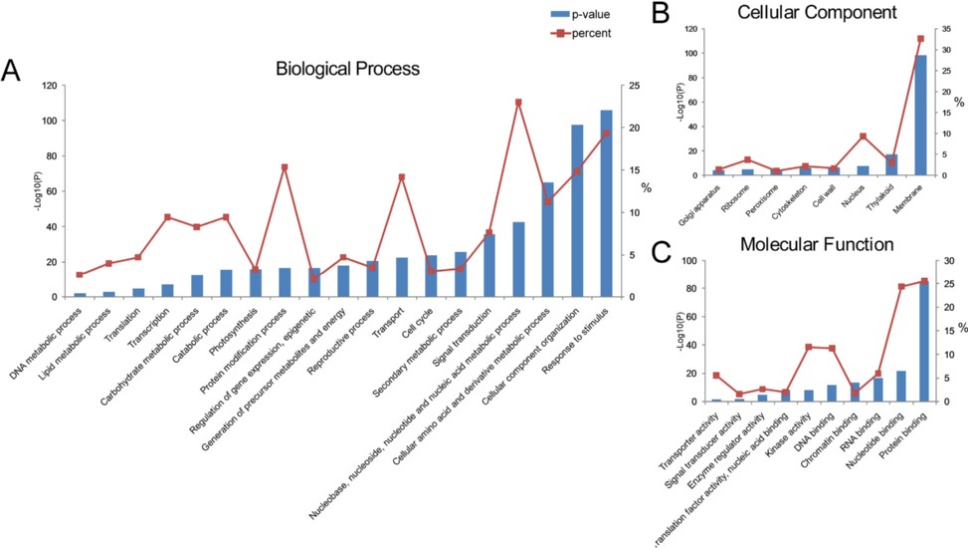
**Gene ontology enrichment analysis of the 950 identified phosphoproteins. (A)** Biological processes. **(B)** Cellular components. **(C)** Molecular functions.

In order to obtain an overview of phosphorylation events in seedling leaves of *B. distachyon*, all of the 950 identified phosphoproteins were used to perform GO, Pfam annotation. Finally, 840 of them were annotated by Blast2GO and 804 of them possessed Pfam domains (Additional file 1: Table S1, sheet B). Significantly (FDR adjusted p < 0.05) enriched GO items of biological process, molecular function and cellular component are shown in Figure 3. Both the percent and p-value of each item were displayed to evaluate these GO items. From the biological process perspective, “nucleobase, nucleoside, nucleotide and nucleic acid metabolic process (23.05%, GO:0006139, FDR:2.6E - 43)”, “response to stimulus (19.37%, GO:0050896, FDR:1.5E - 106)”, “Protein modification process (15.36%, GO:0006464, FDR:3.8E - 17)”, “cellular component organization (14.84%, GO:0016043, FDR:2.3E - 98)”, “transport (14.21%, GO:0006810, FDR:4.5E - 23)”, and “cellular amino acid and derivative metabolic process (11.26%, GO:0006519, FDR:9.9E - 66)” were over-represented (Figure 3, panel A). From the cellular component perspective, “membrane (32.63%, GO:0016020, FDR:8.7E - 99)” and “nucleus (9.37%, GO:0005634, FDR:1.5E - 8)” were significantly enriched (Figure 3, panel B). From the molecular function perspective, “protein binding (25.57%, GO:0005515, FDR:6.1E - 86)”, “nucleotide binding (24.42%, GO:0000166, FDR:3.1E - 22)”, “kinase activity (11.58%, GO:0016301, FDR:1.3E - 8)” and “DNA binding (11.37%, GO:0003677, FDR:3E - 12)” were the highly enriched molecular function items (Figure 3, panel C).

### Conservation and KEGG pathway analyses of phosphoproteins

To investigate the conservation of phosphoproteins between *B. distachyon* and other plant species, the sequences of the 950 *B. distachyon* phosphoproteins were used as queries to blast a phosphoprotein database which were constructed based on datasets from P3DB (Plant Protein Phosphorylation DataBase) [40], MORE (Medicago-Omics Repository) [13] and PhosPhAt 4.0 [41]. *Oryza sativa* and *Arabidopsis thaliana*, as the model species of monocots and dicots, respectively, possess more comprehensive phosphorylaton information than other species. Recently, a large phosphoprotein dataset of the model legume *Medicago truncatula* was released [13]. *B. distachyon* was therefore compared with the three species to determine the degree of conservation of the phosphoproteins among them. The threshold was set as Score ≥ 80, E–value < 1E–10 and Identity ≥ 30%. Finally, 582 (61.3%) phosphoproteins were identified with phosphorylation orthologs in all the three species, 202 (21.3%) had phosphorylation orthologs in two of the three species, and 103 (10.8%) had phosphorylation orthologs in only one species (Additional file 3: Table S2). Only 63 phosphoproteins had no phosphorylation orthologs in the three species. The conserved analysis results are shown in Additional file 3: Table S2. Further biological process enrichment analysis of the 582 highly conserved phosphoproteins (Additional file 4: Figure S2, panel A) showed that “cellular protein modification process (GO:0006464, FDR:2.4E–2)” and “signal transduction (GO:0007165, FDR:5.0E–3)” were significantly over-represented from the total identified phosphoproteins (Additional file 4: Figure S2, panel B).

To reveal the phosphoprotein-associated pathways, the 950 phosphoproteins were mapped to KEGG; 31 *B. distachyon* KEGG pathways with not less than 3 hits were highlighted by the phosphoproteins identified in this study (Additional file 5: Table S3 and Additional file 6: Figure S3). Many fundamental biological pathways were highlighted by the mapping of phosphoproteins from this work, including carbohydrate metabolism involving carbon metabolism (Additional file 6: Figure S3, panel D), glycolysis/gluconeogenesis (panel G), pyruvate metabolism (panel N) and pentose phosphate pathway (panel P), energy metabolism including carbon fixation (panel I), starch and sucrose metabolism (panel L) and oxidative phosphorylation (panel R), and nucleotide metabolism including purine metabolism (panel M). For genetic information processing, transcription in spliceosomes (panel A), translation related to RNA transport (panel C), ribosome (panel E) and mRNA surveillance (panel F) were highly enriched. Two signal transduction pathways were particularly evident: the plant hormone signal transduction pathway (panel Q) and the phosphatidylinositol signaling system (panel AC). Further analysis of the 582 highly conserved phosphoproteins-related pathways showed that carbohydrate metabolism and energy metabolism-associated pathways, splicing, RNA transport and mRNA surveillance pathways stood out (Additional file 5: Table S3). The result of KEGG pathway analysis was consistent with that from GO analysis, indicating that the methods used here were effective and reliable.

### Phosphorylation of transcription factors

**Figure 4:**
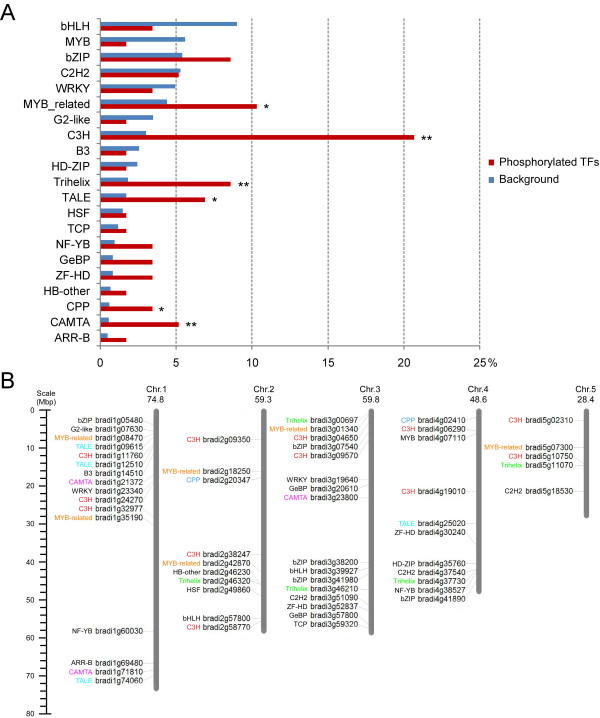
**Analysis of phosphorylated transcription factors (TFs). (A)** TF family enrichment analysis by using a two-sided test of the hypergeometric distribution. The number of phosphorylated TFs belonging to a TF family was compared against the total number of TFs in that family of *B. distachyon*. The six significantly over-represented TF families are marked with *p < 0.05 or **p < 0.01. **(B)** Chromosomal localization of genes of all phosphorylated TFs on the five chromosomes of *B. distachyon*. The chromosome numbers and their size (Mb) are indicated at the top of each bar.

In growth and development of plants, many transcription factors (TFs) are affected through phosphorylation by PKs or dephosphorylation by protein phosphatases (PPs), which in turn either positively or negatively regulate TF activity in facilitating a program of gene expression that results in changed cell behavior [42]. Based on GO and KEGG pathway analyses, transcription-associated proteins were one of the most significant groups of all phosphoproteins, and TFs particularly were the main constituents. Fifty eight TFs belonging to 21 different families were identified (Table 1) based on alignments of our phosphoproteins with *B. distachyon* TFs in PlantTFDB (http://planttfdb.cbi.pku.edu.cn/index.php?sp=Bdi) [43]. In PlantTFDB, there are 1751 *B. distachyon* TFs classified into 56 families. To determine the TF families significantly regulated by phosphorylation, a TF family enrichment test was performed from 21 TF families contained 58 TFs compared with the background set (1751 TFs from 56 TF families in *B. distachyon*). The number of phosphorylated TFs belonging to a TF family was compared against the total number of TFs in that family of *B. distachyon*. Finally, 6 significantly over-represented (p < 0.05) TF families (C3H, Trihelix, CAMTA, TALE, MYB_related, and CPP) were found (Figure 4, panel A). Bd21 is diploid inbred line with its genome size about 272 Mb and contains 26,552 gene loci, coding for 31,029 distinct mRNA molecules [18]. Genes of all the 58 TFs were localized in the five *Brachypodium* chromosomes (Figure 4, panel B). The distribution of the six significantly over-represented TF families had no obvious chromosomal preference.

**Table 1:** **Phosphorylated transcription factors identified in this study**

Phosphoprotein no.	Number of phosphorylation sites	Phosphorylation sites	TF family
Bradi1g69480.1	1	S372	ARR-B
Bradi1g14510.1	1	S576	B3
Bradi2g57800.1	1	S161	bHLH
Bradi3g39927.1	4	S11;S30;S185;S258	bHLH
Bradi1g05480.1	3	S6;S71;S399	bZIP
Bradi3g07540.1	1	S32	bZIP
Bradi3g38200.1	1	S112	bZIP
Bradi3g41980.1	5	S38;S114;S241;S257;S271	bZIP
Bradi4g41890.1	1	S29	bZIP
Bradi3g51090.1	1	S73	C2H2
Bradi4g37540.1	1	S56	C2H2
Bradi5g18530.1	1	S81	C2H2
Bradi1g11760.1	4	S208;S511;S524; S641	C3H
Bradi1g24270.1	2	S493;S563	C3H
Bradi1g32977.1	1	S353	C3H
Bradi2g09350.1	1	S435	C3H
Bradi2g38247.1	2	S397;S550	C3H
Bradi2g58770.1	2	S206;S286	C3H
Bradi3g04650.1	1	S258	C3H
Bradi3g09570.1	1	S38	C3H
Bradi4g06290.1	2	S437;S448	C3H
Bradi4g19010.1	2	S215;S219	C3H
Bradi5g02310.1	1	T35	C3H
Bradi5g10750.1	5	S19;S21;S38;S42;S46	C3H
Bradi1g21372.1	3	S329;S777;S929	CAMTA
Bradi1g71810.2	2	S594;S973	CAMTA
Bradi3g23800.1	1	S956	CAMTA
Bradi2g20347.1	1	S234	CPP
Bradi4g02410.1	1	S226	CPP
Bradi1g07630.1	1	S190	G2-like
Bradi3g20610.1	2	S100;S358	GeBP
Bradi3g57800.1	1	T86	GeBP
Bradi2g46230.1	1	S952	HB-other
Bradi4g35760.1	1	S863	HD-ZIP
Bradi2g49860.1	1	S230	HSF
Bradi4g07110.1	1	S416	MYB
Bradi1g08470.1	1	S204	MYB_related
Bradi1g35190.1	1	S265	MYB_related
Bradi2g18250.1	1	S426	MYB_related
Bradi2g42870.1	1	S105	MYB_related
Bradi3g01340.3	1	S1081	MYB_related
Bradi5g07300.1	1	S72	MYB_related
Bradi1g60030.1	3	S4;S8;T124	NF-YB
Bradi4g38527.1	1	S19	NF-YB
Bradi1g09615.1	1	S421	TALE
Bradi1g12510.1	1	S7	TALE
Bradi1g74060.1	1	S448	TALE
Bradi4g25020.1	1	S75	TALE
Bradi3g59320.1	1	S156	TCP
Bradi2g46320.1	2	S20;S143	Trihelix
Bradi3g00697.1	3	S282;S418;S665	Trihelix
Bradi3g46210.1	1	S65	Trihelix
Bradi4g37730.1	1	S224	Trihelix
Bradi5g11070.1	1	S78	Trihelix
Bradi1g23340.1	1	S105	WRKY
Bradi3g19640.1	4	S332;T352;T354;S409	WRKY
Bradi3g52837.1	1	S265	ZF-HD
Bradi4g30240.1	1	S96	ZF-HD

### Phosphorylation motif enrichment analysis

**Figure 5:**
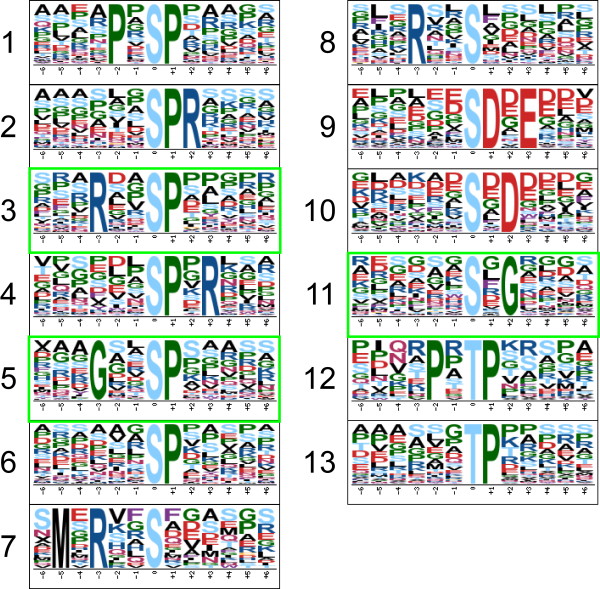
**Significantly enriched phosphorylation motifs.** Among these motifs, 10 are known phosphorylation motifs, and 3 are newly identified (enclosed into green boxes). Detailed information and putatively associated kinases are shown in Additional file 7: Table S4.

To date, about 14,000 and 12,000 phosphorylation sites representing nearly 4100 and 4900 phosphoproteins in P3DB have been found in *A. thaliana* and *O. sativa*, respectively [40]. For *A. thaliana*, PhosPhAt 4.0 contains 12,613 experimental phosphorylation sites and 5663 phosphoproteins [41]. In addition, a large dataset for the model legume *M. truncatula* containing 13,506 phosphorylation sites localized in 3926 phosphoproteins was available in MORE [13]. Phosphorylation modification of these sites are regulated by PKs, which account for about 5.5% of the *Arabidopsis* genome [44]. Different PKs have preference for specific substrates, and many kinase-associated phosphorylation motifs have been deciphered [45–49]. The Motif-X online tool was used in this study to identify the phosphorylation motifs, and 13 different motifs were enriched, including 11 Ser and 2 Thr motifs (Figure 5 and Additional file 7: Table S4, sheet A). The phosphoproteins containing each of the 13 motifs (listed in Additional file 7: Table S4, sheet B) were further used in GO enrichment analysis. As shown in Additional file 8: Figure S4, DNA binding or RNA binding was enriched from phosphoproteins with the motifs 1, 2, 3, 6, 10 or 13, whereas translation factor activity was enriched from phosphoproteins with the motifs 9 or 10. Phosphoproteins containing motifs 1, 4 or 9 were mainly involved in the cell cycle and located in ribosomes. Lipid binding and receptor activity were over-represented from motifs 2 and 8, respectively, and tropism was enriched from phosphoproteins containing the motif 12. Organic cyclic compound binding and heterocyclic compound binding functions were enriched from the novel motif 5. No GO terms were significantly enriched from phosphoproteins containing motifs 7 or 11.

**Figure 6:**
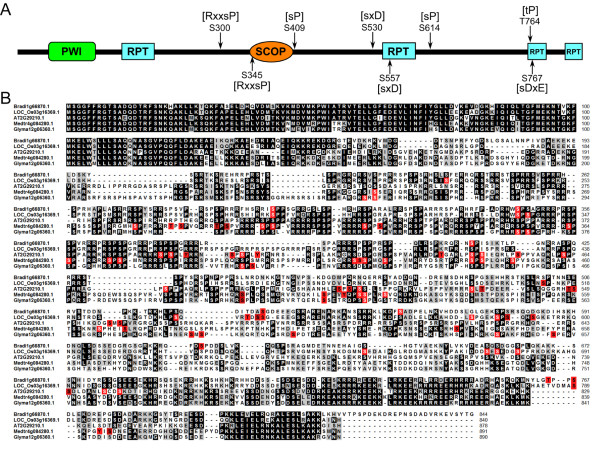
**Phosphorylation sites analysis of Bradi1g66870.1. (A)** Domain structures of Bradi1g66870.1 and distribution of the 8 phosphorylated sites and related phosphorylation motifs on the sequence of Bradi1g66870.1. **(B)** Sequence alignments among Bradi1g66870.1 and its phosphorylation orthologs from *O. sativa*, *A. thaliana*, *M. truncatula*, and *G. max*. Phosphorylation sites identified in this study and based on the information from P3DB are highlighted in red.

According to the literature and databases [45–49], 10 of these motifs were specific to known PKs and 3 had no corresponding known kinases (Additional file 7: Table S4, sheet A). Ten known motifs included proline-directed motifs such as [s/tP] (Motifs 6 and 13) and [Pxs/tP] (Motifs 1 and 12), which were potential substrates of mitogen-activated PK (MAPK), cyclin-dependent kinase (CDK) and CDK-like kinase. Basic motifs [sPR] (Motif 2) and [sPxR] (Motif 4) were recognized by growth-associated histone kinase (GHK), CDK or cell division cycle 2 (CDC2) kinase. [Rxxs] (Motif 8) was recognized by calcium/calmodulin-dependent PK II (CaMK-II). [MxRxxs] (Motif 7) was recognized by C-terminal Src Kinase-homologous Kinase 1 (CHK1). Acidic motifs [sDxE] (Motif 9) and [sxD] (Motif 10) were recognized by casein kinase-II (CK-II). The other 3 phosphorylation motifs had no known specific kinases and were therefore regarded as novel phosphorylation motifs found in *B. distachyon*. Further analysis of these motifs showed that [RxxsP] (Motif 3), as a basic motif, is a combination of [sP] (Motif 6) and [Rxxs] (Motif 8); and this motif may be recognized by MAPK, CDK or CaMK. [GxxsP] (Motif 5) is similar to [GsP], which is recognized by glycogen synthase kinase 3 (GSK-3), MAPK or CDK5. [sxG] (Motif 11) may be recognized by CKII [50]. Further inspection of the 127 phosphoproteins containing multiple phosphorylation sites (Additional file 1: Table S1, sheet B) showed that 122 of them possessed at least one of the 13 phosphorylation motifs (Additional file 7: Table S4, sheet C). Among them, 91 phosphoproteins possessed two or more kinds of motifs. Phosphorylation sites of most phosphoproteins were motif-specific, but some other phosphoproteins each contained multiple phosphorylation sites belonging the same motif. Two phosphoproteins (Bradi1g51560.1 and Bradi3g03690.1) each contained four phosphorylation sites specific to one motif (Additional file 7: Table S4, sheet C). This suggests that most phosphoproteins with multiple sites are regulated by multiple PKs, and a small part of them are activated by only one kind of PKs. The phosphoprotein Bradi1g66870.1 contained eight phosphorylation sites belonging to five kinds of motifs (Figure 6, panel A), which may be regulated by different PKs such as MAPK, CDK and CK-II. Conservation analysis showed that phosphorylation orthologs in other species were also identified as phosphoproteins with many phosphosites, and 5 of the 8 identified phosphorylation sites in Bradi1g66870.1 were also identified in other species whereas the other three were the newly identified phosphorylation sites present only in *B. distachyon* (Figure 6, panel B).

### Phosphorylation of PKs and PPs

There are 989 PKs and 131 PPs in *Arabidopsis* according to PlantsP (http://plantsp.genomics.purdue.edu/) [51]. In this study, 84 PKs and 9 PPs were identified as phosphoproteins (Table 2). To identify the interactions between the phosphorylated PKs and PPs and their potential substrates, protein-protein interaction (PPI) analysis was conducted by using the Search Tool for Retrieval of Interacting Genes/Proteins (STRING). All 478 euKaryotic Orthologous Groups (KOGs) (Additional file 1: Table S1, sheet B) representing 685 of the 950 phosphoproteins were used to construct the phosphoprotein interaction network. To improve the accuracy of PPI analysis, the confidence level (score) was set to a high value (0.900). Finally, a complicated PPI network centered by PKs and PPs was constructed and displayed by using Cytoscape software (Additional file 9: Figure S5). Depending on the homology comparison with the PKs and PPs of *Arabidopsis* in the PlantsP database, the phosphorylated PKs and PPs identified in our study were classified into six classes (Table 1): transmembrane receptor kinases and related non-transmembrane kinases, ATN1/CTR1/EDR1/GmPK6-like kinases, casein kinase I, non-transmembrane PKs, other kinases, and plant phosphatases.

**Table 2:** **Phosphorylated PKs and PPs identified in this study**

Phosphoprotein no.	Phosphorylation site count	Phosphorylation sites	KOG no.
**Class 1-transmembrane receptor kinases and related non-transmembrane kinases**			
Bradi1g07010.1	1	S372	KOG1187
Bradi1g20750.1	1	S735	KOG1187
Bradi1g35477.1	1	S697	KOG1187
Bradi1g58260.1	3	S741;S743;S733	KOG1187
Bradi1g59210.1	1	S837	KOG1187
Bradi1g60397.2	2	S345;S370	KOG1187
Bradi1g72430.1	2	S382;S389	KOG1187
Bradi1g76660.1	1	S770	KOG1187
Bradi2g01180.1	1	S133	KOG1187
Bradi2g12120.1	1	S149	KOG1187
Bradi2g27350.1	1	S444	KOG1187
Bradi2g34630.1	1	T263	KOG1187
Bradi2g43110.1	2	S654;T674	KOG1187
Bradi2g47000.1	1	S682	KOG1187
Bradi3g08660.1	1	S1031	KOG1187
Bradi3g32330.1	1	S233	KOG1187
Bradi3g40370.1	1	S93	KOG1187
Bradi3g44250.1	1	S508	KOG1187
Bradi3g49160.1	1	S693	KOG1187
Bradi3g53350.1	2	S546;S535	KOG1187
Bradi4g35050.1	1	S24	KOG1187
Bradi5g03410.1	3	S342;T666;T672	KOG1187
Bradi5g12540.1	2	S665;S325	KOG1187
Bradi5g13960.1	1	S612	KOG1187
**Class 2-ATN1/CTR1/EDR1/GmPK6 like kinases**			
Bradi1g00580.1	2	S514;S503	KOG0192
Bradi1g28110.2	1	S12	KOG0192
Bradi1g28950.2	3	S1105;S761;S569	KOG0192
Bradi1g30720.1	1	S477	KOG0192
Bradi1g47570.1	1	T14	KOG0192
Bradi2g46340.1	2	S333;S410	KOG0192
Bradi2g49790.1	1	S129	KOG0192
Bradi3g09170.1	3	S478;S481;S400	KOG0192
Bradi3g60210.1	1	S811	KOG0192
Bradi4g36880.1	2	S27;S153	KOG0192
Bradi4g41870.1	4	S23;S37;S234;T417	KOG0192
**Class 3-casein kinase I**			
Bradi2g14310.1	1	S415	KOG1163
Bradi2g47850.1	1	S417	KOG1163
Bradi3g54860.1	1	S456	KOG1163
Bradi2g33037.2	1	S10	KOG1164
**Class 4-non-transmembrane PKs**			
Bradi1g06300.1	1	S137	KOG0032
Bradi1g24240.1	1	S524	KOG0032
Bradi1g35090.1	4	S102;S26;T36;T46	KOG0032
Bradi1g56970.1	2	S557;S85	KOG0032
Bradi2g15520.1	1	S537	KOG0032
Bradi4g24390.1	1	S504	KOG0032
Bradi4g39870.1	1	S538	KOG0032
Bradi4g40300.1	1	S509	KOG0032
Bradi5g19430.1	4	S40;S549;S553;S16	KOG0032
Bradi1g10970.1	1	S230	KOG0198
Bradi2g26547.1	1	S972	KOG0198
Bradi4g22760.1	1	S155	KOG0198
Bradi5g10670.1	1	S607	KOG0198
Bradi5g18180.1	2	S335;S337	KOG0198
Bradi5g24870.1	1	S893	KOG0198
Bradi1g26670.1	1	S322	KOG0201
Bradi1g42257.2	3	S502;S336;S477	KOG0581
Bradi1g51000.1	1	T25	KOG0581
Bradi1g75150.1	1	T30	KOG0581
Bradi1g77700.1	3	S538;S562;S625	KOG0582
Bradi3g05890.1	2	S330;T331	KOG0582
Bradi3g31110.1	1	S537	KOG0582
Bradi1g07620.1	2	S179;S183	KOG0583
Bradi2g56267.1	1	S158	KOG0583
Bradi1g23970.1	1	S614	KOG0584
Bradi2g56460.1	1	S394	KOG0592
Bradi1g18450.2	1	S375	KOG0600
Bradi1g62440.1	1	S77	KOG0600
Bradi2g12937.2	1	S509	KOG0600
Bradi2g26680.1	1	T198	KOG0600
Bradi2g61310.1	2	S93;T198	KOG0600
Bradi1g11380.1	3	S635;S654;T28	KOG0606
Bradi4g45310.1	4	S378;S385;S286;S297	KOG0610
Bradi5g07360.1	3	S508;S306;T304	KOG0610
Bradi3g10350.1	2	S466;S427	KOG0614
Bradi2g38590.1	1	Y235	KOG0658
Bradi2g26510.1	2	S337;T173	KOG0659
Bradi1g49100.1	2	T218;Y220	KOG0660
Bradi5g13980.1	3	S125;S190;S693	KOG0663
Bradi1g10530.1	1	S546	KOG0667
Bradi4g00230.1	1	T544	KOG0670
Bradi2g42320.1	1	S33	KOG0671
**Class 5-other kinases**			
Bradi1g08540.1	1	S92	KOG1151
Bradi1g26580.1	1	S30	KOG1166
Bradi2g17660.1	3	S545;T531;T540	KOG0594
**Class 6-plant phosphatases**			
Bradi2g36370.1	2	S847;S500	KOG0374
Bradi1g24400.1	1	S414	KOG0698
Bradi1g47710.1	1	S339	KOG0698
Bradi3g39540.1	1	S504	KOG0698
Bradi3g52110.1	2	S121;S131	KOG0700
Bradi1g42810.1	1	S232	KOG1716
Bradi2g37450.1	3	S283;S465;T92	KOG1716
Bradi4g08080.1	2	S73;S113	KOG2283

**Figure 7:**
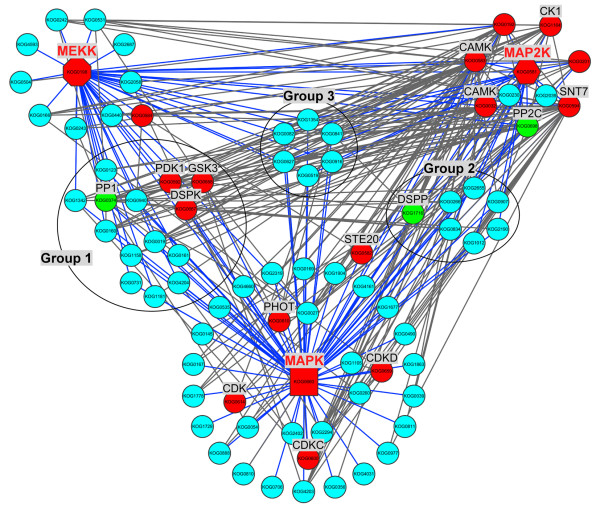
**Phosphorylation regulatory network centered by MAPK cascade.** Protein kinases and protein phosphatases are highlighted in red and green, respectively and other phosphoproteins identified in this study are shown with sky-blue nodes. Edges with blue color represent the phosphoproteins with direct relationship to MEKK, MAP2K or MAPK.

MAPK cascades are universal signal transduction modules in plants and are involved in responses to various biotic and abiotic stresses, hormones, cell division and developmental processes [52]. Activation of a MAPK cascade can lead to changes in gene expression relating to cell wall biogenesis [53, 54]. MAPK cascades contained three PK families, the MAPK, MAPK kinase (MAP2K) and MAPK kinase kinase (MEKK) families. In this study, one MAPK (Bradi1g49100.1; KOG0660), one MAP2K (Bradi1g51000.1; KOG0581) and five MEKK (Bradi1g10970.1, Bradi4g22760.1, Bradi5g10670.1, Bradi5g18180.1, and Bradi5g24870.1; KOG0198) were identified as phosphoproteins, and conserved among *B. distachyon, O. sativa*, *A. thaliana* and *M. truncatula* (Additional file 3: Table S2), clearly indicating the fundamental roles of MAPK cascades in plants. A sub-network (Figure 7) centered by MEKK, MAP2K and MAPK was extracted from the whole PPI network mentioned above. From the sub-network, the MAPK family was the crucial PK in MAPK cascades and directly activated the substrates, so most phosphoproteins including many other kind of PKs (CDK, PHOT and STE20) interacted with MAPK. MAPK and many other PKs such as CK1 and SNT7 and PP2C were activated by MAP2K whereas MAP2K was activated by MEKK. Many phosphoproteins interacted simultaneously with MEKK and MAPK (Group 1) or MAP2K and MAPK (Group 2), and some interacted with MEKK, MAP2K and MAPK (Group 3). Several PKs and PPs, including GSK3, PDK1, DSPK and PP1, were also regulated by both MEKK and MAPK whereas only DSPP was regulated by both MAP2K and MAPK. Two phosphorylation sites (Table 2 and Additional file 1: Table S1, sheet A) were identified from BdMAPK (Bradi1g49100.1) and one of them was phosphorylated at Tyr220. An *Arabidopsis* MAPK (AtMPK4) is phosphorylated at a Tyr residue during the activation process [55]. A previous study revealed that plant MAPKs contained a conserved phosphorylation motif, [tD/Ey] [56]. Interestingly, the BdMAPK we identified also contains this motif. Besides, we identified phosphorylation of 11 BdCDPKs and 4 BdCDKs, which may function in defense response and regulation of cell division and differentiation [57–59].

### Phosphorylation of signal transduction proteins

In plants, many signal pathways are involved in vegetative growth. In the present study, KEGG pathway analysis showed that the plant hormone signal transduction pathway (Additional file 6: Figure S3, panel Q) and phosphatidylinositol signaling system (Additional file 6: Figure S3, panel AC) played important roles through phosphorylation modification of some crucial proteins. The hormones include cytokinin, abscisic acid, ethylene and brassinosteroid which mainly regulate cell division, cell elongation and shoot initiation. A homolog of type-B *Arabidopsis* response regulator (B-ARR, Bradi1g69480.1) was found in the cytokinin regulated pathway. B-ARR is a DNA-binding transcriptional regulator, whose activity is regulated by phosphorylation of its phospho-accepting receiver domain through the His-kinase-mediated His to Asp phosphorelay and lack of this domain disturbs the development of young seedlings [60]. Likewise, other conserved phosphoproteins including a bZIP TF (Bradi3g38200.1), two ethylene-insensitive protein (Bradi4g08380.1 and Bradi5g00700.1) and three serine/threonine PKs (Bradi1g07620.1, Bradi3g32330.1 and Bradi2g56267.1) were involved in the abscisic acid, ethylene or brassinosteroid regulated signal transduction pathways. Another signal transduction pathway shown by KEGG was the phosphatidylinositol signaling system (Additional file 6: Figure S3, panel AC) that is associated with cell proliferation and differentiation [61]. Phosphatidylinositol-3,4,5-trisphosphate 3-phosphatase (PTEN, Bradi4g08080.1) acts as a lipid phosphatase to produce phosphatidylinositol 4,5-biphosphate (PtdIns(4,5)P2). Phosphatidylinositol-4- phosphate 5-kinase (Bradi1g44047.1) is involved in PtdIns(4,5)P2 synthesis. Phosphoinositide phospholipase C (PLC, Bradi1g16560.1) mediates the production of second messenger molecules diacylglycerol (DAG) and inositol 1,4,5-trisphosphate (IP3) by hydrolysis of PtdIns(4,5)P2. The three enzymes were all identified as phosphoproteins (Additional file 1: Table S1).

### Phosphorylation of proteins related to cell wall expansion

The *Poaceae* family can be distinguished from most other land plants by the composition of their cell walls [62, 63]. *B. distachyon* is a good model for cell wall (mostly primary wall) studies of temperate grasses [24, 64]. Cell wall loosening is essential for leaf expansion during vegetative growth of plants, and this must be balanced with polymer synthesis and wall restrengthening to prevent the cell wall from rupture. Multiple receptor-like PKs (RLKs) are implicated in cell wall signaling [30]. The RLK (Bradi2g12120.1), a conserved phosphoprotein among *B. distachyon*, *O. sativa* and *A. thaliana*, was phosphorylated at Ser149 (Table 2). Hématy *et al.* [65] found a RLK (THE1) mediating the response of growing plant cells to inhibition of cellulose synthesis in *Arabidopsis* and that it can be phosphorylated at a Thr residue. The phosphorylation of RLK identified in our study may be involved in cell wall remodeling during leaf development. A wall-associated receptor kinase (WAK, Bradi3g49160.1), as a kind of RLK, has the potential to serve as both linkers of the cell wall to the plasma membrane and as a signaling molecule. WAKs also function in pathogen response and cell expansion and elongation [27, 66, 67]. WAK proteins contain a cytoplasmic Ser/Thr kinase (STK) domain, a transmembrane domain, and an extra cytoplasmic region with several epidermal growth factor (EGF) repeats [28, 68]. Interestingly, the phosphorylated site of this BdWAK was located on the C-terminal (Ser693) in our study, which was outside these domains above. As a kind of RLK, phosphorylation of this BdWAK may regulate functions of itself and further mediate the phosphorylation of enzymes involved in synthesis of cellulose in cell walls.

The primary cell walls of grasses consist mainly of cellulose, arabinoxylan and uniquely (1, 3; 1, 4)-β-D-glucan, small quantities of structural proteins, and low levels of pectin and xyloglucan [63, 69–71]. Cellulose is by far the most abundant biological polymer on earth, and is synthesized by cellulose synthase (CESA) contained in plasma membrane–localized complexes. In *A. thaliana*, CESA1, CESA3 and CESA6 interact with each other and are largely responsible for cellulose production during formation of the primary cell wall [72–75], whereas three types of CESA subunits (CESA4, CESA7 and CESA8) are required for secondary cell wall formation [74, 76, 77]. In addition, CESA2, CESA5, and CESA9 also function in primary cell wall synthesis [72]. This study identified 6 phosphoproteins as different CESAs in *B. distachyon*, namely BdCESA1, BdCESA2, BdCESA4, BdCESA7, BdCESA8 and BdCESA9 (Bradi2g34240.1, Bradi1g04597.1, Bradi2g49912.1, Bradi3g28350.1, Bradi1g54250.1 and Bradi4g30540.1 in Additional file 1: Table S1). Representative MS/MS spectra of phosphopeptides BdCESA2 and BdCESA4 are shown in Additional file 10: Figure S6, panel A and B, respectively. *AtCESA2* expressed in all organs except root hairs, and lack of *AtCESA2* expression can lead to stunted growth of hypocotyls in seedlings and greatly reduced seed production in mature plants [78]. All cellulose synthases described to date have shown conserved structural features [78]. The amino N-terminal of the CESA protein contains a RING finger domain with a strongly conserved CxxC motif (C = Cys, × = any amino acid) [79]. The RING finger domain is a small zinc-binding domain found in many functionally distinct proteins [80]. Kurek *et al.* [81] confirmed that GhCESA1 formed homodimers as well as heterodimers with GhCESA2 through a zinc-binding domain. Chu *et al.* [78] demonstrated that AtCESA2 could homodimerize and the zinc finger domain of AtCESA2 was important for the interaction. Sequence analyses indicated that all six identified BdCESAs contained a RING finger domain with a conserved CxxC motif (Additional file 10: Figure S6, panel C). There are 8 transmemberane regions in BdCESA1, BdCESA2, BdCESA8 and BdCESA9, but there is no the second transmemberane region in BdCESA4 and BdCESA7. All phosphorylation sites identified from these BdCESAs are located between the RING finger domain and transmemberane region I. Nühse *et al.* [8] performed a plasma membrane phosphoproteomics in *Arabidopsis* and revealed that CESA1, CESA3, and CESA5 proteins were phosphorylated at several sites clustered in the two hypervariable regions. Chen *et al.* [82] found that phosphorylation of CESA1 differentially affects polar interaction with microtubules and may regulate the length or quantity of a subset of cellulose microfibrils in the primary cell wall. The region with phosphorylation sites in each BdCESA belonged to hypervariable region I (Additional file 10: Figure S6, panel C). Phosphorylation of AtCESA7 was linked to its degradation via a 26S proteasome-dependent pathway [83]. It seems that phosphorylation of the 6 BdCESAs may play vital roles in cell expansion during rapid vegetative growth of *Brachypodium*.

(1, 3; 1, 4)-β-D-glucan is mainly detected in some families of the *Poales* [70]. Recently, various genes involved in (1, 3; 1, 4)-β-D-glucan synthesis were identified [84, 85]. They belong to two grass-specific families (CSLF and CSLH) of the cellulose synthase-like (CSL) gene superfamily. In the present study, two CSL proteins BdCSLF6 (Bradi3g16307.1) and BdCSLH1 (Bradi5g10130.1) were identified as phosphoproteins. In developing barley coleoptiles, the transcript levels of *HvCslF6* are maximal in 4- to 5-d coleoptiles, at the time when (1, 3; 1, 4)-β-D-glucan content of coleoptile cell walls also reaches its maximum level [84]. HvCSLH1 protein is responsible for β-glucan deposited in cell walls of transgenic *Arabidopsis* [85]. In *B. distachyon*, the *CSLH* gene showed a much higher transcript level and played a prominent role in (1, 3; 1, 4)-β-D-glucan synthesis [64].

## Conclusions

In this work, TiO_2_-enriched phosphopeptides in Bd21 seedling leaves detected by LC-MS/MS, and MaxQuant software were used to identify the phosphoproteins and phosphorylation sites. To our knowledge, this is the first large-scale phosphoproteome analysis in *B. distachyon* and provides an overview of *in vivo* phosphorylation events in seedling leaves. A total of 950 phosphoproteins containing 1470 unambiguous phosphorylation sites were identified. In-depth GO, conservation, and KEGG pathway analyses of these phosphoproteins revealed the phosphorylation profiling during rapid seedling leaf growth. GO analysis showed that the 950 phosphoproteins mainly functioned in protein binding, nucleotide binding and kinase activity. Conservation analysis revealed that 887 (93.4%) of the phosphoproteins had phosphorylation orthologs in other species. Many fundamental biological pathways and two signal transduction pathways were identified through KEGG pathway analysis.

Fifty eight TFs, 94 PKs and 8 PPs were identified from the large dataset and analyzed in depth. Thirteen enriched motifs specific for various Ser/Thr PKs were identified from *B. distachyon*. A total of 127 phosphoproteins contained multiple phosphorylation sites and 91 of them contained two or more phosphorylation motifs. Several signal transduction and cell wall expansion associated phosphoproteins that may have vital functions in the rapid growth of *B. distachyon* seedling leaves were found such as B-ARR, PTEN, CESA and CSL. Our overall results provide an important resource for further research and new insights into the regulation of protein post-translational phosphorylation during rapid vegetative growth of *Poaceae* species.

## Methods

### Plant materials

Seeds of *Brachypodium distachyon* Bd21, kindly provided by Dr. John Vogel, USDA-ARS, Albany, CA, were surface sterilized in 5% sodium hypochlorite for 5 min, and rinsed 4 times in sterile distilled water. Seeds were submerged in water for 12 h at room temperature, and then transferred to wet filter paper for 24 h to germinate at room temperature (22–25°C). Uniformly germinated seeds were selected and grown in three plastic pots containing Hoagland’s solution [86]; the Hoagland’s solution was changed every two days. At the three leaf stage, all three leaves were collected and frozen at –80°C. Culture of the Bd21 seedlings was repeated three times and the leaves were collected independently.

### Protein extraction

Total proteins were extracted from seedling leafs according to the method of Wang *et al.* [87] with minor modifications. Approximately 400 mg fresh leaves of each sample were ground into fine powder in liquid nitrogen. The ground powder was suspended in 4 mL SDS buffer (30% sucrose, 2% SDS, 100 mM Tris-HCl, pH 8.0, 50 mM EDTA-Na_2_, 20 mM DTT) and 4 mL phenol (Tris-buffered, pH 8.0) in a 10 mL tube, and 1 mM phenylmethanesulfonyl fluoride (PMSF) and PhosSTOP Phosphatase Inhibitor Cocktail (Roche, Basel, Switzerland), were added to inhibit the activities of proteases and phosphatases. The mixtures were thoroughly vortexed thoroughly for 30 s and the phenol phase was separated by centrifugation at 14,000 × *g* and at 4°C for 15 min. The upper phenol phase was pipetted to fresh 10 mL tubes and four volumes of cold methanol plus 100 mM ammonium acetate were added, and the mixture was stored at –20°C for at least 30 min. After centrifugation at 14,000 × *g* and at 4°C for 15 min, the supernatant was carefully discarded and the precipitated proteins were washed twice with cold methanolic ammonium acetate (100 mM) and ice-cold 80% acetone, respectively. Finally the pellet was vacuum-dried and then dissolved in lysis buffer (7 M urea, 2 M thiourea, 4% w/v CHAPS and 65 mM DTT) over 3 h at 4°C. The protein mixtures were harvested by centrifugation at 14,000 × *g* and 4°C for 15 min to remove insoluble materials. The concentrations of the extracted protein mixtures were determined with a 2-D Quant Kit (Amersham Bioscience, Buckinghamshire, UK) using BSA (2 mg/mL) as standard, and the final protein solution was stored at –80°C for later use.

### Phosphopeptide enrichment using TiO_2_ microcolumns

Extracted protein mixtures were directly reduced with dithiothreitol (DTT), alkylated with iodoacetamide, and subsequently digested with endoproteinase Lys-C and trypsin as previously described [88]. The enrichment procedure for phosphopeptides was performed as reported by Wu *et al.* [5] with modifications. The TiO_2_ beads (GL Sciences, Tokyo, Japan) were incubated in 400 *μ*L loading buffer containing 65% Acetonitrile (ACN)/2% trifluoroaceticacid (TFA)/saturated by glutamic acid. A total of 2 mg of tryptic peptides were dissolved in 600 *μ*L loading buffer, and incubated with an appropriate amount of TiO_2_ beads. After washing with 600 *μ*L wash buffer (65% ACN/0.1% TFA), phosphopeptides were eluted twice with 300 *μ*L elution buffer (500 mM NH_4_OH/60% ACN) and the eluates were dried down and reconstituted in 0.1% formic acid (FA)/H_2_O for MS analysis.

### Phosphopeptide identification and phosphorylation site localization using LC-MS/MS

Enriched phosphopeptides were separated on a self-packed C18 reverse phase column (75 *μ*m I.D., 150 mm length) (Column Technology Inc., Fremont, CA), which directly connected the nano electrospray ion source to a LTQ-Orbitrap XL mass spectrometer (Thermo Fisher Scientific, San Jose, CA). Pump flow was split to achieve a flow rate at 1 *μ*L/min for sample loading and 300 nL/min for MS analysis. The mobile phases consisted of 0.1% FA (A), and 0.1% FA and 80% ACN (B). A five-step linear gradient of 5% to 30% B in 105 min, 35% to 90% B in 16 min, 90% B in 4 min, 90% to 2% B for 0.5 min and 2% B for 14.5 min was performed. The spray voltage was set to 2.0 kV and the temperature of the heated capillary was 240°C.

For data acquisition, each MS scan was acquired at a resolution of 60,000 (at 400 m/z) with lock mass option enabled and was followed by a data-dependent top 10 MS/MS scans using collision induced dissociation (CID). The threshold for precursor ion selection was 500 and mass window for precursor ion selection was 2.0 Da. The dynamic exclusion duration was 120 s, repeat count was 1 and repeat duration was 30 s. The analyzer for the MS scans was Orbitrap and for the MS/MS scans LTQ (37% relative collision energy). Three biological replicates were performed independently from sample collection to phosphopeptide identification using LC-MS/MS.

The raw files were processed with MaxQuant (version 1.2.2.5) [89] and searched against the *B. distachyon* protein database (31,029 entries in total) in Phytozome (http://www.phytozome.net/search.php; version 9.1) concatenated with a decoy of reversed sequences. The following parameters were used for database searches: cysteine carbamidomethylation was selected as a fixed modification; methionine oxidation, protein N-terminal acetylation, and phosphorylation on serine, threonine and tyrosine were selected as variable modifications. Up to two missing cleavage points were allowed. The precursor ion mass tolerances were 7 ppm, and fragment ion mass tolerance was 0.5 Da for MS/MS spectra. The false discovery rate (FDR) was set to < 1.0% for both peptide and protein identifications, the minimum peptide length was set to 6.

Phosphorylation site localization was based on PTM scores that assign probabilities for each of the possible sites according to their site-determining ions. In this study, MaxQuant (version 1.2.2.5) was used to calculate PTM scores and PTM localization probabilities. Potential phosphorylation sites were then grouped into three categories depending on their PTM localization probabilities [10, 37, 88], namely class I (localization probability, P ≥ 0.75), class II (0.75 > P ≥ 0.5) and class III (P < 0.5). A false discovery rate (FDR) of 1% was used for phosphorylation sites identification.

### Bioinformatics

The GO biological processes, molecular functions and cellular components of the identified phosphoproteins were examined by using Blast2GO software [90]. GO enrichment analysis was conducted with agriGO [91]. The statistical test method was set as Fisher and the multi-test adjustment method was set as Bonferroni. Pfam domain information was extracted from the database (http://pfam.sanger.ac.uk/search) [92]. Protein descriptions were extracted from the *B. distachyon* protein database (31,029 entries in total) in Phytozome (http://www.phytozome.net/search.php; version 9.1). For pathway analysis, the proteins were searched in the KEGG *B. distachyon* database and mapped to *B. distachyon*-specific pathways with KEGG Mapper. The enrichment of TF family significantly regulated by phosphorylation was performed using a two-sided test of the hypergeometric distribution. The significantly enriched phosphorylation motifs were extracted from phosphopeptides with unambiguous phosphorylation sites (class I) using the Motif-X algorithm [33]. The phosphopeptides were centered at phosphorylated amino acid residues and aligned, and six positions upstream and downstream of the phosphorylation site were included. In the cases of C- and N-terminal peptides, the sequence was completed to 13 amino acids with the required number of “X”s, where X represents any amino acid. Because the upload restriction of Motif-X is 10 MB, a fasta format dataset (nearly 10 MB) containing the protein sequences from the *B. distachyon* protein database in Phytozome (version 9.1) was used as the background database to normalize scores against random distributions of amino acids. The occurrence threshold was set to 5% of the input data set at a minimum of 20 peptides, and the probability threshold was set to P < 10^-6^. The sequences of all the phosphoproteins were used for BLAST analysis with the National Center for Biotechnology Information (NCBI) clusters of the KOG database to obtain the KOG numbers of those proteins. Then a dataset containing all the KOG numbers was used for protein-protein interaction (PPI) analysis by using the STRING database (version 9.1, http://string-db.org) [93]. Only interactions that had a confidence score of at least 0.9 were used to construct the network and the networks were then displayed using Cytoscape (version 3.0.2) software [94]. HMMTOP (version 2.0, http://www.enzim.hu/hmmtop/) was used to predict the transmembrane helices in proteins [95].

## Electronic supplementary material

Additional file 1: Table S1: Phosphorylation sites and phosphoproteins identified from seedling leaves of *B. distachyon*. (A) Total phosphorylation sites and phosphoproteins. (B) Annotation of the phosphoproteins. (XLSX 506 KB)

Additional file 2: Figure S1: Representative tandem mass spectrometry spectra of the seven phosphopeptides containing the eight phosphosites in Bradi1g66870.1. (A) KHLPSQDITDAS(ph)GDEEEGSR. (B) HWS(ph)PSAGRR. (C) LKDFS(ph)ADPELNK. (D) MNYLGT(ph)PPS(ph)DLEK. (E) NS(ph)PISIK. (F) QFDS(ph)PDDSLVK. (G) RIS(ph)PPSGR. (PDF 275 KB)

Additional file 3: Table S2: Conservation analysis of phosphoproteins identified from *B. distachyon*. (XLSX 122 KB)

Additional file 4: Figure S2: GO biological process enrichment of 582 highly conserved phosphoproteins. The statistical significance of the enrichment analysis is represented by a scale of red tones whose intensity is proportional to the degree of significance starting from FDR < 0.05. (PDF 382 KB)

Additional file 5: Table S3: Pathway analysis of the identified phosphoproteins by KEGG. (A) Pathway search results of all identified phosphoproteins and highly conserved phosphoproteins. (B) KEGG identifiers of all the identified phosphoproteins. (XLSX 23 KB)

Additional file 6: Figure S3: Pathway mapping of identified phosphoproteins by KEGG. Phosphoproteins were analyzed in the KEGG *B. distachyon* pathway database. The phosphoproteins were used to search the KEGG *B. distachyon* database and mapped to *B. distachyon* specific pathways with KEGG Mapper. In each pathway map, objects with red foreground color and blue background color represent the highly conserved phosphoproteins; objects with pink foreground and blue background represent the other conserved phosphoproteins; objects with yellow foreground and blue background represent proteins identified as novel phosphoproteins; objects with green background represent the total proteins in the *B. distachyon* KEGG database. Details of the pathways can be found in Additional file 5: Table S3. (PDF 1 MB)

Additional file 7: Table S4: Enriched phosphorylation motifs, and the phosphopeptides and the corresponding phosphoproteins in each phosphorylation motif. (A) Phosphorylation motifs enriched by Motif-X and the putative protein kinases. (B) Phosphopeptides and the corresponding phosphoproteins in each phosphorylation motif. (C) Motifs of phosphoproteins with multiple phosphorylation sites. (XLSX 60 KB)

Additional file 8: Figure S4: GO enrichment of phosphoproteins represented by the phosphorylation motifs in *B. distachyon.* GO enrichment graphs of phosphoproteins contained each of 11 motifs were displayed by Blast2GO software. No GO terms were significantly enriched from phosphoproteins containing motif 7 or motif 11. The statistical significance of the enrichment analysis is represented by a scale of red tones whose intensity is proportional to the degree of significance starting from p < 0.05. (PDF 2 MB)

Additional file 9: Figure S5: Protein-protein interaction network of the phosphoproteins. The confidence (score) was set as the highest (0.900) and only the phosphoproteins identified in this study were used to construct the interaction profiles. Protein kinases and protein phosphatases are highlighted in red and green respectively and other phosphoproteins identified in this study are shown with sky-blue nodes. (PDF 630 KB)

Additional file 10: Figure S6: Phosphorylation sites of the six BdCESAs. (A) and (B) The tandem mass spectrometry spectra of the phosphopeptides EFSGS(ph)LGNVAWK from BdCESA2 (Bradi1g04597.1) and VTIASQLS(ph)DR from BdCESA4 (Bradi2g49912.1). (C) Sequence alignment among the six BdCESAs. Phosphorylation sites identified in this study are highlighted in red. The RING domain and transmembrane regions are highlighted in yellow and green, respectively. (PDF 1 MB)

## Abbreviations

LC-MS/MS: Liquid chromatography-tandem mass spectrometry
GO: Gene ontology
KEGG: Kyoto Encyclopedia of Genes and Genomes
PK: Protein kinase
PP: Protein phosphatase
TF: Transcription factor
MAPK: Mitogen-activated protein kinase
CDK: Cyclin-dependent kinase
GHK: Growth-associated histone kinase
CDC2: Cell division cycle 2
CaMK: Calcium/calmodulin-dependent protein kinase
CHK1: C-terminal Src Kinase-homologous Kinase 1
CK: Casein kinase
PPI: Protein-protein interaction
STRING: Search Tool for Retrieval of Interacting Genes/Proteins
KOG: EuKaryotic Orthologous Group
MAP2K: Mitogen-activated protein kinase kinase
MEKK: Mitogen-activated protein kinase kinase kinase
RLK: Receptor-like protein kinase
WAK: Wall-associated receptor kinase
CESA: Cellulose synthase
CSL: Cellulose synthase-like.
