# Supplementary figures and images for: Large-scale phosphoproteome analysis in seedling leaves of Brachypodium distachyon L
Source: BMC Genomics. 2014 May 16;15(1):375. doi: 10.1186/1471-2164-15-375 (PMC4079959; doi:10.1186/1471-2164-15-375)

Figure S1

A

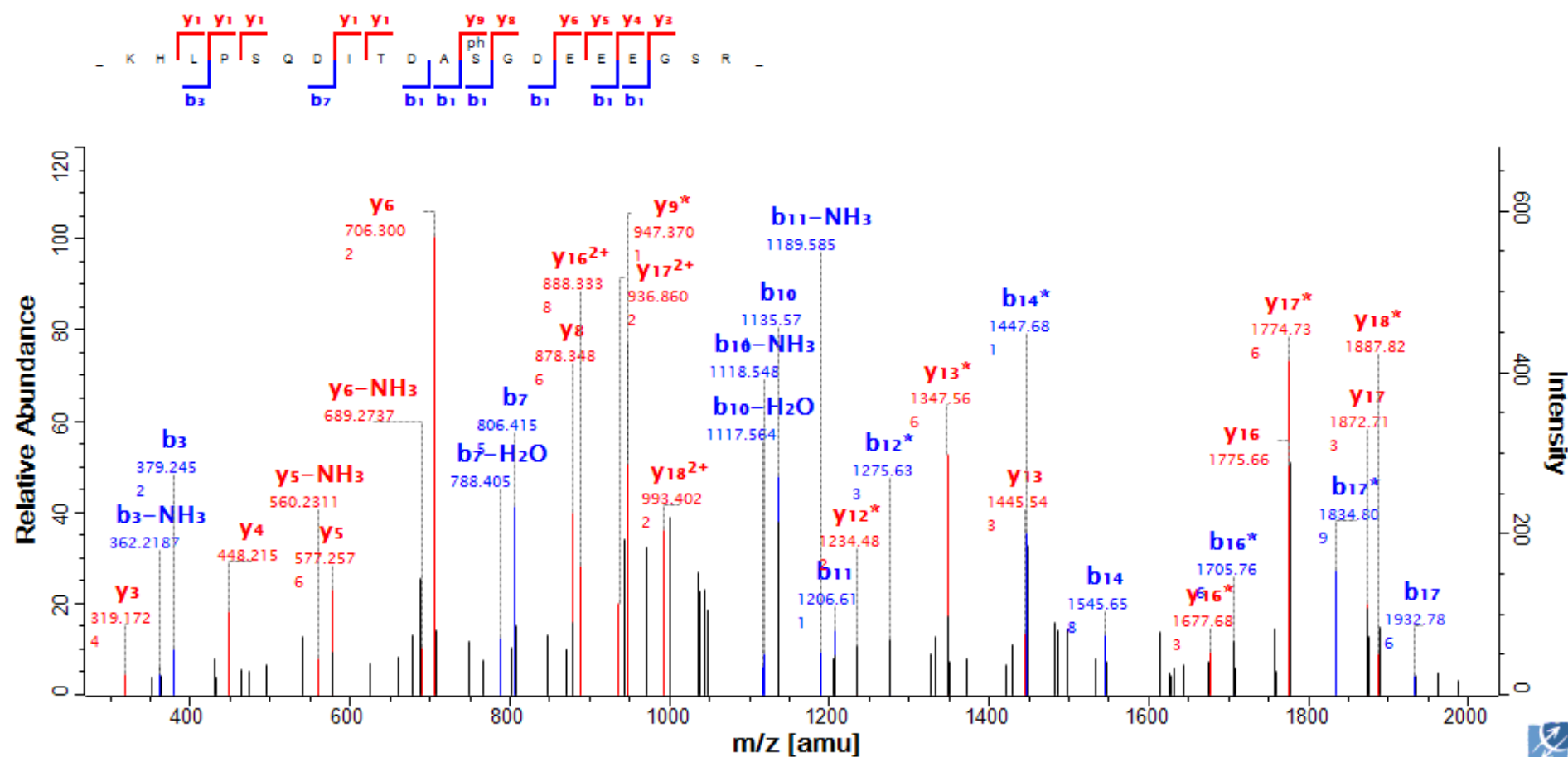

B

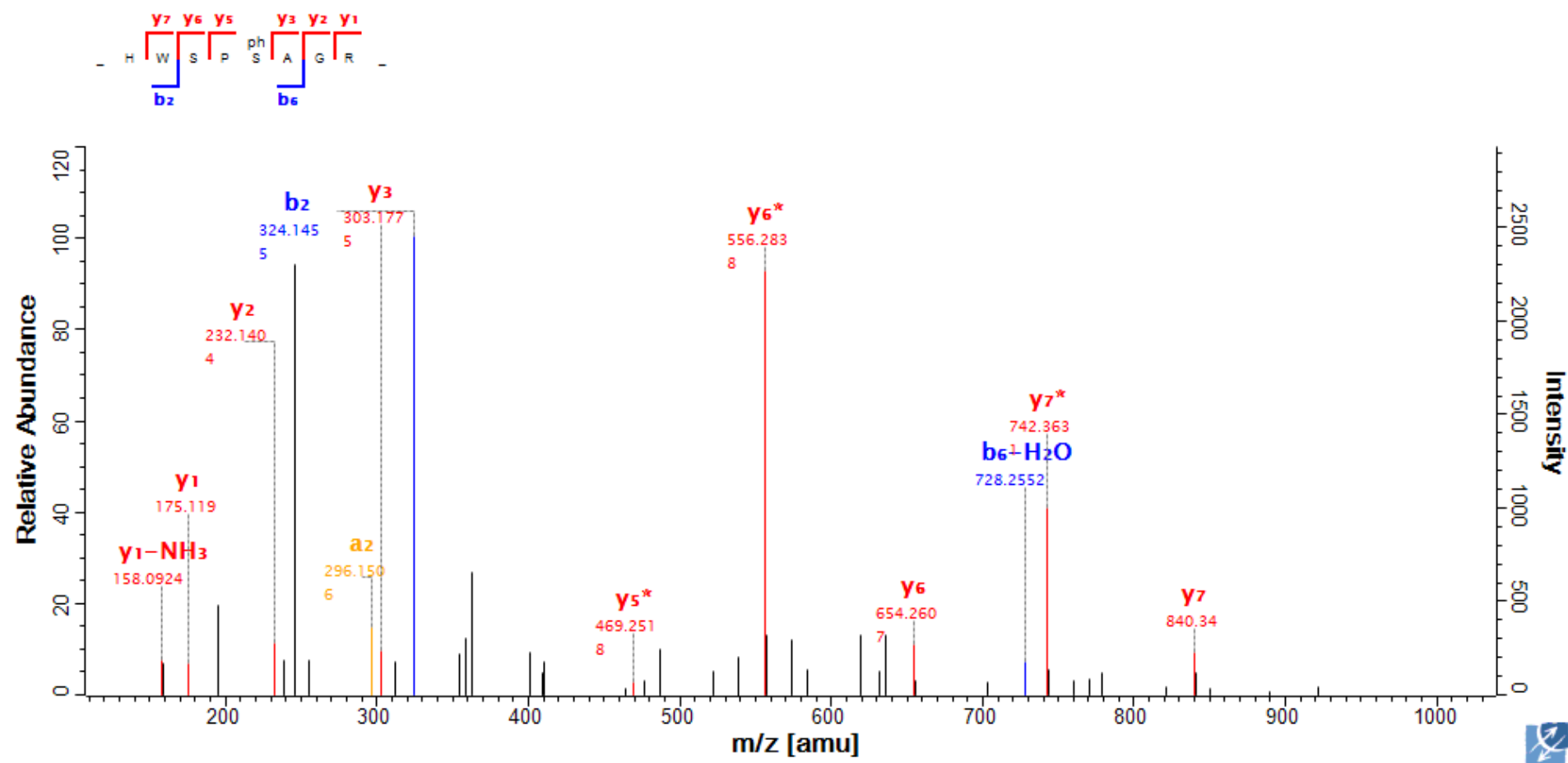

C

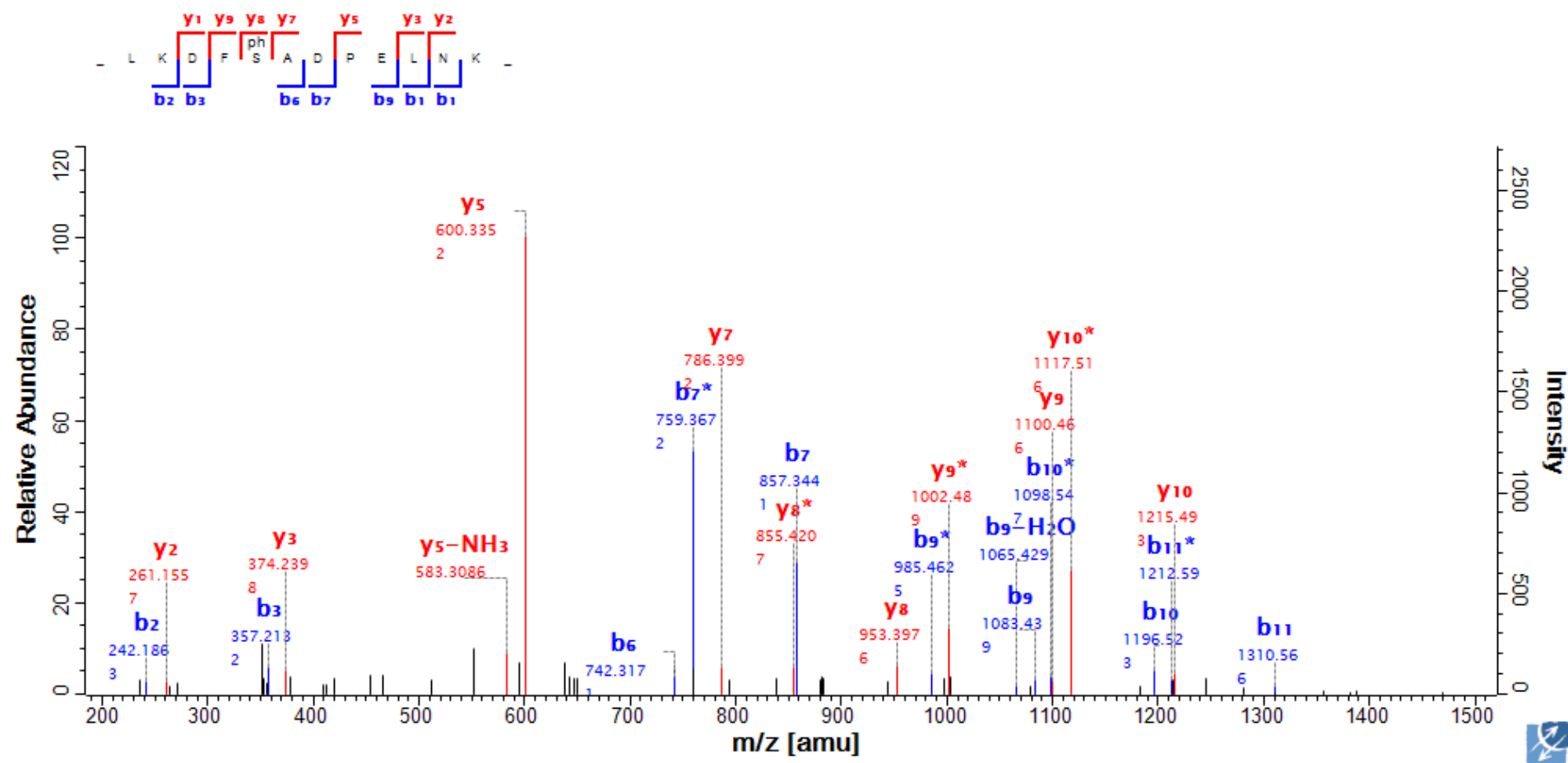

D

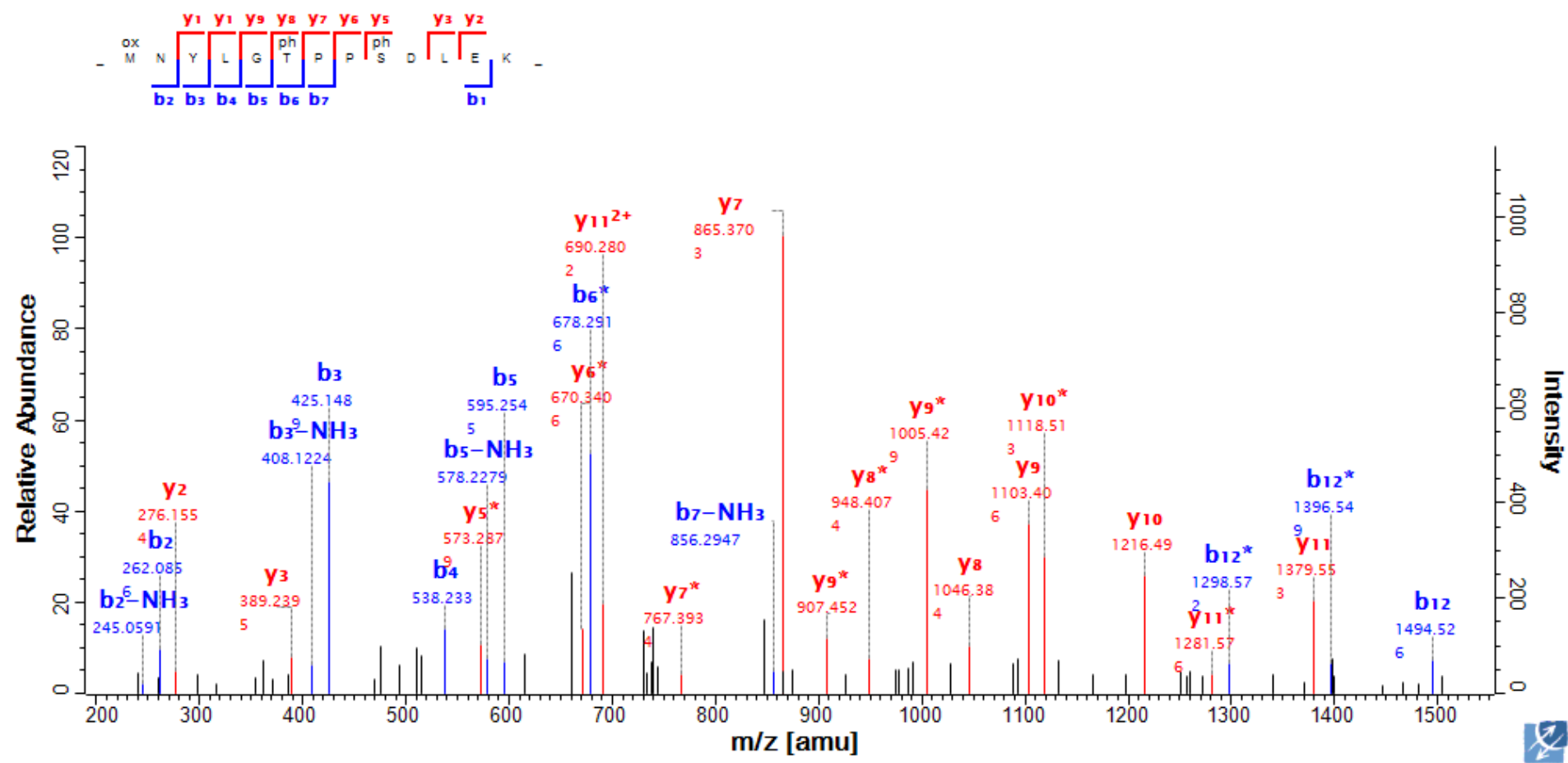

E

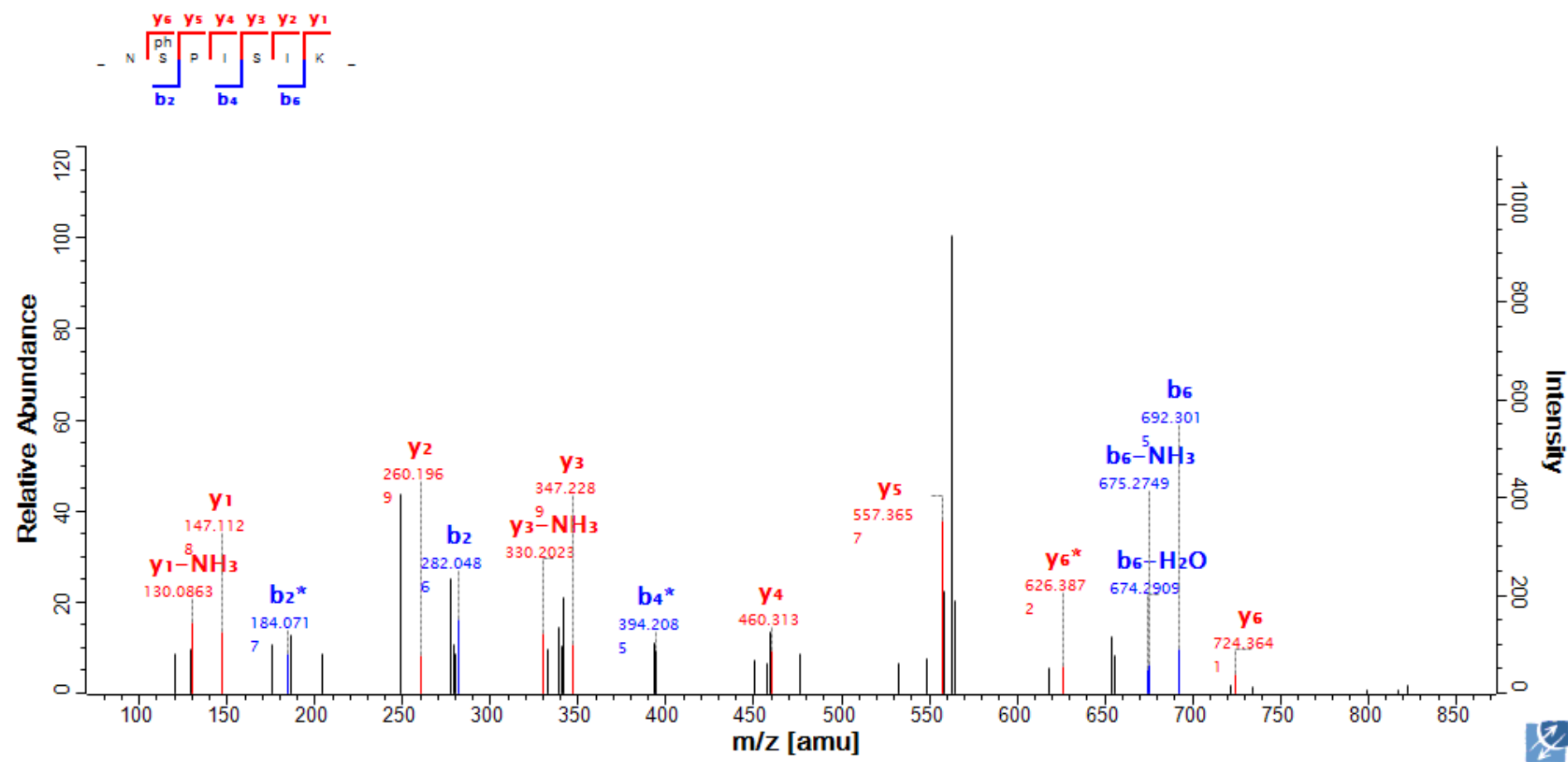

F

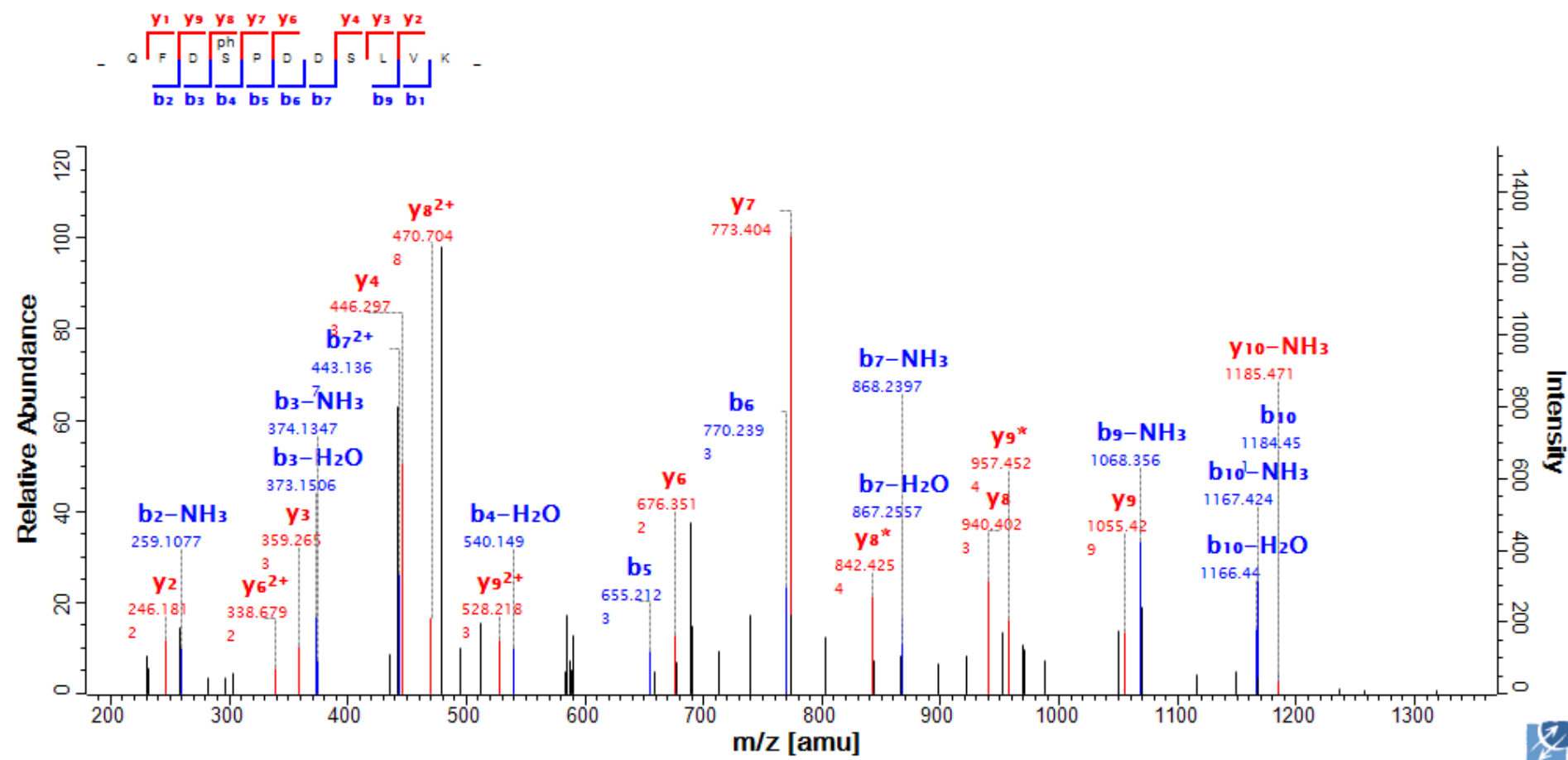

G

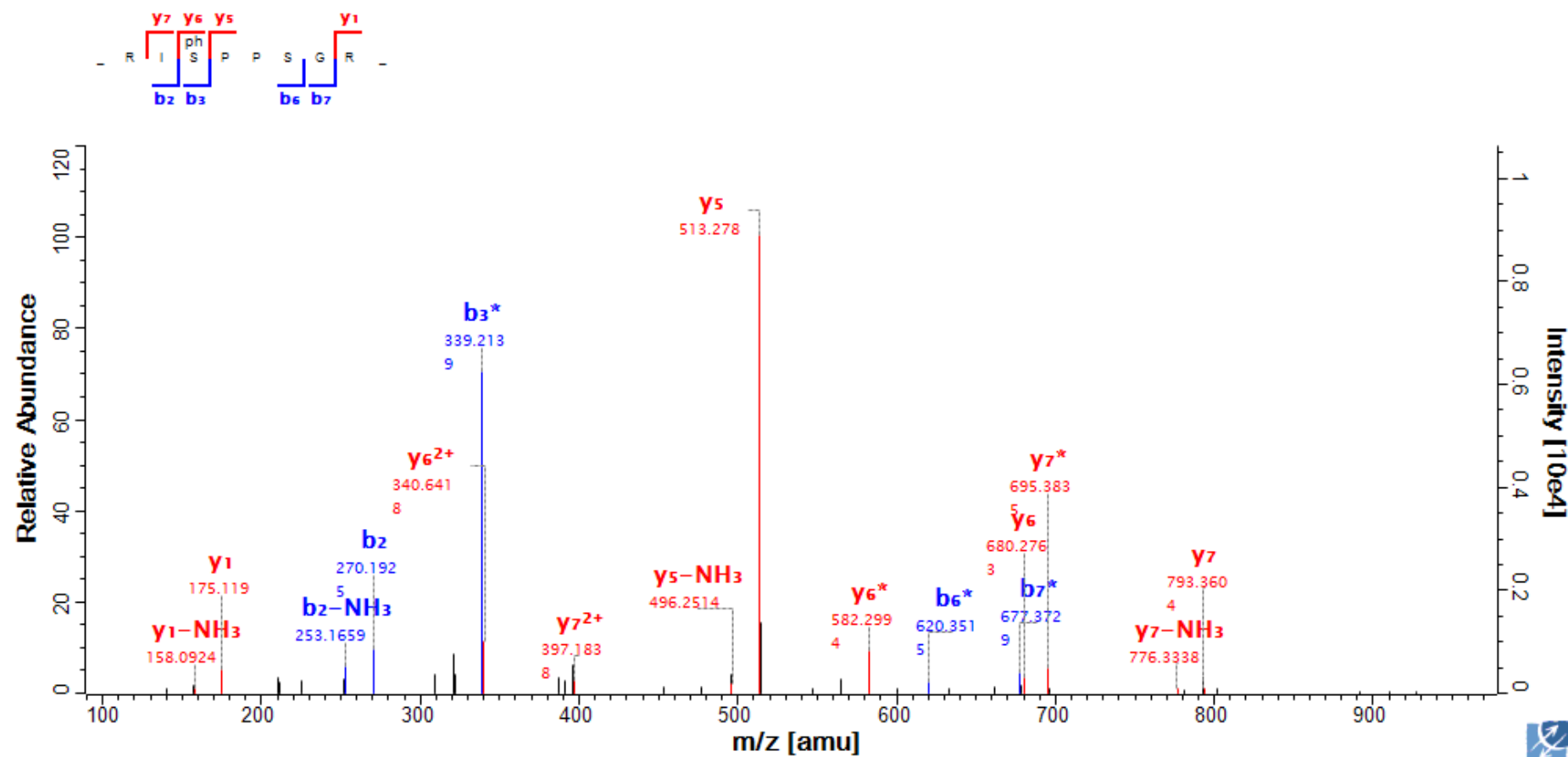

Supplement: Supplementary file 2 — Additional file 2: Figure S1: Representative tandem mass spectrometry spectra of the seven phosphopeptides containing the eight phosphosites in Bradi1g66870.1. (A) KHLPSQDITDAS(ph)GDEEEGSR. (B) HWS(ph)PSAGRR. (C) LKDFS(ph)ADPELNK. (D) MNYLGT(ph)PPS(ph)DLEK. (E) NS(ph)PISIK. (F) QFDS(ph)PDDSLVK. (G) RIS(ph)PPSGR. (PDF 275 KB) [file 12864_2014_6177_MOESM2_ESM.pdf]

Figure S2

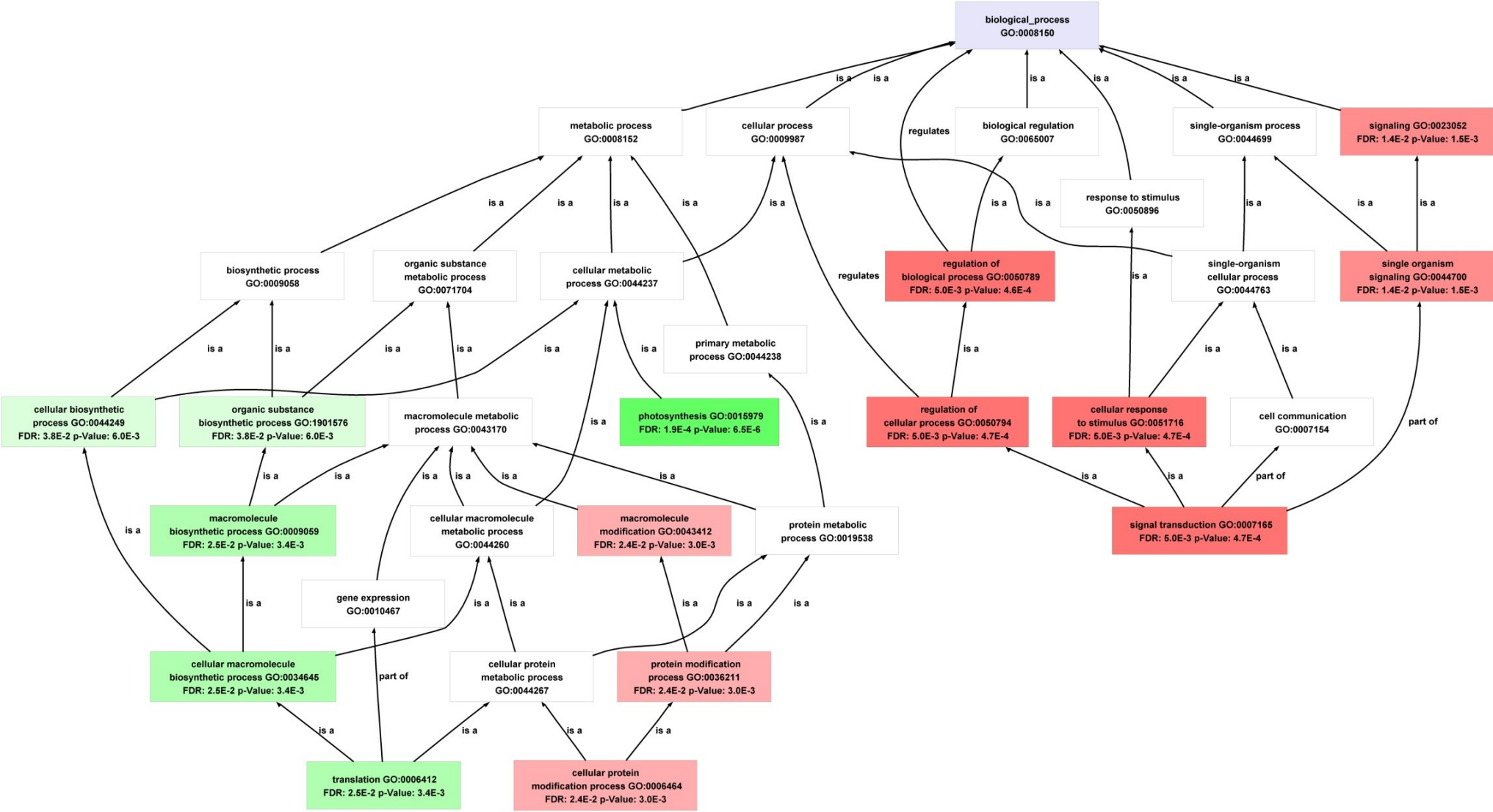

Supplement: Supplementary file 4 — Additional file 4: Figure S2: GO biological process enrichment of 582 highly conserved phosphoproteins. The statistical significance of the enrichment analysis is represented by a scale of red tones whose intensity is proportional to the degree of significance starting from FDR < 0.05. (PDF 382 KB) [file 12864_2014_6177_MOESM4_ESM.pdf]

Figure S4

Motif 1

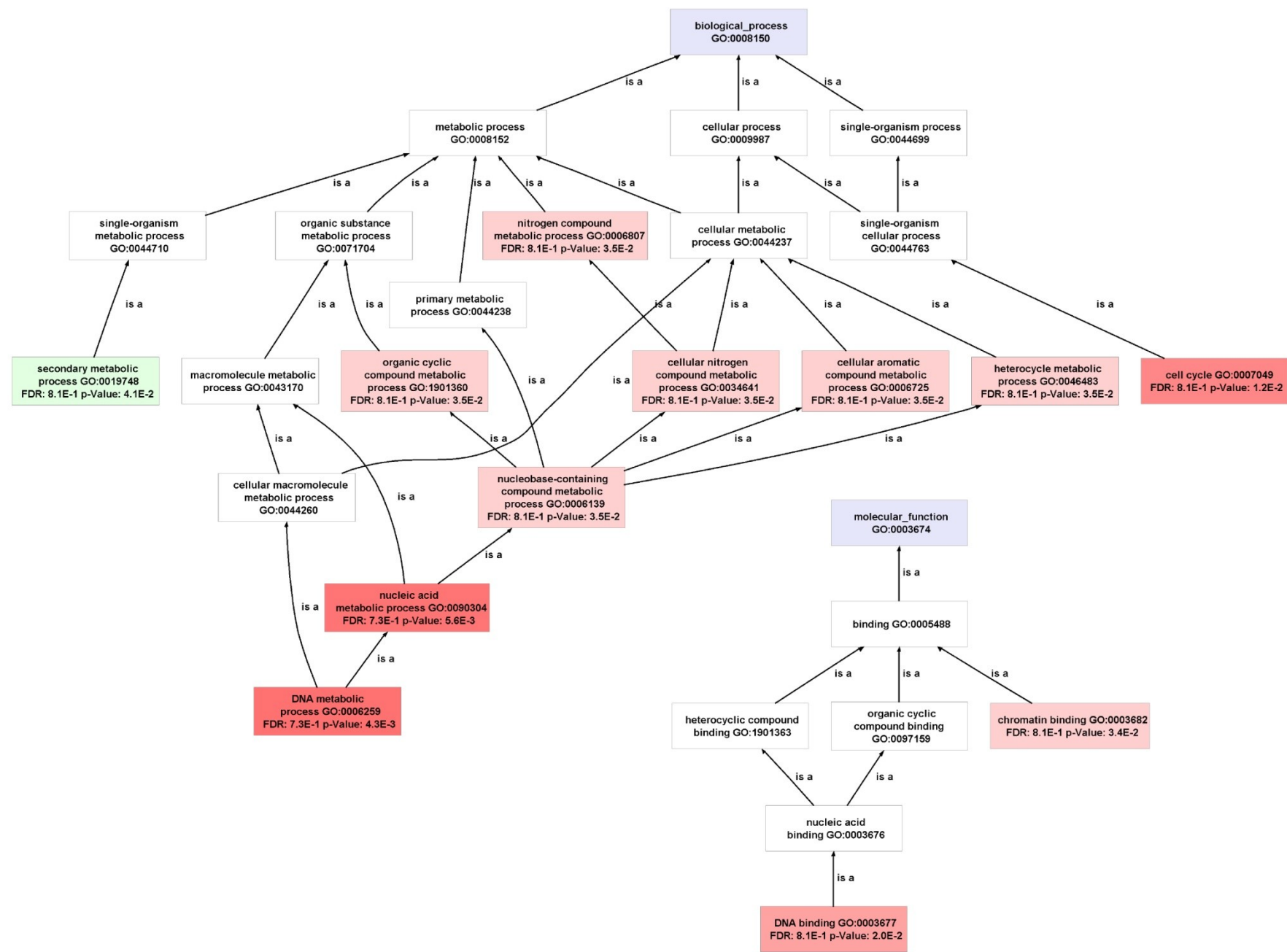

Motif 2

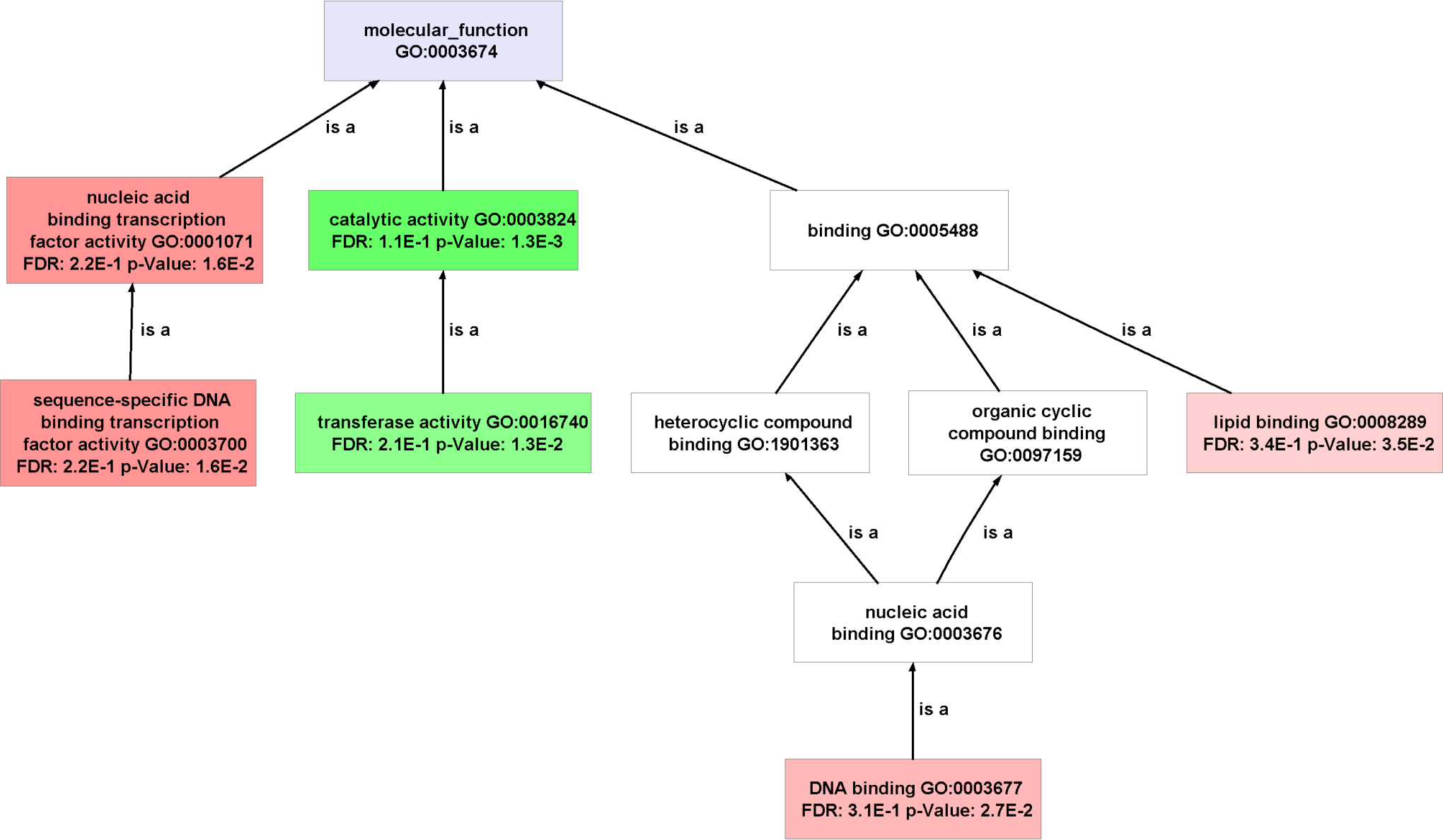

### Motif 3

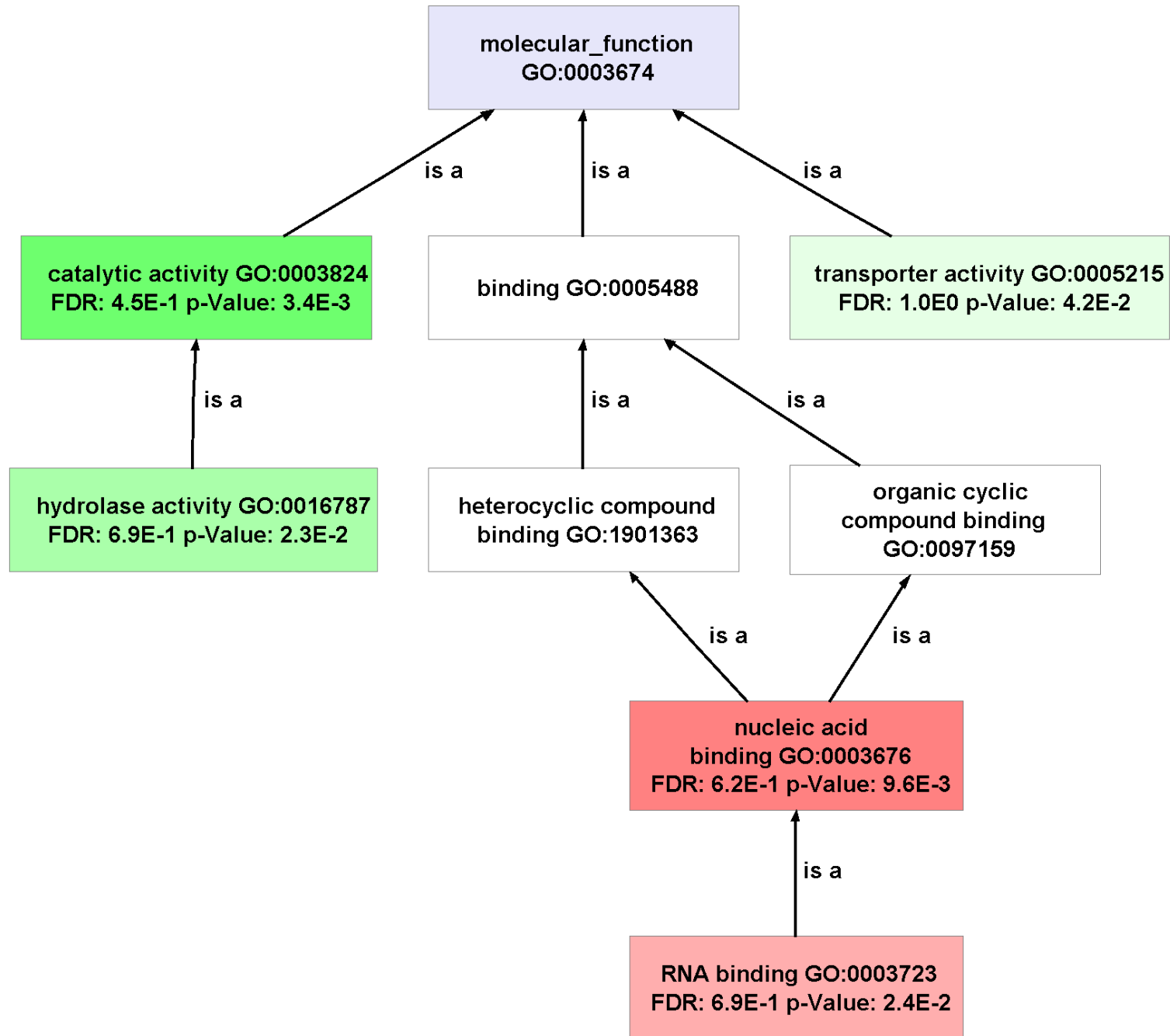

#### Motif 4

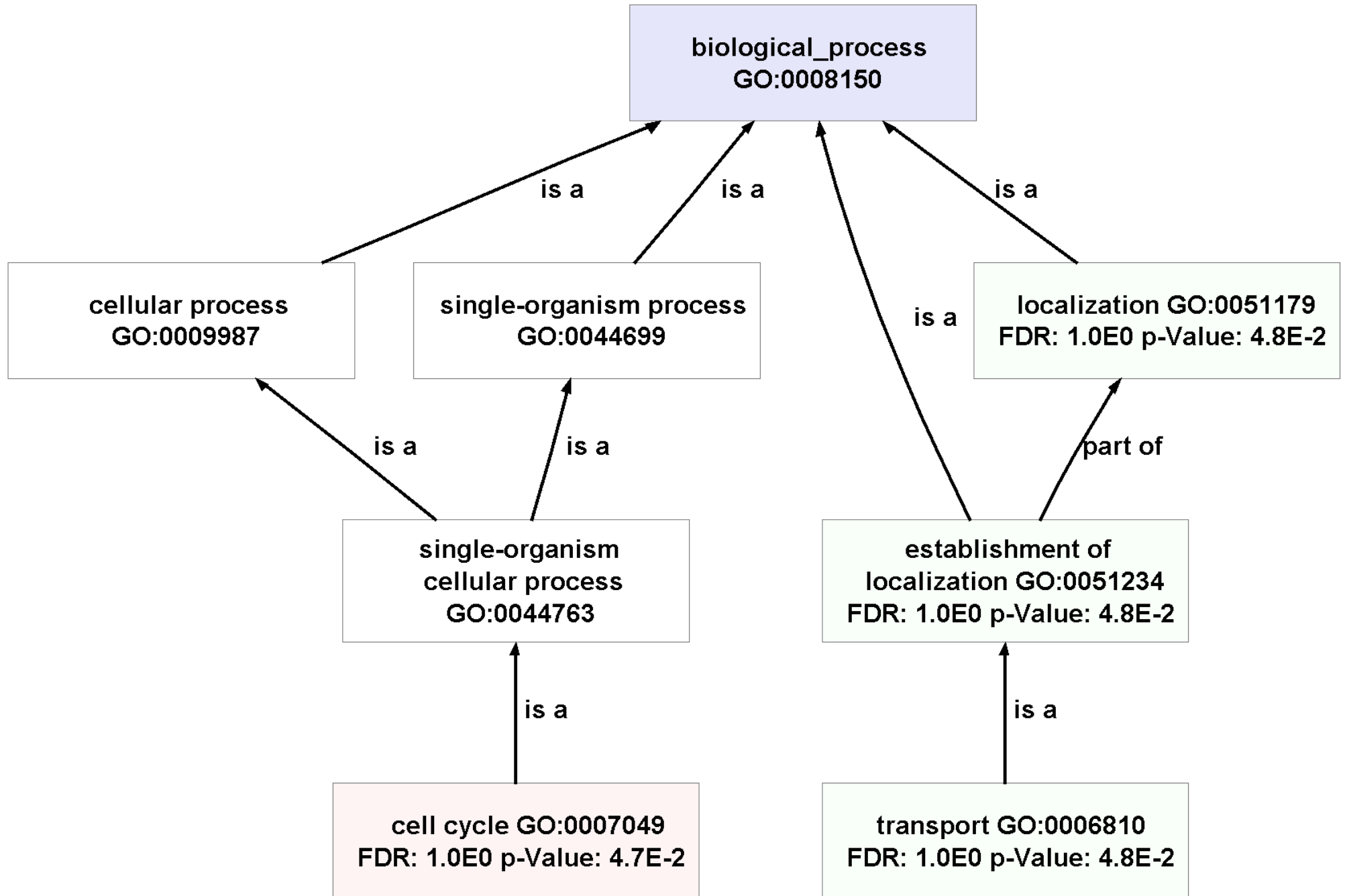

**Motif 5**

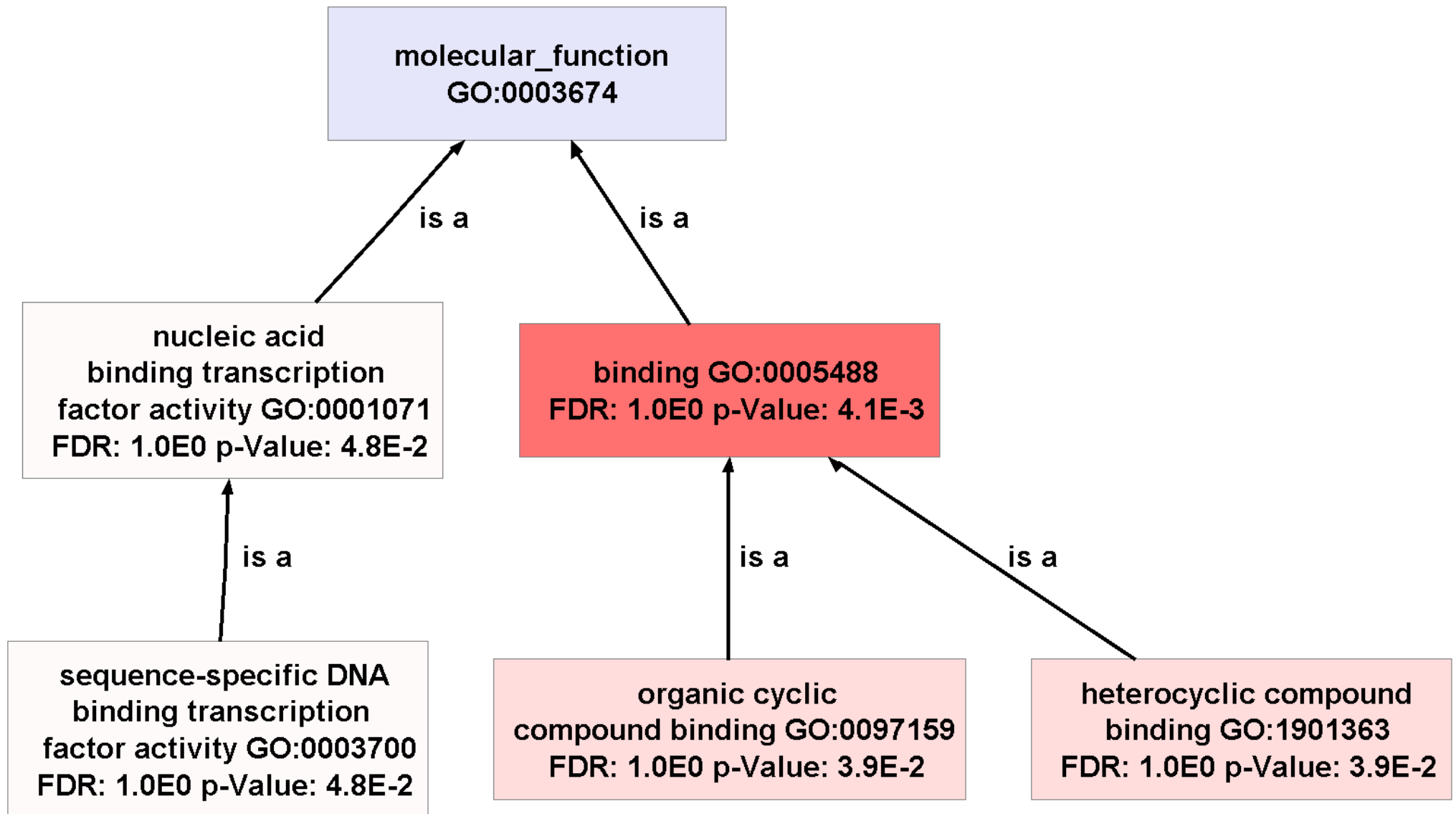

Motif 6

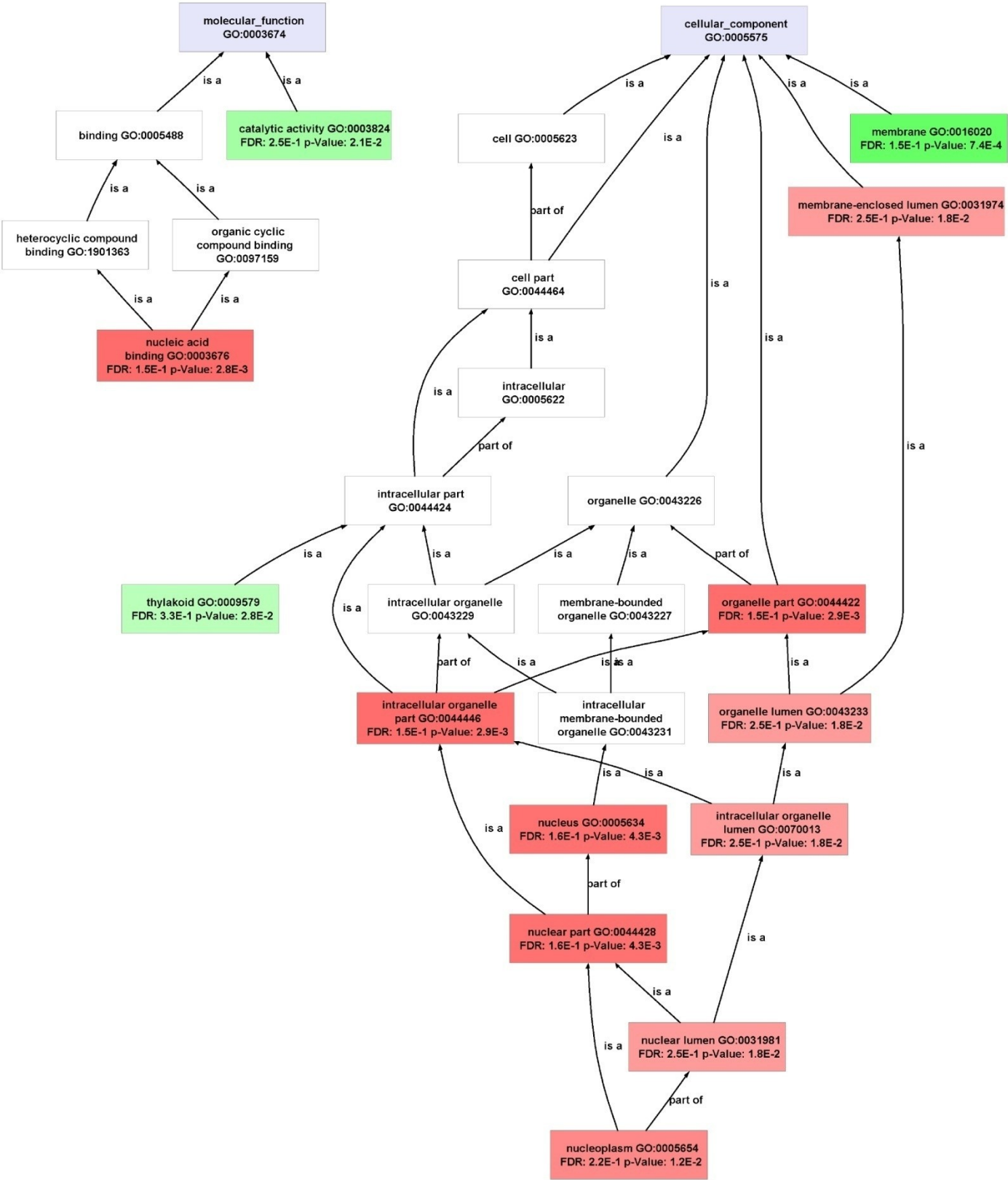

## Motif 8

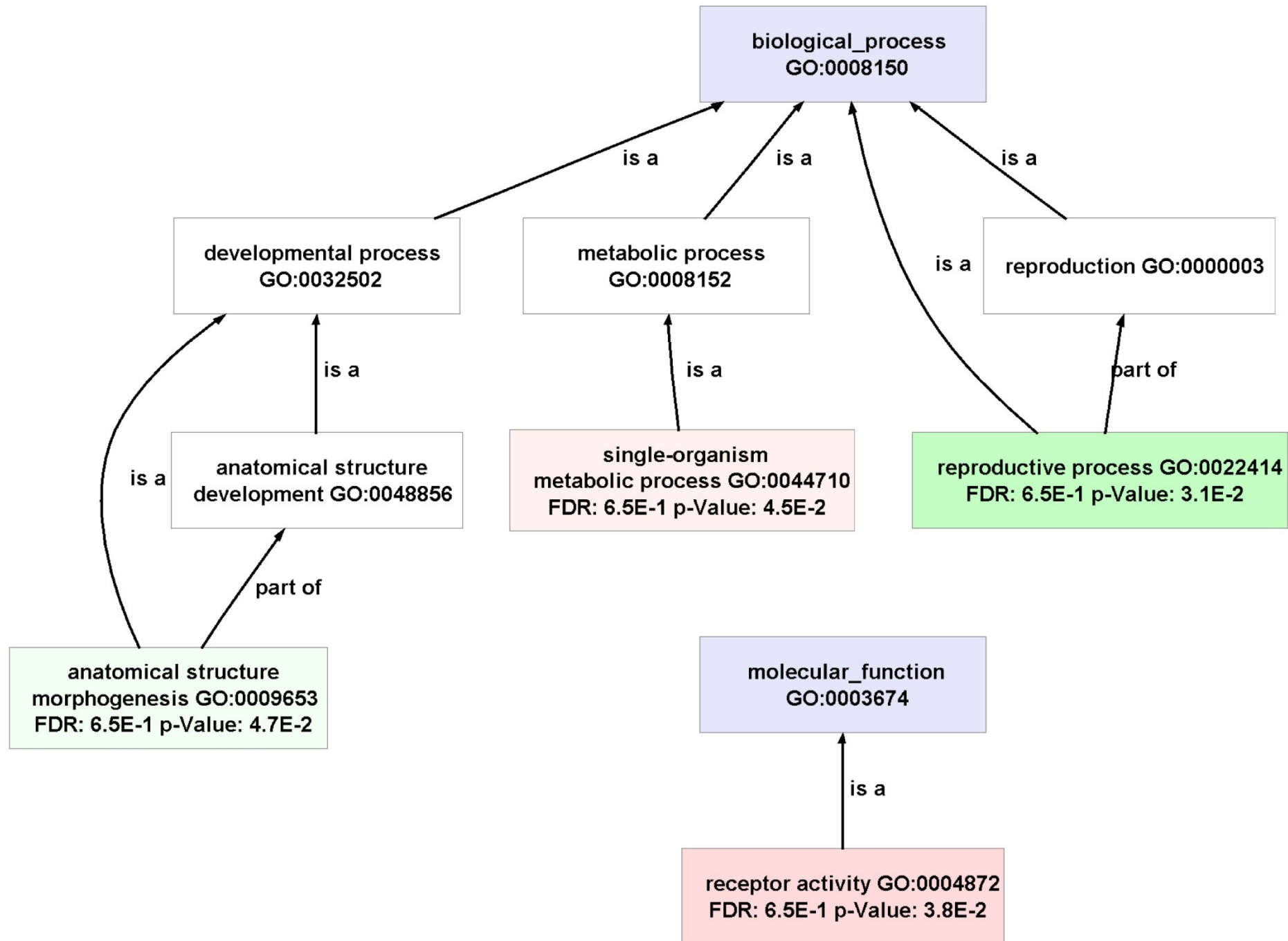

Motif 9

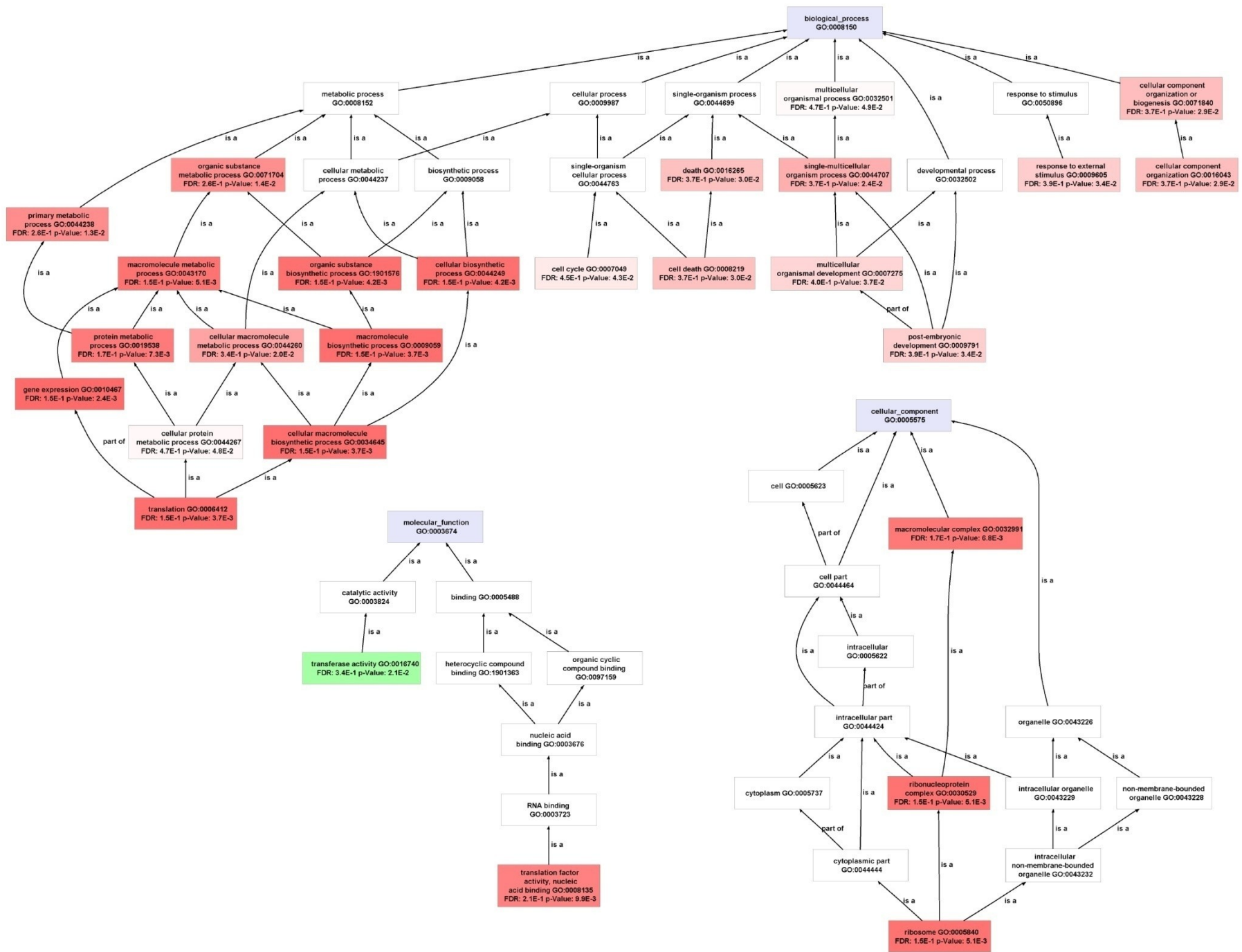

Motif 10

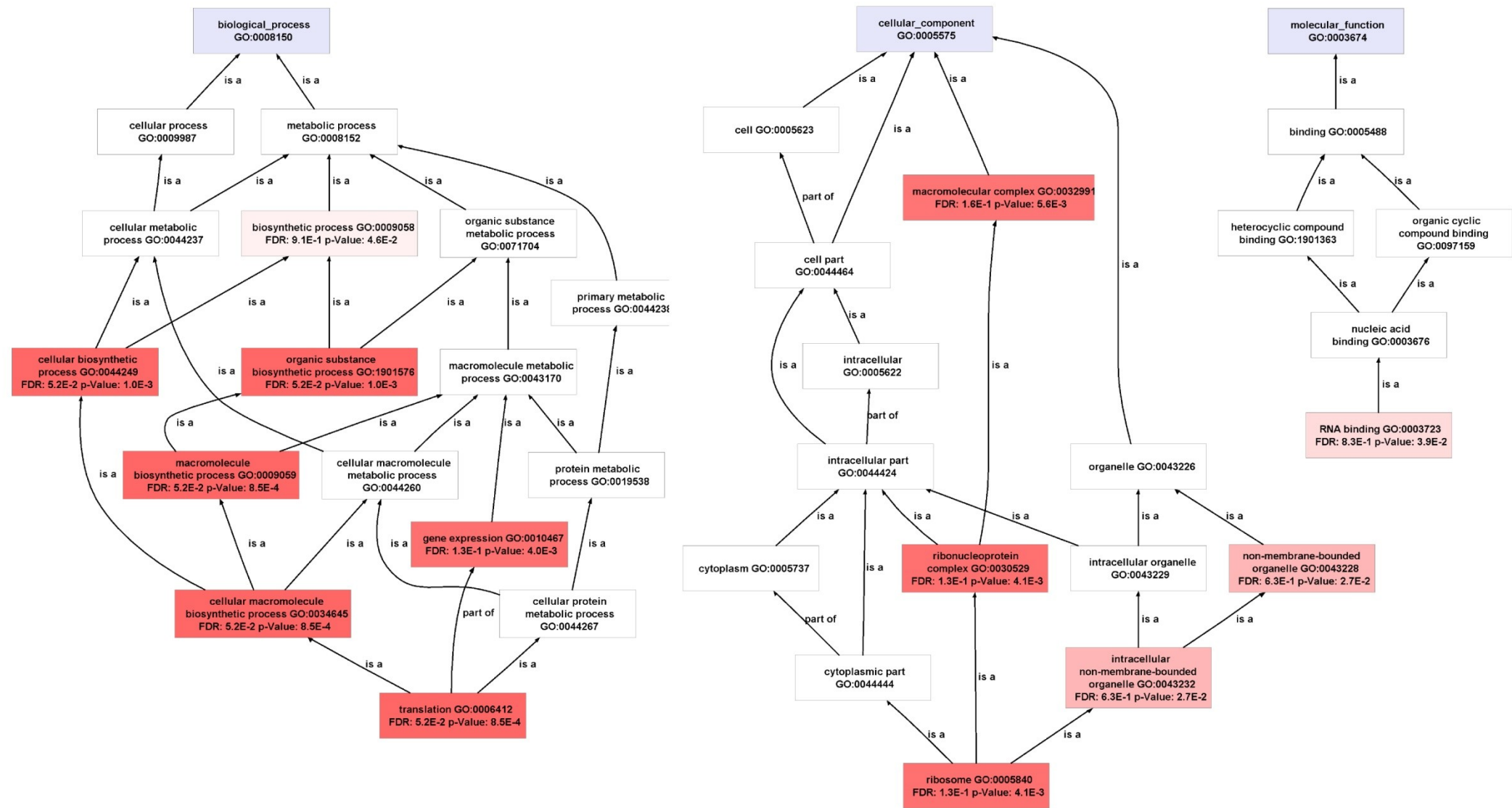

## Motif 12

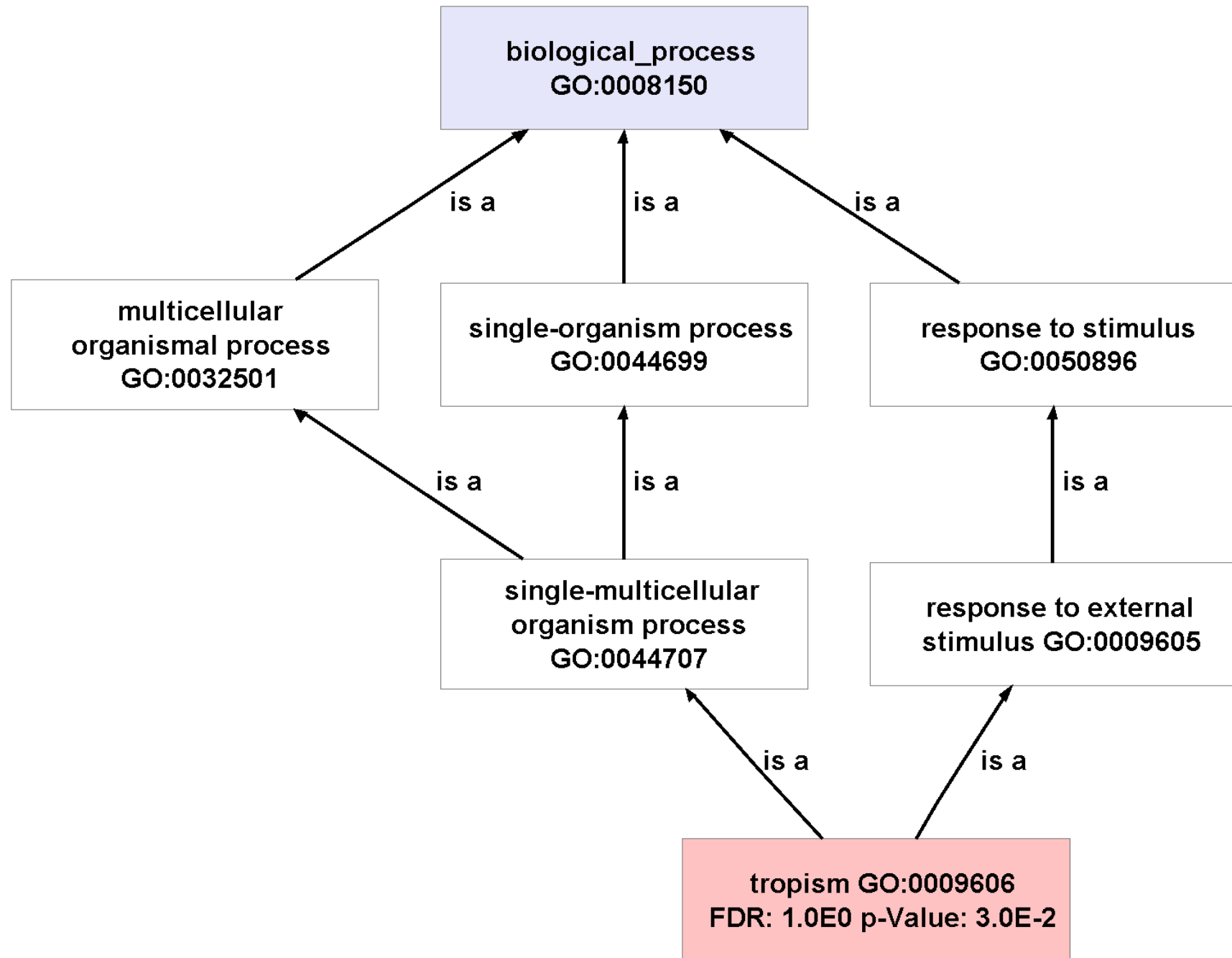

## Motif 13

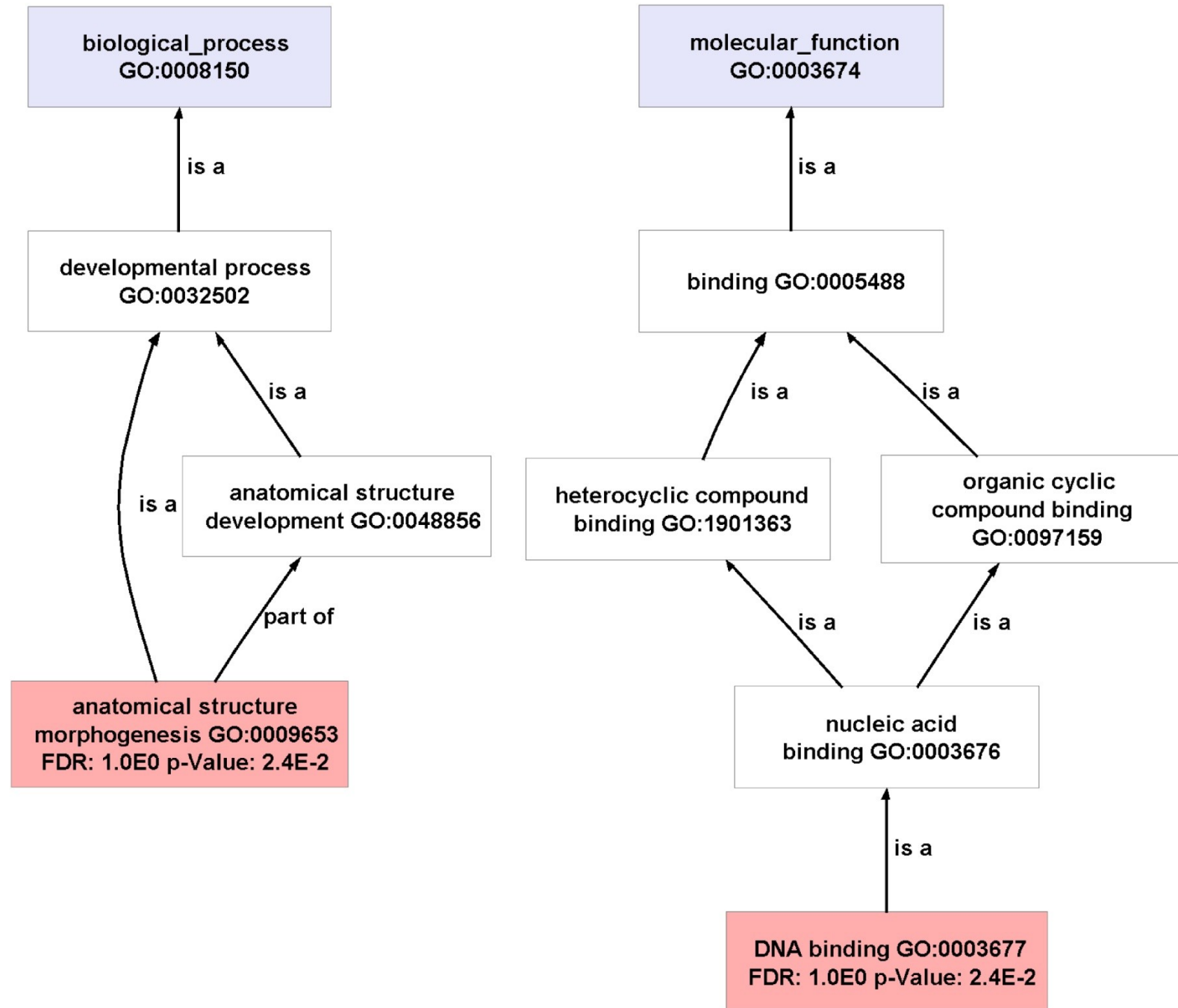

Supplement: Supplementary file 8 — Additional file 8: Figure S4: GO enrichment of phosphoproteins represented by the phosphorylation motifs in B. distachyon. GO enrichment graphs of phosphoproteins contained each of 11 motifs were displayed by Blast2GO software. No GO terms were significantly enriched from phosphoproteins containing motif 7 or motif 11. The statistical significance of the enrichment analysis is represented by a scale of red tones whose intensity is proportional to the degree of significance starting from p < 0.05. (PDF 2 MB) [file 12864_2014_6177_MOESM8_ESM.pdf]

Figure S5

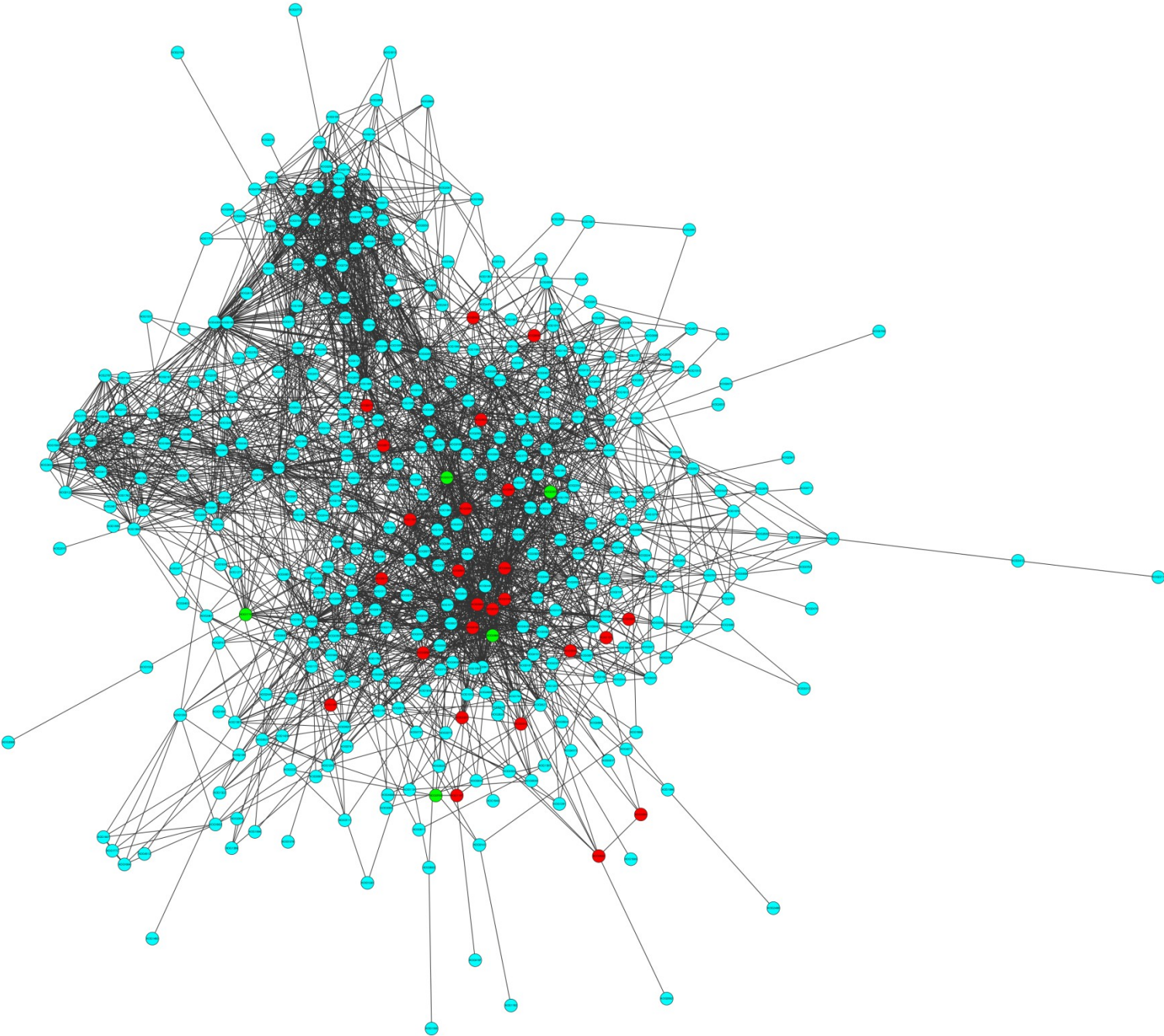

Supplement: Supplementary file 9 — Additional file 9: Figure S5: Protein-protein interaction network of the phosphoproteins. The confidence (score) was set as the highest (0.900) and only the phosphoproteins identified in this study were used to construct the interaction profiles. Protein kinases and protein phosphatases are highlighted in red and green respectively and other phosphoproteins identified in this study are shown with sky-blue nodes. (PDF 630 KB) [file 12864_2014_6177_MOESM9_ESM.pdf]

A

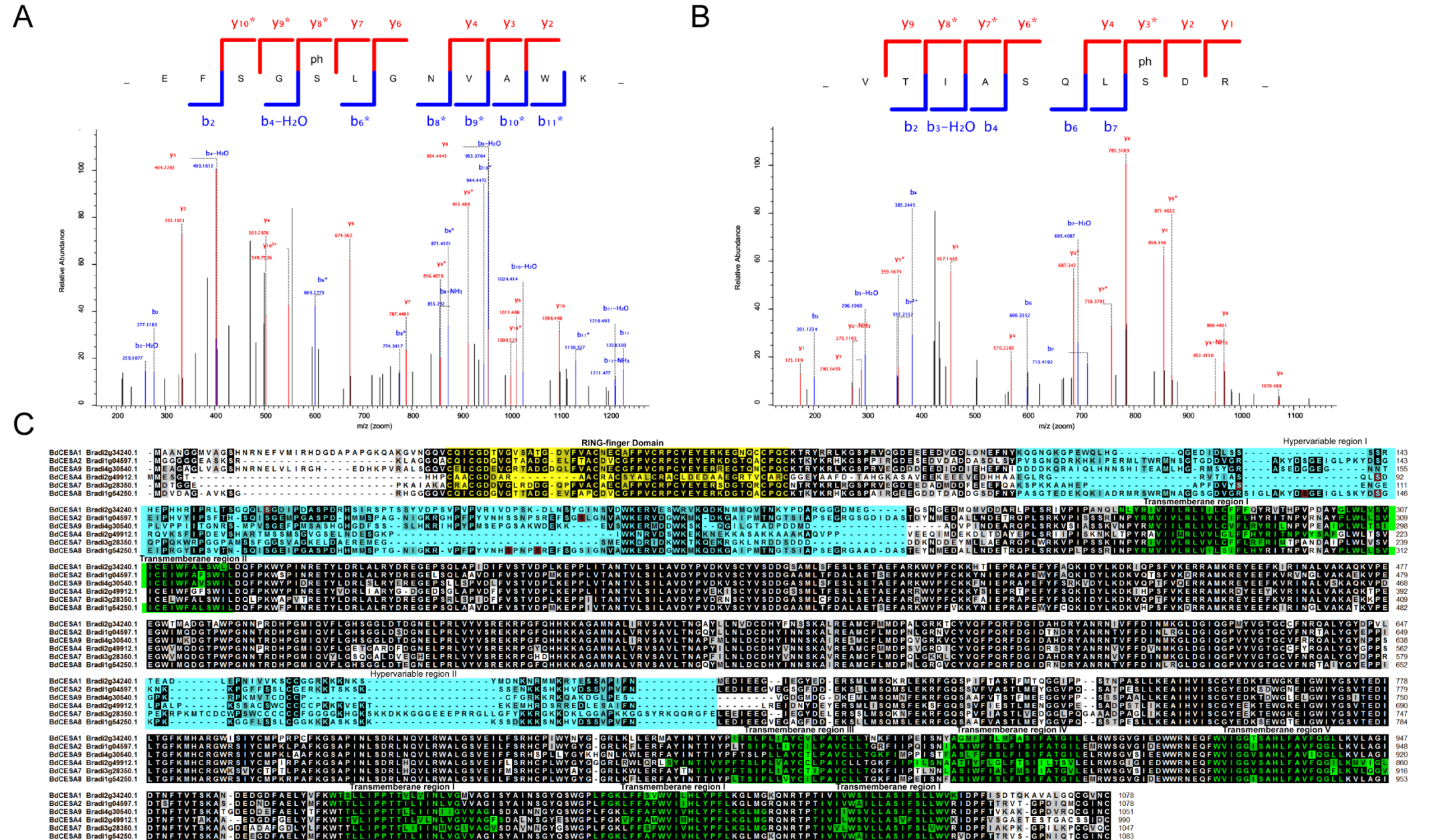

Supplement: Supplementary file 10 — Additional file 10: Figure S6: Phosphorylation sites of the six BdCESAs. (A) and (B) The tandem mass spectrometry spectra of the phosphopeptides EFSGS(ph)LGNVAWK from BdCESA2 (Bradi1g04597.1) and VTIASQLS(ph)DR from BdCESA4 (Bradi2g49912.1). (C) Sequence alignment among the six BdCESAs. Phosphorylation sites identified in this study are highlighted in red. The RING domain and transmembrane regions are highlighted in yellow and green, respectively. (PDF 1 MB) [file 12864_2014_6177_MOESM10_ESM.pdf]
