# Supplementary material for: Large-scale phosphoproteome analysis in seedling leaves of Brachypodium distachyon L
Source: BMC Genomics. 2014 May 16;15(1):375. doi: 10.1186/1471-2164-15-375 (PMC4079959; doi:10.1186/1471-2164-15-375)
Supplement: Supplementary file 6 — Additional file 6: Figure S3: Pathway mapping of identified phosphoproteins by KEGG. Phosphoproteins were analyzed in the KEGG B. distachyon pathway database. The phosphoproteins were used to search the KEGG B. distachyon database and mapped to B. distachyon specific pathways with KEGG Mapper. In each pathway map, objects with red foreground color and blue background color represent the highly conserved phosphoproteins; objects with pink foreground and blue background represent the other conserved phosphoproteins; objects with yellow foreground and blue background represent proteins identified as novel phosphoproteins; objects with green background represent the total proteins in the B. distachyon KEGG database. Details of the pathways can be found in Additional file 5: Table S3. (PDF 1 MB) [file 12864_2014_6177_MOESM6_ESM.pdf]

Figure S3
A

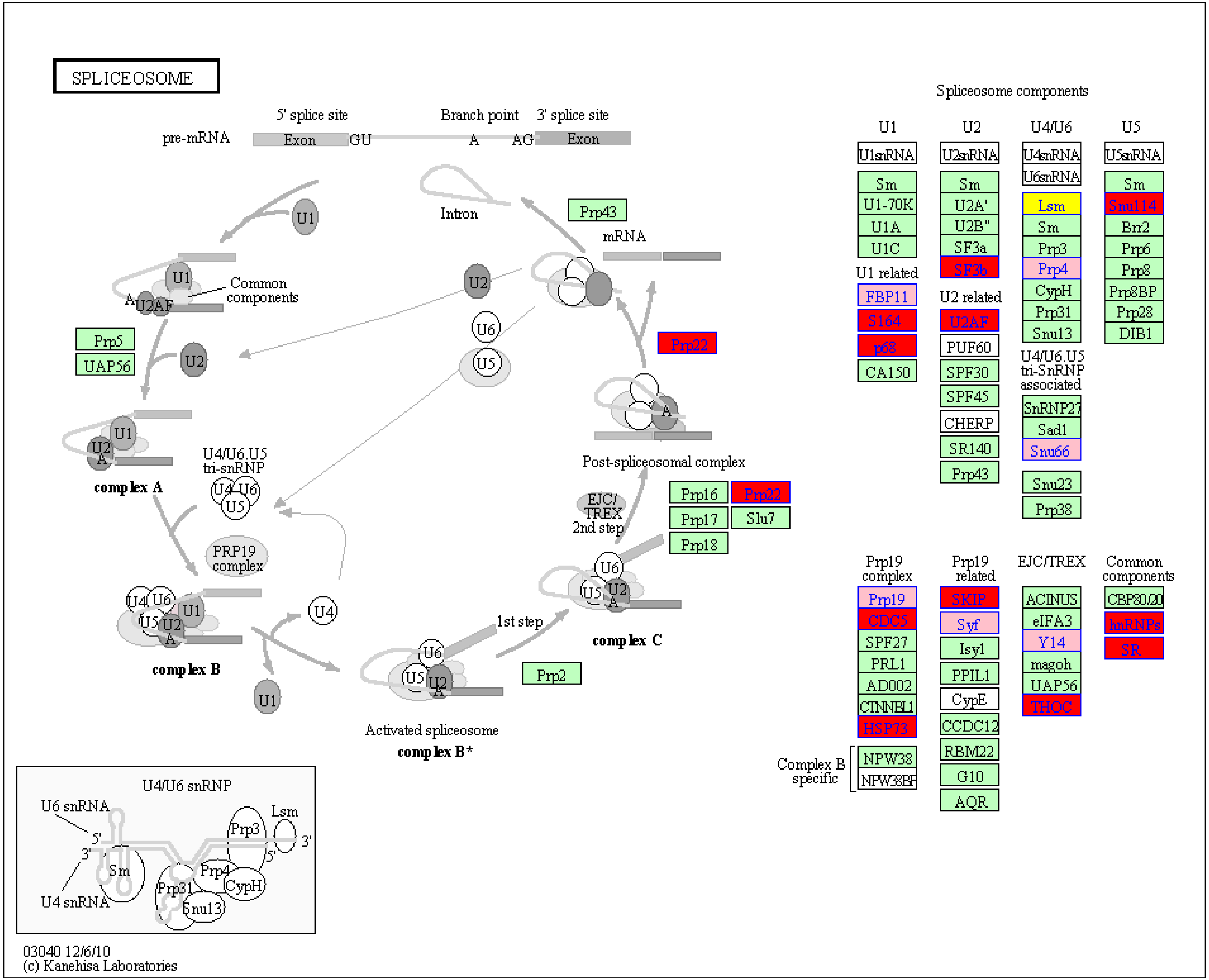
SPLICEOSOME
5' splice site
Branch point
3' splice site
pre-mRNA
Exon
GU
A
AG
Exon
Intron
mRNA
Prp43
U1
U2
U6
U5
Common components
U2AF
Prp5
UAP56
Prp22
Post-spliceosomal complex
complex A
U4/U6.U5 tri-snRNP
U4
EJC/ TREX
2nd step
Prp16
Prp22
Prp17
Slu7
Prp18
PRP19 complex
complex B
complex C
1st step
Prp2
Activated spliceosome
complex B*
U4/U6 snRNP
U6 snRNA
Lsm
Prp3
Sm
Prp4
CypH
Prp31
Snu13
U4 snRNA
Spliceosome components
U1
U2
U4/U6
U5
U1snRNA
U2snRNA
U4snRNA
U6snRNA
U5snRNA
Sm
U1-70K
U1A
U1C
U2A'
U2B"
SF3a
SF3b
Lsm
Prp3
Prp4
CypH
Prp31
Snu13
Snu114
Brr2
Prp6
Prp8
Prp8BP
Prp28
DIB1
U1 related
FBP11
S164
p68
CA150
U2 related
U2AF
PUF60
SPF30
SPF45
CHERP
SR140
Prp43
U4/U6.U5 tri-SnRNP associated
SnRNP27
Sad1
Snu66
Snu23
Prp38
Prp19 complex
Prp19
CDC5
SPF27
PRL1
AD002
CTNNBL1
HSP73
Prp19 related
SKIP
Syf
Isy1
PPIL1
CypE
CCDC12
RBM22
G10
AQR
EJC/TREX
ACINUS
eIFA3
Y14
magoh
UAP56
THOC
Common components
CBP80/20
hnRNPs
SR
Complex B specific
NPW38
NPW38BP
03040 12/6/10
(c) Kanehisa Laboratories

B

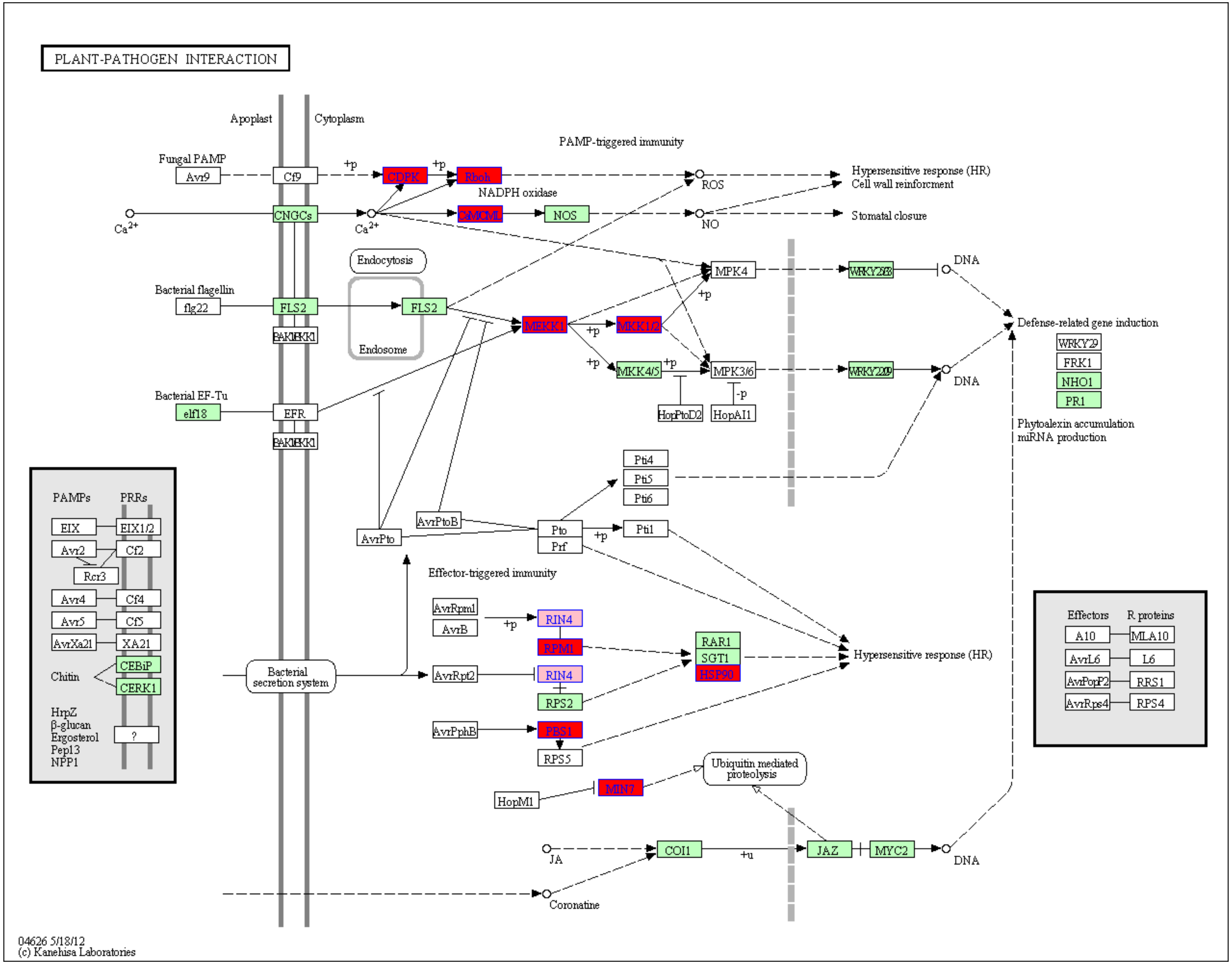

PLANT-PATHOGEN INTERACTION
Apoplast
Cytoplasm
PAMP-triggered immunity
Fungal PAMP
Avr9
Cf9
+p
CDPK
Rboh
NADPH oxidase
ROS
Hypersensitive response (HR)
Cell wall reinforcment
Ca2+
CNGCs
CaM/CML
NOS
NO
Stomatal closure
Endocytosis
Endosome
Bacterial flagellin
flg22
FLS2
BAK1/BKK1
MPK4
WRKY22/33
DNA
MEKK1
MKK1/2
MKK4/5
MPK3/6
WRKY22/29
-p
HopPtoD2
HopAI1
Defense-related gene induction
WRKY29
FRK1
NHO1
PR1
Phytoalexin accumulation
miRNA production
Bacterial EF-Tu
elf18
EFR
Pti4
Pti5
Pti6
AvrPtoB
AvrPto
Pto
Prf
Pti1
Effector-triggered immunity
PAMPs
PRRs
EIX
EIX1/2
Avr2
Cf2
Rcr3
Avr4
Cf4
Avr5
Cf5
AvrXa21
XA21
Chitin
CEBiP
CERK1
HrpZ
β-glucan
Ergosterol
Pep13
NPP1
?
AvrRpm1
AvrB
RIN4
RPM1
RAR1
SGT1
HSP90
Hypersensitive response (HR)
Bacterial secretion system
AvrRpt2
RPS2
AvrPphB
PBS1
RPS5
Effectors
R proteins
A10
MLA10
AvrL6
L6
AvrPopP2
RRS1
AvrRps4
RPS4
Ubiquitin mediated proteolysis
HopM1
MIN7
JA
COI1
+u
JAZ
MYC2
Coronatine
04626 5/18/12
(c) Kanehisa Laboratories

C

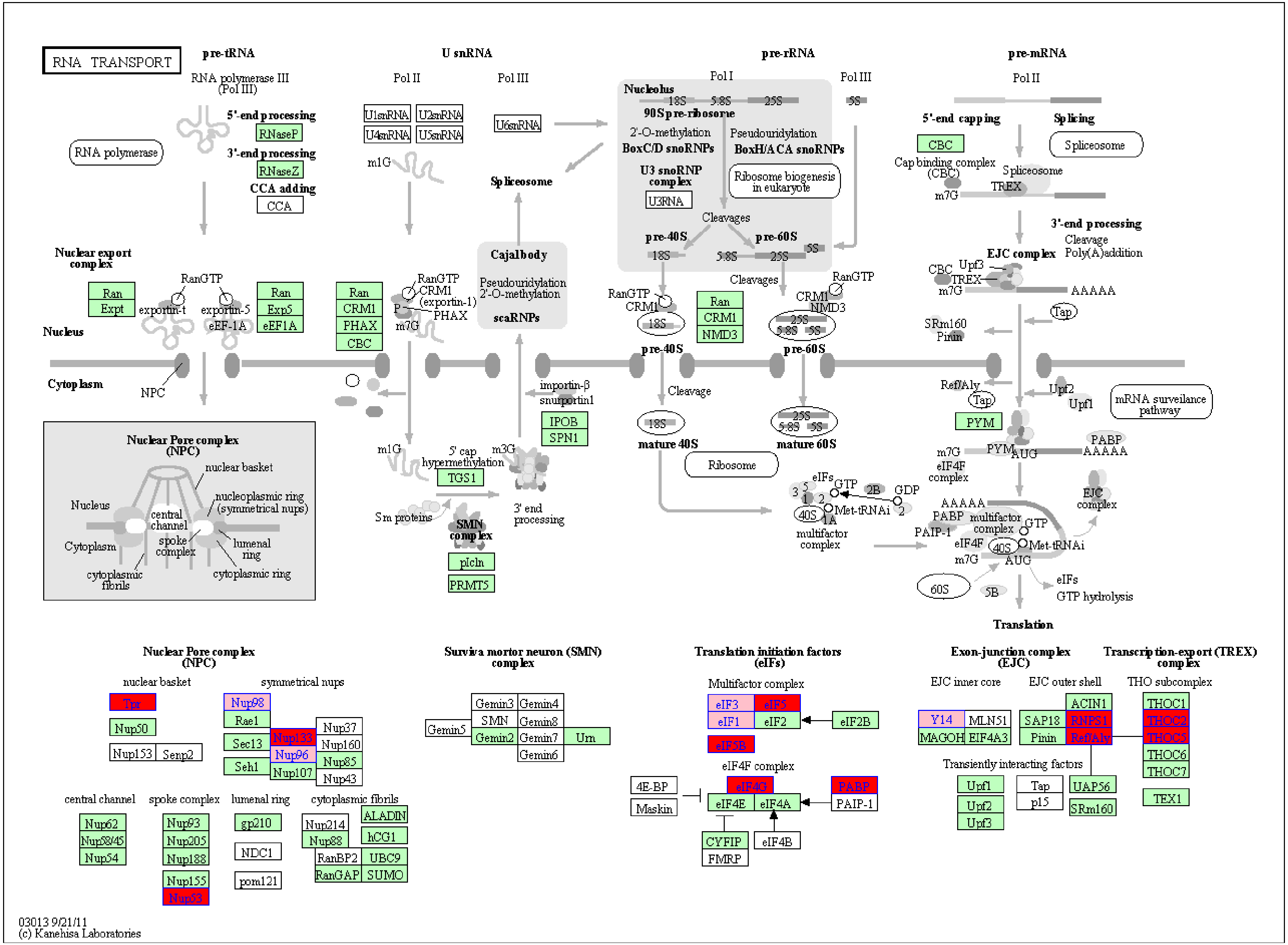

RNA TRANSPORT
pre-tRNA
U snRNA
pre-rRNA
pre-mRNA
Nucleus
Cytoplasm
Nuclear Pore complex (NPC)
Survival motor neuron (SMN) complex
Translation initiation factors (eIFs)
Exon-junction complex (EJC)
Transcription-export (TREX) complex
Translation
03013 9/21/11
(c) Kanehisa Laboratories

D

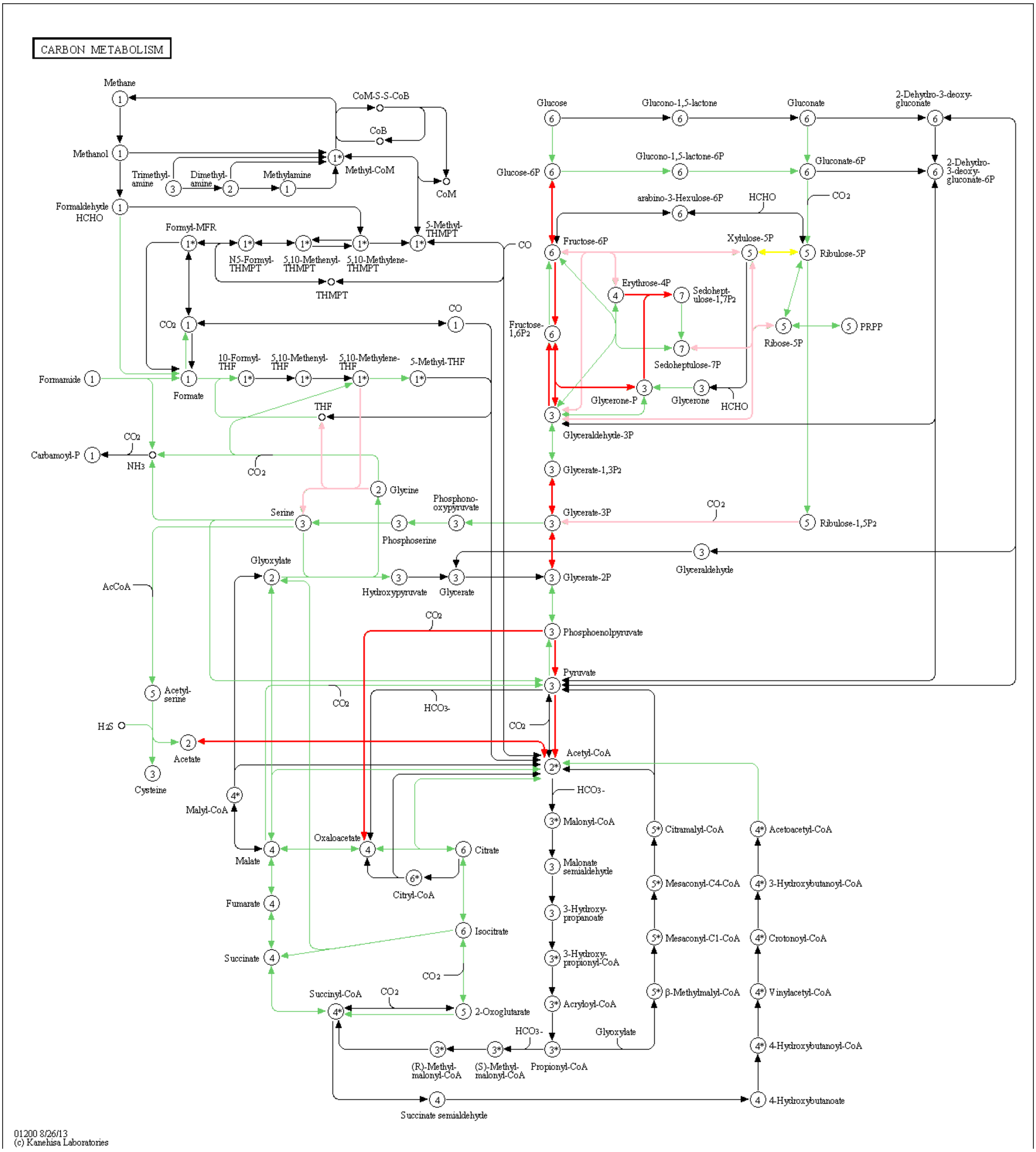
CARBON METABOLISM
Methane
Methanol
Formaldehyde
HCHO
Trimethyl-amine
Dimethyl-amine
Methylamine
CoM-S-S-CoB
CoB
Methyl-CoM
CoM
Formyl-MFR
N5-Formyl-THMPT
5,10-Methenyl-THMPT
5,10-Methylene-THMPT
5-Methyl-THMPT
THMPT
CO
CO2
Formamide
Formate
10-Formyl-THF
5,10-Methenyl-THF
5,10-Methylene-THF
5-Methyl-THF
THF
Carbamoyl-P
NH3
Glycine
Serine
Phosphono-oxypyruvate
Phosphoserine
Glyoxylate
Hydroxypyruvate
Glycerate
AcCoA
Acetyl-serine
H2S
Acetate
Cysteine
Malyl-CoA
Malate
Oxaloacetate
Citrate
Citryl-CoA
Fumarate
Isocitrate
Succinate
Succinyl-CoA
2-Oxoglutarate
(R)-Methyl-malonyl-CoA
(S)-Methyl-malonyl-CoA
Propionyl-CoA
Succinate semialdehyde
Glucose
Glucono-1,5-lactone
Gluconate
2-Dehydro-3-deoxy-gluconate
Glucose-6P
Glucono-1,5-lactone-6P
Gluconate-6P
2-Dehydro-3-deoxy-gluconate-6P
arabino-3-Hexulose-6P
HCHO
Fructose-6P
Xylulose-5P
Ribulose-5P
Erythrose-4P
Sedohept-ulose-1,7P2
Fructose-1,6P2
Ribose-5P
PRPP
Sedoheptulose-7P
Glycerone-P
Glycerone
Glyceraldehyde-3P
Glycerate-1,3P2
Glycerate-3P
Ribulose-1,5P2
Glyceraldehyde
Glycerate-2P
Phosphoenolpyruvate
Pyruvate
HCO3-
Acetyl-CoA
Malonyl-CoA
Malonate semialdehyde
3-Hydroxy-propanoate
3-Hydroxy-propionyl-CoA
Acryloyl-CoA
Citramalyl-CoA
Mesaconyl-C4-CoA
Mesaconyl-C1-CoA
β-Methylmalyl-CoA
Acetoacetyl-CoA
3-Hydroxybutanoyl-CoA
Crotonoyl-CoA
Vinylacetyl-CoA
4-Hydroxybutanoyl-CoA
4-Hydroxybutanoate
01200 8/26/13
(c) Kanehisa Laboratories

E

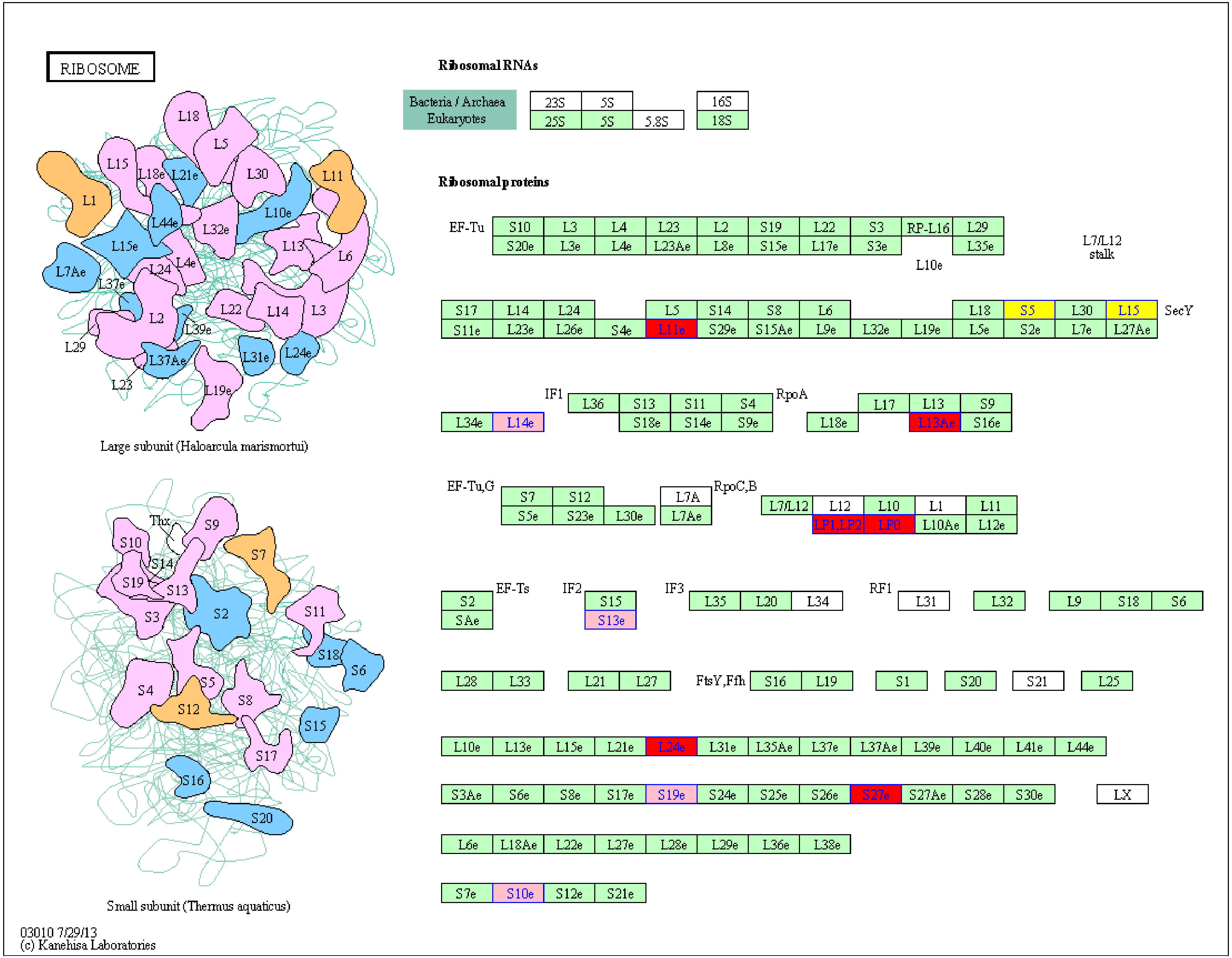
RIBOSOME
L18
L5
L15
L18e
L21e
L30
L11
L1
L44e
L10e
L32e
L15e
L13
L6
L4e
L7Ae
L24
L37e
L22
L14
L3
L2
L39e
L29
L37Ae
L31e
L24e
L23
L19e
Large subunit (Haloarcula marismortui)
Thx
S9
S10
S7
S14
S19
S13
S3
S2
S11
S18
S6
S4
S5
S8
S12
S15
S17
S16
S20
Small subunit (Thermus aquaticus)
03010 7/29/13
(c) Kanehisa Laboratories
Ribosomal RNAs
Bacteria / Archaea
Eukaryotes
23S
5S
16S
25S
5S
5.8S
18S
Ribosomal proteins
EF-Tu
S10
L3
L4
L23
L2
S19
L22
S3
RP-L16
L29
S20e
L3e
L4e
L23Ae
L8e
S15e
L17e
S3e
L35e
L10e
L7/L12
stalk
S17
L14
L24
L5
S14
S8
L6
L18
S5
L30
L15
SecY
S11e
L23e
L26e
S4e
L11e
S29e
S15Ae
L9e
L32e
L19e
L5e
S2e
L7e
L27Ae
IF1
L36
S13
S11
S4
RpoA
L17
L13
S9
L34e
L14e
S18e
S14e
S9e
L18e
L13Ae
S16e
EF-Tu,G
S7
S12
L7A
RpoC,B
L7/L12
L12
L10
L1
L11
S5e
S23e
L30e
L7Ae
LP1,LP2
LP0
L10Ae
L12e
S2
EF-Ts
IF2
S15
IF3
L35
L20
L34
RF1
L31
L32
L9
S18
S6
SAe
S13e
L28
L33
L21
L27
FtsY,Ffh
S16
L19
S1
S20
S21
L25
L10e
L13e
L15e
L21e
L24e
L31e
L35Ae
L37e
L37Ae
L39e
L40e
L41e
L44e
S3Ae
S6e
S8e
S17e
S19e
S24e
S25e
S26e
S27e
S27Ae
S28e
S30e
LX
L6e
L18Ae
L22e
L27e
L28e
L29e
L36e
L38e
S7e
S10e
S12e
S21e

F

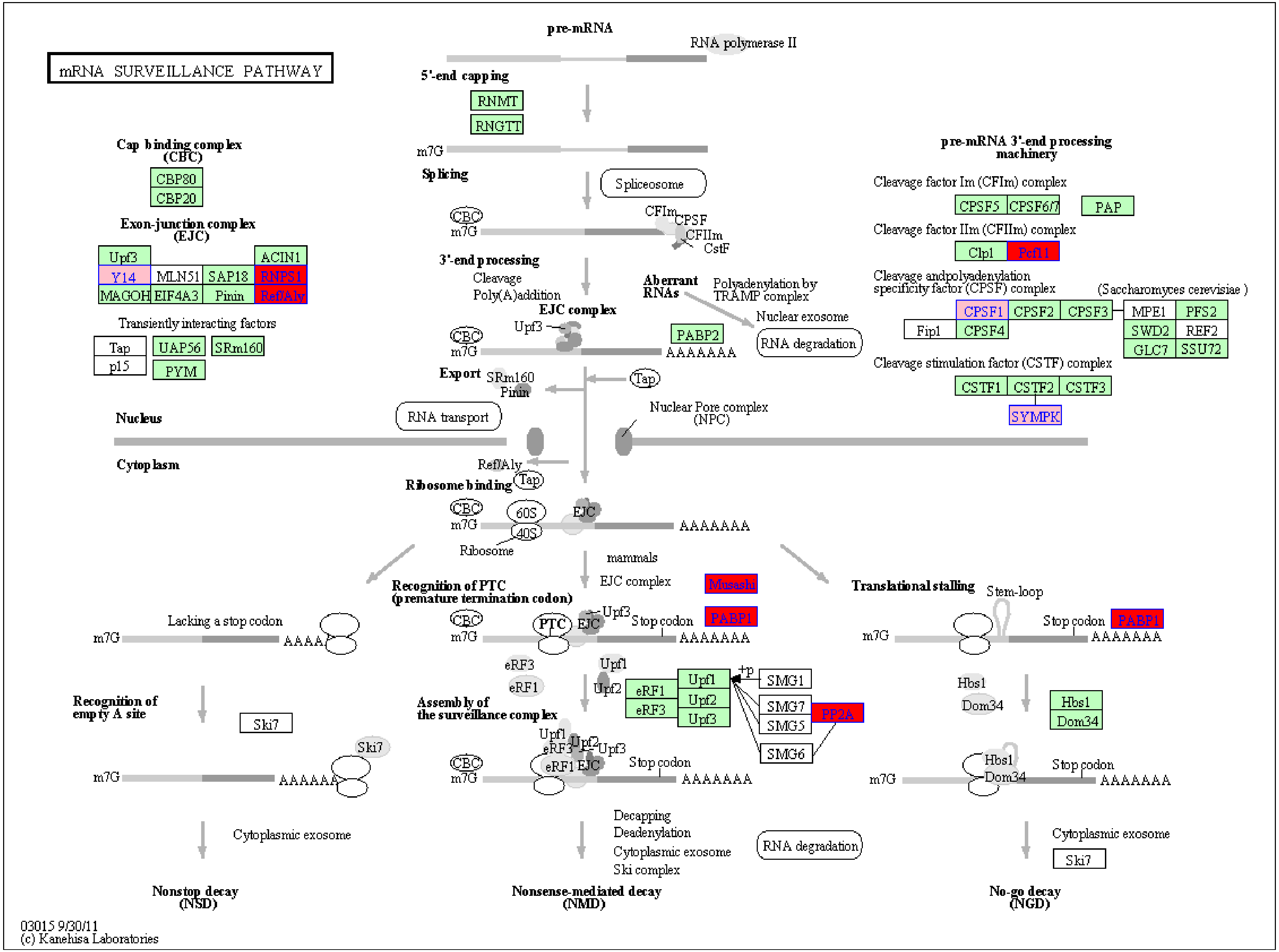
mRNA SURVEILLANCE PATHWAY
pre-mRNA
RNA polymerase II
5'-end capping
RNMT
RNGTT
m7G
Splicing
Spliceosome
CBC
CFIm
CPSF
CFIIm
CstF
3'-end processing
Cleavage
Poly(A)addition
Aberrant RNAs
Polyadenylation by TRAMP complex
Nuclear exosome
RNA degradation
EJC complex
Upf3
PABP2
AAAAAAA
Export
SRm160
Pinin
Tap
Nuclear Pore complex (NPC)
RNA transport
Cap binding complex (CBC)
CBP80
CBP20
Exon-junction complex (EJC)
Upf3
ACIN1
Y14
MLN51
SAP18
RNPS1
MAGOH
EIF4A3
Pinin
Ref/Aly
Transiently interacting factors
Tap
p15
UAP56
SRm160
PYM
pre-mRNA 3'-end processing machinery
Cleavage factor Im (CFIm) complex
CPSF5
CPSF6/7
PAP
Cleavage factor IIm (CFIIm) complex
Clp1
Pcf11
Cleavage and polyadenylation specificity factor (CPSF) complex
(Saccharomyces cerevisiae)
CPSF1
CPSF2
CPSF3
MPE1
PFS2
Fip1
CPSF4
SWD2
REF2
GLC7
SSU72
Cleavage stimulation factor (CSTF) complex
CSTF1
CSTF2
CSTF3
SYMPK
Nucleus
Cytoplasm
Ref/Aly
Ribosome binding
60S
40S
EJC
Ribosome
mammals
EJC complex
Musashi
PABP1
Recognition of PTC (premature termination codon)
PTC
Stop codon
Lacking a stop codon
AAAA
Translational stalling
Stem-loop
eRF3
eRF1
Upf1
Upf2
eRF1
eRF3
Upf1
Upf2
Upf3
+p
SMG1
SMG7
SMG5
PP2A
SMG6
Assembly of the surveillance complex
Recognition of empty A site
Ski7
Hbs1
Dom34
Decapping
Deadenylation
Cytoplasmic exosome
Ski complex
Cytoplasmic exosome
Ski7
Nonstop decay (NSD)
Nonsense-mediated decay (NMD)
No-go decay (NGD)
03015 9/30/11
(c) Kanehisa Laboratories

G

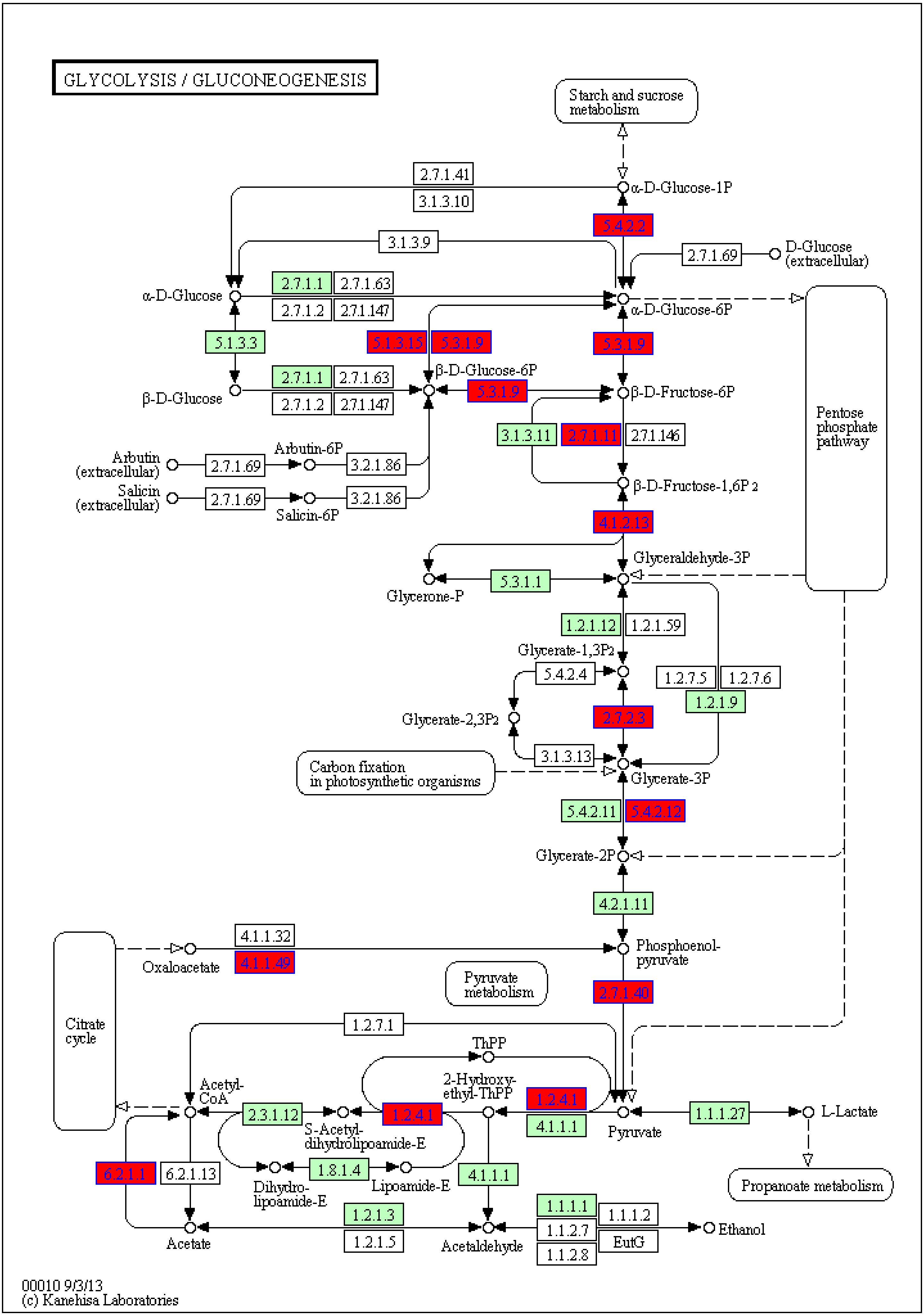

GLYCOLYSIS / GLUCONEOGENESIS
Starch and sucrose metabolism
2.7.1.41
3.1.3.10
α-D-Glucose-1P
5.4.2.2
3.1.3.9
2.7.1.69
D-Glucose (extracellular)
2.7.1.1
2.7.1.63
2.7.1.2
2.7.1.147
α-D-Glucose
α-D-Glucose-6P
5.1.3.3
5.1.3.15
5.3.1.9
β-D-Glucose-6P
β-D-Glucose
β-D-Fructose-6P
Pentose phosphate pathway
3.1.3.11
2.7.1.11
2.7.1.146
Arbutin (extracellular)
Arbutin-6P
3.2.1.86
Salicin (extracellular)
Salicin-6P
β-D-Fructose-1,6P2
4.1.2.13
Glyceraldehyde-3P
5.3.1.1
Glycerone-P
1.2.1.12
1.2.1.59
Glycerate-1,3P2
5.4.2.4
1.2.7.5
1.2.7.6
1.2.1.9
Glycerate-2,3P2
2.7.2.3
3.1.3.13
Carbon fixation in photosynthetic organisms
Glycerate-3P
5.4.2.11
5.4.2.12
Glycerate-2P
4.2.1.11
4.1.1.32
4.1.1.49
Oxaloacetate
Phosphoenol-pyruvate
Citrate cycle
Pyruvate metabolism
2.7.1.40
1.2.7.1
ThPP
2-Hydroxy-ethyl-ThPP
Acetyl-CoA
2.3.1.12
S-Acetyl-dihydrolipoamide-E
1.2.4.1
4.1.1.1
Pyruvate
1.1.1.27
L-Lactate
Propanoate metabolism
6.2.1.1
6.2.1.13
Dihydro-lipoamide-E
1.8.1.4
Lipoamide-E
1.2.1.3
1.2.1.5
1.1.1.1
1.1.2.7
1.1.2.8
1.1.1.2
EutG
Ethanol
Acetate
Acetaldehyde
00010 9/3/13
(c) Kanehisa Laboratories

H

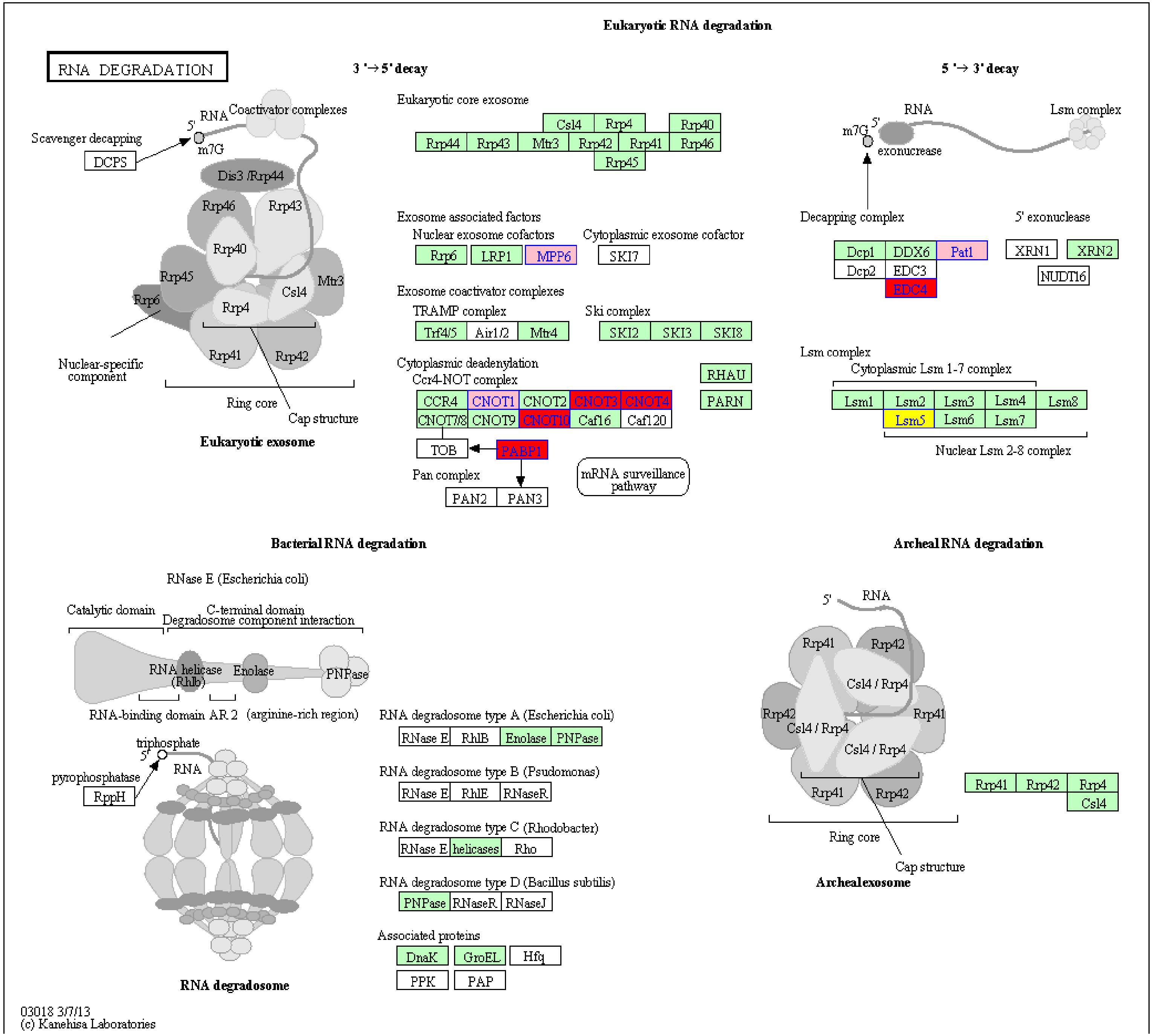
Eukaryotic RNA degradation
RNA DEGRADATION
3 '→ 5' decay
5 '→ 3' decay
Eukaryotic core exosome
Csl4 Rrp4 Rrp40
Rrp44 Rrp43 Mtr3 Rrp42 Rrp41 Rrp46
Rrp45
Scavenger decapping
DCPS
RNA
Coactivator complexes
m7G
Dis3 /Rrp44
Rrp46 Rrp43 Rrp40 Rrp45 Mtr3 Csl4 Rrp6 Rrp4 Rrp41 Rrp42
Nuclear-specific component
Ring core
Cap structure
Eukaryotic exosome
Exosome associated factors
Nuclear exosome cofactors
Cytoplasmic exosome cofactor
Rrp6 LRP1 MPP6 SKI7
Exosome coactivator complexes
TRAMP complex
Trf4/5 Air1/2 Mtr4
Ski complex
SKI2 SKI3 SKI8
Cytoplasmic deadenylation
Ccr4-NOT complex
CCR4 CNOT1 CNOT2 CNOT3 CNOT4
CNOT7/8 CNOT9 CNOT10 Caf16 Caf120
RHAU
PARN
TOB
PABP1
Pan complex
PAN2 PAN3
mRNA surveillance pathway
RNA
Lsm complex
m7G
exonucrease
Decapping complex
5' exonuclease
Dcp1 DDX6 Pat1
Dcp2 EDC3
EDC4
XRN1 XRN2
NUDT16
Lsm complex
Cytoplasmic Lsm 1-7 complex
Lsm1 Lsm2 Lsm3 Lsm4 Lsm8
Lsm5 Lsm6 Lsm7
Nuclear Lsm 2-8 complex
Bacterial RNA degradation
RNase E (Escherichia coli)
Catalytic domain
C-terminal domain
Degradosome component interaction
RNA helicase (Rhlb)
Enolase
PNPase
RNA-binding domain AR 2 (arginine-rich region)
triphosphate
RNA
pyrophosphatase
RppH
RNA degradosome
RNA degradosome type A (Escherichia coli)
RNase E RhlB Enolase PNPase
RNA degradosome type B (Psudomonas)
RNase E RhlE RNaseR
RNA degradosome type C (Rhodobacter)
RNase E helicases Rho
RNA degradosome type D (Bacillus subtilis)
PNPase RNaseR RNaseJ
Associated proteins
DnaK GroEL Hfq
PPK PAP
Archeal RNA degradation
RNA
Rrp41 Rrp42
Csl4 / Rrp4
Rrp42 Rrp41
Csl4 / Rrp4
Csl4 / Rrp4
Rrp41 Rrp42
Ring core
Cap structure
Archeal exosome
Rrp41 Rrp42 Rrp4
Csl4
03018 3/7/13
(c) Kanehisa Laboratories

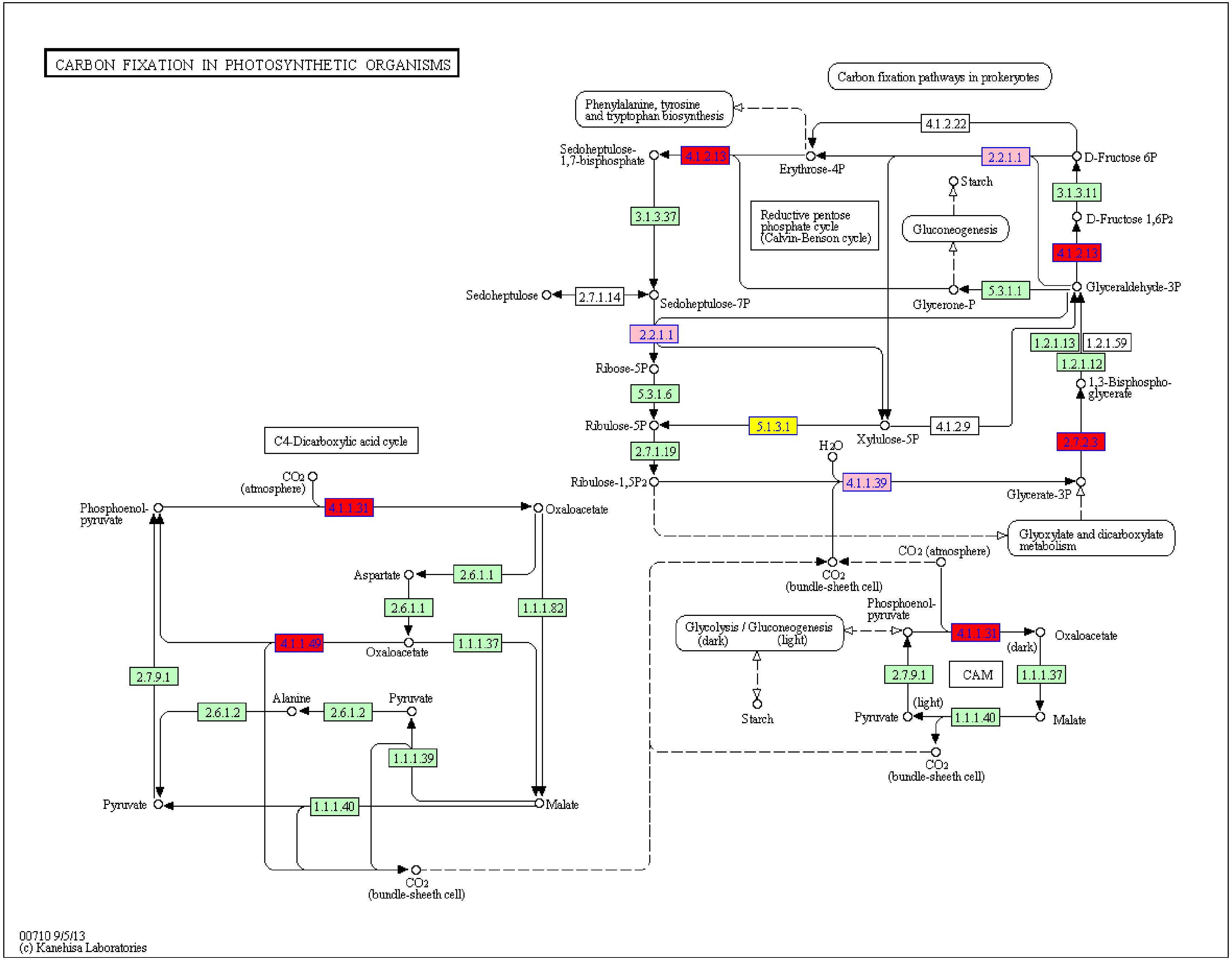
CARBON FIXATION IN PHOTOSYNTHETIC ORGANISMS
Carbon fixation pathways in prokaryotes
Phenylalanine, tyrosine and tryptophan biosynthesis
4.1.2.22
Sedoheptulose-1,7-bisphosphate
4.1.2.13
Erythrose-4P
2.2.1.1
D-Fructose 6P
Starch
3.1.3.11
3.1.3.37
Reductive pentose phosphate cycle (Calvin-Benson cycle)
Gluconeogenesis
D-Fructose 1,6P2
4.1.2.13
Sedoheptulose
2.7.1.14
Sedoheptulose-7P
Glycerone-P
5.3.1.1
Glyceraldehyde-3P
2.2.1.1
1.2.1.13
1.2.1.59
1.2.1.12
Ribose-5P
1,3-Bisphospho-glycerate
5.3.1.6
Ribulose-5P
5.1.3.1
Xylulose-5P
4.1.2.9
2.7.2.3
C4-Dicarboxylic acid cycle
2.7.1.19
H_2O
CO_2 (atmosphere)
Ribulose-1,5P2
4.1.1.39
Glycerate-3P
Phosphoenol-pyruvate
4.1.1.31
Oxaloacetate
Glyoxylate and dicarboxylate metabolism
CO_2 (atmosphere)
CO_2 (bundle-sheeth cell)
Aspartate
2.6.1.1
2.6.1.1
1.1.1.82
Phosphoenol-pyruvate
Glycolysis / Gluconeogenesis
(dark) (light)
4.1.1.31
Oxaloacetate
(dark)
4.1.1.49
Oxaloacetate
1.1.1.37
2.7.9.1
2.7.9.1
CAM
1.1.1.37
(light)
Alanine
Pyruvate
2.6.1.2
2.6.1.2
Starch
Pyruvate
1.1.1.40
Malate
1.1.1.39
CO_2 (bundle-sheeth cell)
Pyruvate
1.1.1.40
Malate
CO_2 (bundle-sheeth cell)
00710 9/5/13
(c) Kanehisa Laboratories

J

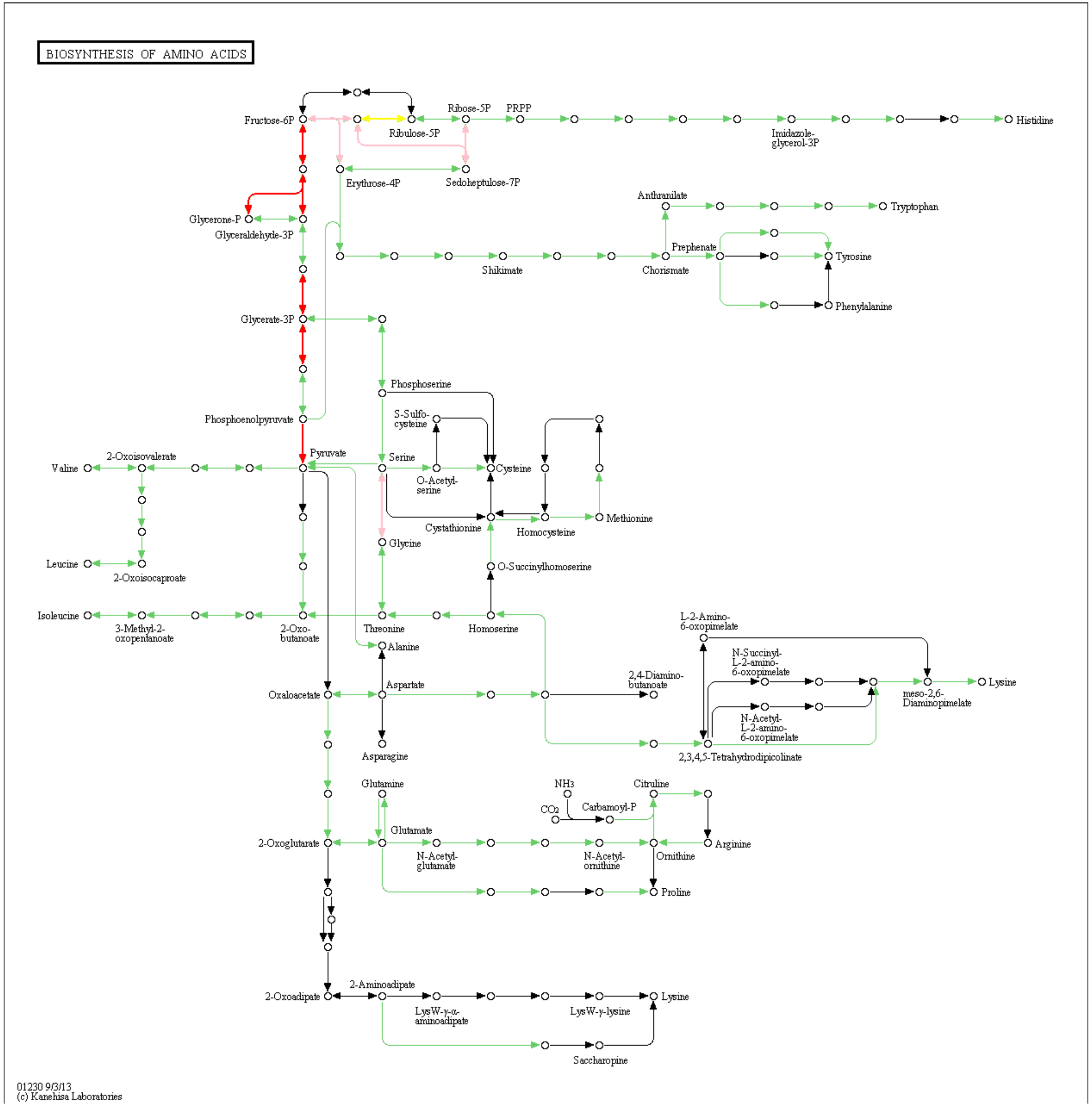
BIOSYNTHESIS OF AMINO ACIDS
Fructose-6P
Ribose-5P
PRPP
Ribulose-5P
Imidazole-glycerol-3P
Histidine
Erythrose-4P
Sedoheptulose-7P
Anthranilate
Tryptophan
Glycerone-P
Glyceraldehyde-3P
Prephenate
Tyrosine
Shikimate
Chorismate
Phenylalanine
Glycerate-3P
Phosphoserine
S-Sulfo-cysteine
Phosphoenolpyruvate
2-Oxoisovalerate
Pyruvate
Serine
Valine
O-Acetyl-serine
Cysteine
Cystathionine
Homocysteine
Methionine
Glycine
Leucine
2-Oxoisocaproate
O-Succinylhomoserine
Isoleucine
3-Methyl-2-oxopentanoate
2-Oxo-butanoate
Threonine
Homoserine
L-2-Amino-6-oxopimelate
Alanine
N-Succinyl-L-2-amino-6-oxopimelate
2,4-Diamino-butanoate
Aspartate
Oxaloacetate
Lysine
meso-2,6-Diaminopimelate
N-Acetyl-L-2-amino-6-oxopimelate
Asparagine
2,3,4,5-Tetrahydrodipicolinate
Glutamine
NH3
Citruline
CO2
Carbamoyl-P
Glutamate
2-Oxoglutarate
Arginine
N-Acetyl-glutamate
N-Acetyl-ornithine
Ornithine
Proline
2-Aminoadipate
2-Oxoadipate
Lysine
LysW-γ-α-aminoadipate
LysW-γ-lysine
Saccharopine
01230 9/3/13
(c) Kanehisa Laboratories

K

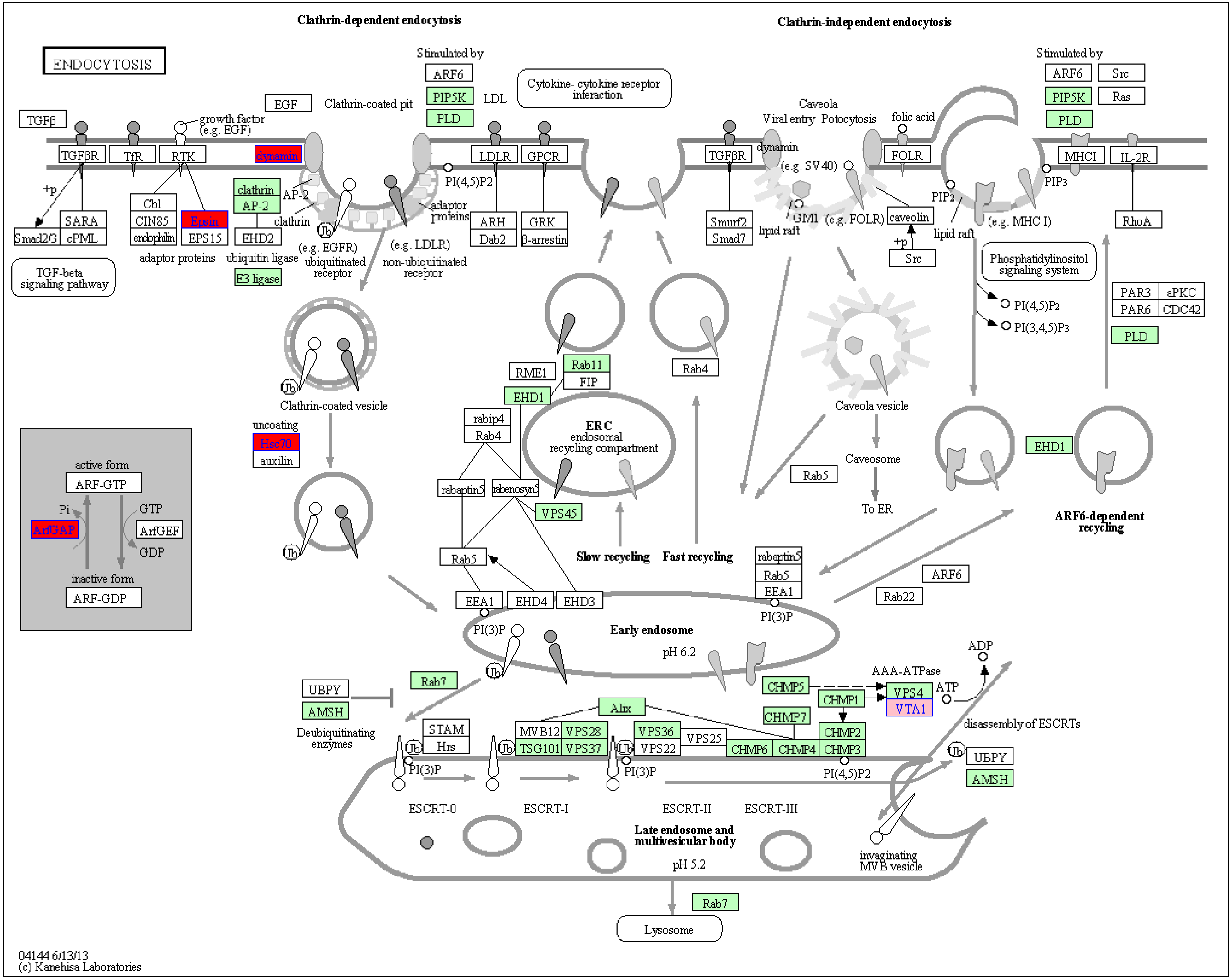
Clathrin-dependent endocytosis
Clathrin-independent endocytosis
ENDOCYTOSIS
Stimulated by
ARF6
PIP5K
PLD
LDL
Cytokine- cytokine receptor interaction
EGF
Clathrin-coated pit
TGFβ
growth factor (e.g. EGF)
TGFβR
TfR
RTK
dynamin
+p
Smad2/3
SARA
cPML
Cbl
CIN85
endophilin
Epsin
EPS15
clathrin
AP-2
EHD2
adaptor proteins
ubiquitin ligase
E3 ligase
TGF-beta signaling pathway
(e.g. EGFR) ubiquitinated receptor
(e.g. LDLR) non-ubiquitinated receptor
PI(4,5)P2
adaptor proteins
LDLR
GPCR
ARH
Dab2
GRK
β-arrestin
Caveola
Viral entry
Potocytosis
folic acid
(e.g. SV40)
Smurf2
Smad7
lipid raft
GM1
(e.g. FOLR)
caveolin
FOLR
+p
Src
PIP2
lipid raft
(e.g. MHC I)
PIP3
MHCI
IL-2R
RhoA
Ras
Phosphatidylinositol signaling system
PI(4,5)P2
PI(3,4,5)P3
PAR3
aPKC
PAR6
CDC42
PLD
Clathrin-coated vesicle
uncoating
Hsc70
auxilin
RME1
Rab11
FIP
EHD1
rabip4
Rab4
ERC endosomal recycling compartment
rabaptin5
rabenosyn5
VPS45
Rab5
Slow recycling
Fast recycling
Caveola vesicle
Caveosome
Rab5
To ER
EHD1
ARF6-dependent recycling
active form
ARF-GTP
Pi
ArfGAP
GTP
ArfGEF
GDP
inactive form
ARF-GDP
EEA1
EHD4
EHD3
rabaptin5
Rab5
EEA1
ARF6
Rab22
PI(3)P
PI(3)P
Early endosome
pH 6.2
Ub
ADP
AAA-ATPase
ATP
UBPY
AMSH
Deubiquitinating enzymes
Rab7
STAM
Hrs
MVB12
VPS28
TSG101
VPS37
Alix
VPS36
VPS22
VPS25
CHMP5
CHMP1
VPS4
VTA1
CHMP7
CHMP2
CHMP6
CHMP4
CHMP3
disassembly of ESCRTs
UBPY
AMSH
PI(3)P
PI(3)P
PI(4,5)P2
ESCRT-0
ESCRT-I
ESCRT-II
ESCRT-III
Late endosome and multivesicular body
pH 5.2
invaginating MVB vesicle
Rab7
Lysosome
04144 6/13/13
(c) Kanehisa Laboratories

L

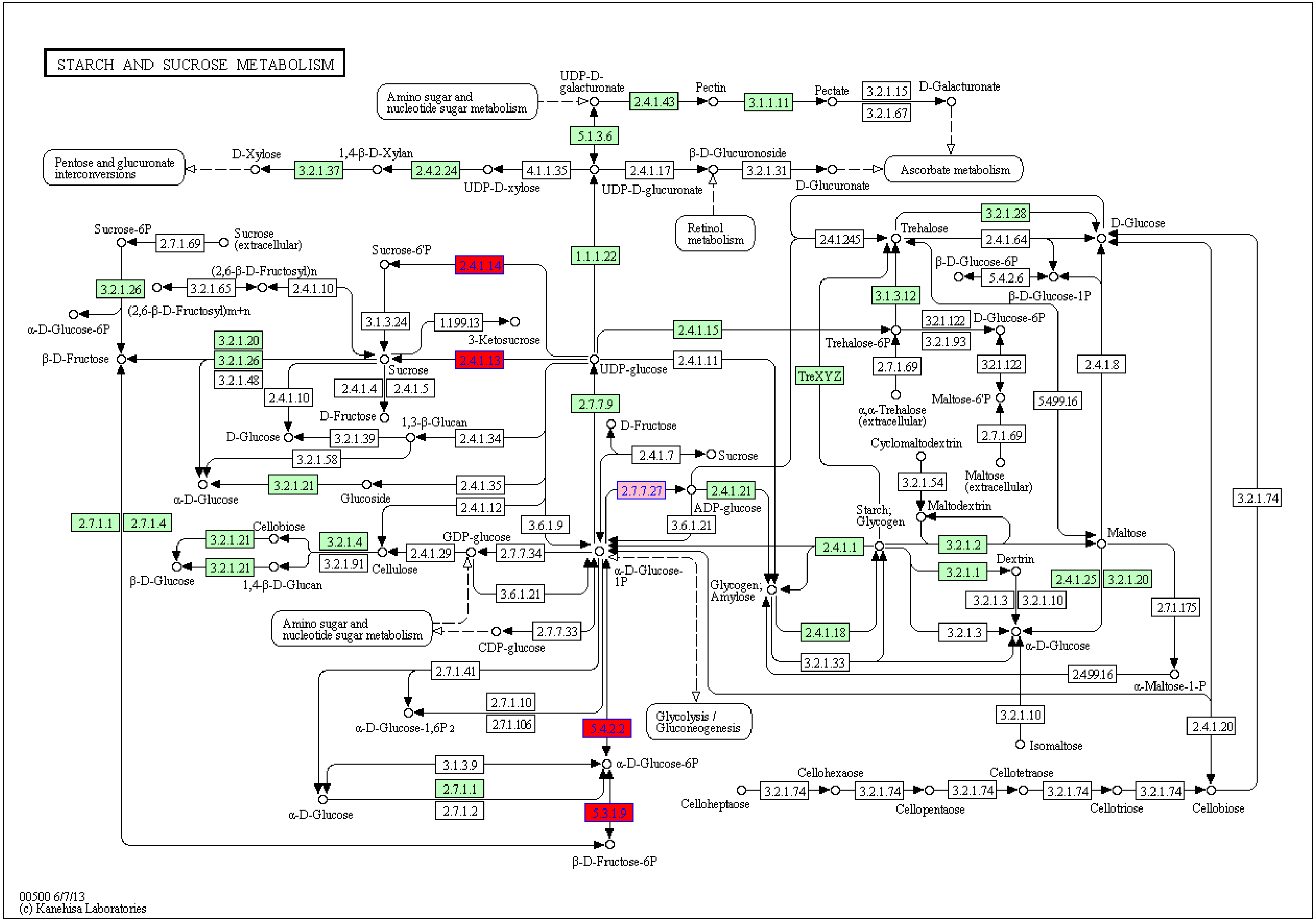

STARCH AND SUCROSE METABOLISM
Amino sugar and nucleotide sugar metabolism
UDP-D-galacturonate
2.4.1.43
Pectin
3.1.1.11
Pectate
3.2.1.15
3.2.1.67
D-Galacturonate
5.1.3.6
Pentose and glucuronate interconversions
D-Xylose
3.2.1.37
1,4-β-D-Xylan
2.4.2.24
4.1.1.35
UDP-D-xylose
2.4.1.17
β-D-Glucuronoside
3.2.1.31
D-Glucuronate
Ascorbate metabolism
UDP-D-glucuronate
Retinol metabolism
Sucrose-6P
2.7.1.69
Sucrose (extracellular)
Sucrose-6'P
2.4.1.14
1.1.1.22
(2,6-β-D-Fructosyl)n
3.2.1.26
3.2.1.65
2.4.1.10
(2,6-β-D-Fructosyl)m+n
α-D-Glucose-6P
3.1.3.24
1.1.99.13
3-Ketosucrose
3.2.1.20
3.2.1.26
3.2.1.48
β-D-Fructose
2.4.1.13
Sucrose
2.4.1.4
2.4.1.5
2.4.1.10
D-Fructose
D-Glucose
3.2.1.39
1,3-β-Glucan
2.4.1.34
3.2.1.58
α-D-Glucose
3.2.1.21
Glucoside
2.4.1.35
2.4.1.12
3.6.1.9
2.7.1.1
2.7.1.4
Cellobiose
3.2.1.21
3.2.1.4
β-D-Glucose
3.2.1.21
1,4-β-D-Glucan
3.2.1.91
Cellulose
GDP-glucose
2.4.1.29
2.7.7.34
3.6.1.21
Amino sugar and nucleotide sugar metabolism
2.7.7.33
CDP-glucose
UDP-glucose
2.4.1.15
2.4.1.11
2.7.7.9
D-Fructose
2.4.1.7
Sucrose
2.7.7.27
2.4.1.21
ADP-glucose
3.6.1.21
α-D-Glucose-1P
2.7.1.41
2.7.1.10
α-D-Glucose-1,6P2
2.7.1.106
5.4.2.2
Glycolysis / Gluconeogenesis
3.1.3.9
α-D-Glucose-6P
2.7.1.1
2.7.1.2
α-D-Glucose
5.3.1.9
β-D-Fructose-6P
3.2.1.28
D-Glucose
2.4.1.245
Trehalose
2.4.1.64
β-D-Glucose-6P
5.4.2.6
β-D-Glucose-1P
3.1.3.12
3.2.1.122
D-Glucose-6P
3.2.1.93
Trehalose-6P
TreXYZ
2.7.1.69
3.2.1.122
2.4.1.8
α,α-Trehalose (extracellular)
Maltose-6'P
5.4.99.16
2.7.1.69
Cyclomaltodextrin
Maltose (extracellular)
3.2.1.54
Maltodextrin
Starch; Glycogen
3.2.1.74
2.4.1.1
3.2.1.2
Maltose
3.2.1.1
Dextrin
2.4.1.25
3.2.1.20
3.2.1.3
3.2.1.10
Glycogen; Amylose
2.4.1.18
3.2.1.3
α-D-Glucose
2.7.1.175
3.2.1.33
2.4.99.16
α-Maltose-1-P
3.2.1.10
Isomaltose
2.4.1.20
Cellohexaose
Celloheptaose
3.2.1.74
3.2.1.74
Cellopentaose
Cellotetraose
3.2.1.74
3.2.1.74
Cellotriose
3.2.1.74
Cellobiose
00500 6/7/13
(c) Kanehisa Laboratories

M

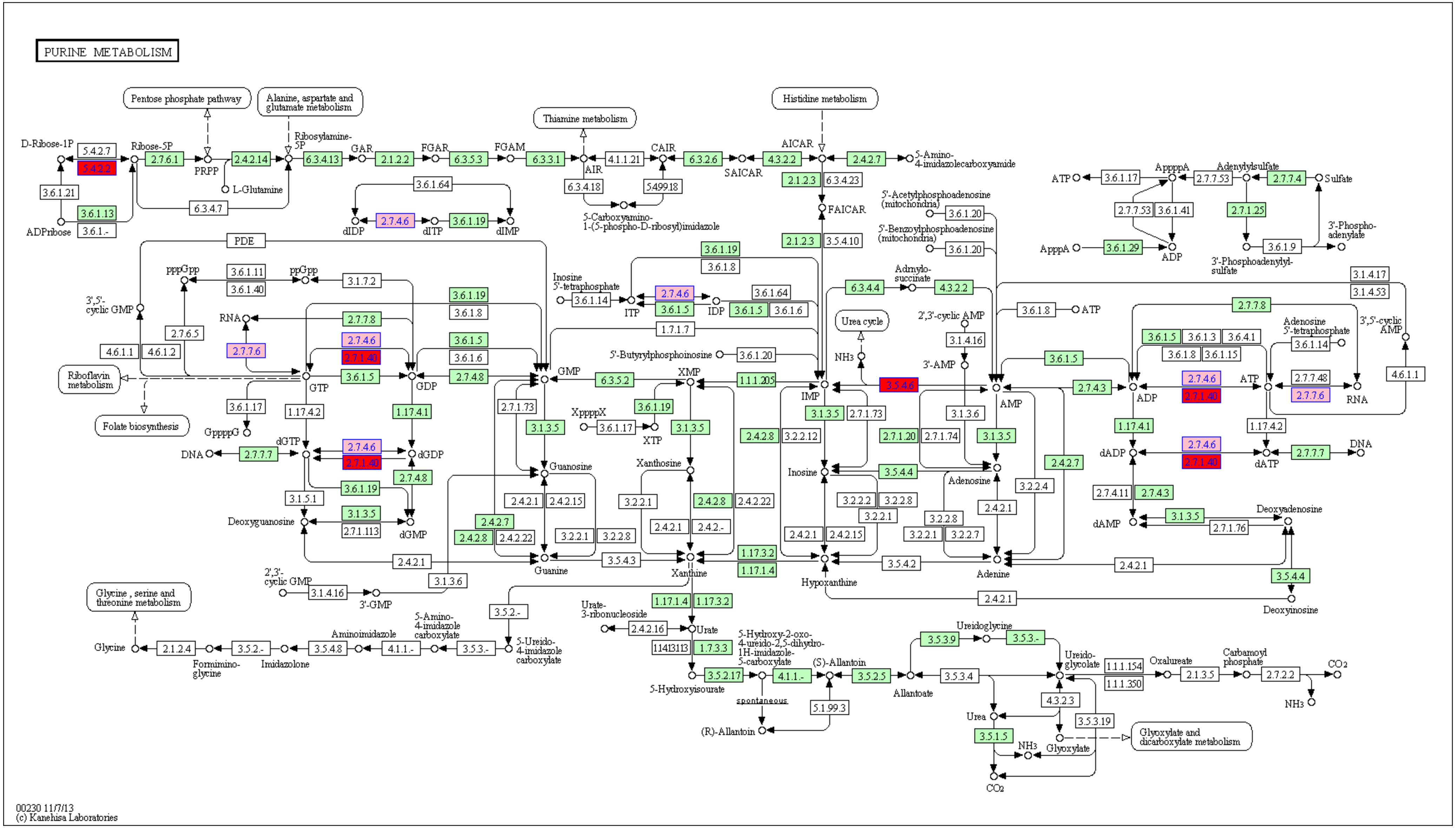
PURINE METABOLISM
Pentose phosphate pathway
Alanine, aspartate and glutamate metabolism
Thiamine metabolism
Histidine metabolism
D-Ribose-1P
5.4.2.7
5.4.2.2
Ribose-5P
2.7.6.1
PRPP
2.4.2.14
Ribosylamine-5P
6.3.4.13
GAR
2.1.2.2
FGAR
6.3.5.3
FGAM
6.3.3.1
AIR
4.1.1.21
CAIR
6.3.2.6
SAICAR
4.3.2.2
AICAR
2.4.2.7
5-Amino-4-imidazolecarboxyamide
L-Glutamine
6.3.4.7
3.6.1.21
3.6.1.13
3.6.1.-
ADPribose
3.6.1.64
2.7.4.6
3.6.1.19
dIDP
dITP
dIMP
6.3.4.18
5.4.99.18
5-Carboxyamino-1-(5-phospho-D-ribosyl)imidazole
2.1.2.3
6.3.4.23
FAICAR
3.5.4.10
5'-Acetylphosphoadenosine (mitochondria)
5'-Benzoylphosphoadenosine (mitochondria)
3.6.1.20
ATP
3.6.1.17
ApppA
2.7.7.53
Adenylylsulfate
2.7.7.4
Sulfate
3.6.1.41
2.7.1.25
3'-Phospho-adenylate
3.6.1.9
ApppA
3.6.1.29
ADP
3'-Phosphoadenylyl-sulfate
PDE
pppGpp
3.6.1.11
3.6.1.40
ppGpp
3.1.7.2
3',5'-cyclic GMP
2.7.6.5
RNA
2.7.7.8
4.6.1.1
4.6.1.2
2.7.7.6
2.7.1.40
3.6.1.5
3.6.1.6
2.7.4.8
Riboflavin metabolism
GTP
GDP
GMP
Folate biosynthesis
3.6.1.17
GppppG
1.17.4.2
1.17.4.1
dGTP
dGDP
DNA
2.7.7.7
3.1.5.1
Deoxyguanosine
2.7.1.113
dGMP
Inosine 5'-tetraphosphate
3.6.1.14
ITP
3.6.1.8
IDP
3.6.1.64
1.7.1.7
5'-Butyrylphosphoinosine
6.3.5.2
XMP
1.1.1.205
IMP
3.5.4.6
AMP
6.3.4.4
Adenylo-succinate
4.3.2.2
Urea cycle
NH3
2',3'-cyclic AMP
3.1.4.16
3'-AMP
3.1.3.6
XppppX
3.1.3.5
XTP
2.7.1.73
Guanosine
Xanthosine
Inosine
Adenosine
3.5.4.4
2.4.2.8
3.2.2.12
2.7.1.20
2.7.1.74
2.4.2.7
3.2.2.4
2.4.2.1
2.4.2.15
3.2.2.1
3.2.2.8
2.4.2.-
3.2.2.2
3.2.2.7
Guanine
3.5.4.3
Xanthine
1.17.3.2
1.17.1.4
Hypoxanthine
3.5.4.2
Adenine
3',5'-cyclic AMP
3.1.4.17
3.1.4.53
2.7.7.8
Adenosine 5'-tetraphosphate
3.6.1.3
3.6.4.1
3.6.1.15
2.7.4.3
2.7.7.48
dADP
dATP
2.7.4.11
dAMP
2.7.1.76
Deoxyadenosine
Deoxyinosine
2',3'-cyclic GMP
3'-GMP
3.1.3.6
Glycine, serine and threonine metabolism
Glycine
2.1.2.4
Formimino-glycine
3.5.2.-
Imidazolone
3.5.4.8
Aminoimidazole
4.1.1.-
5-Amino-4-imidazole carboxylate
3.5.3.-
5-Ureido-4-imidazole carboxylate
Urate-3-ribonucleoside
2.4.2.16
Urate
1.14.13.113
1.7.3.3
5-Hydroxy-2-oxo-4-ureido-2,5-dihydro-1H-imidazole-5-carboxylate
5-Hydroxyisourate
3.5.2.17
spontaneous
(S)-Allantoin
3.5.2.5
5.1.99.3
(R)-Allantoin
Allantoate
3.5.3.4
Ureidoglycine
3.5.3.9
Ureido-glycolate
4.3.2.3
3.5.3.19
Urea
3.5.1.5
NH3
Glyoxylate
Glyoxylate and dicarboxylate metabolism
CO2
1.1.1.154
1.1.1.350
Oxalureate
2.1.3.5
Carbamoyl phosphate
2.7.2.2
00230 11/7/13
(c) Kanehisa Laboratories

N

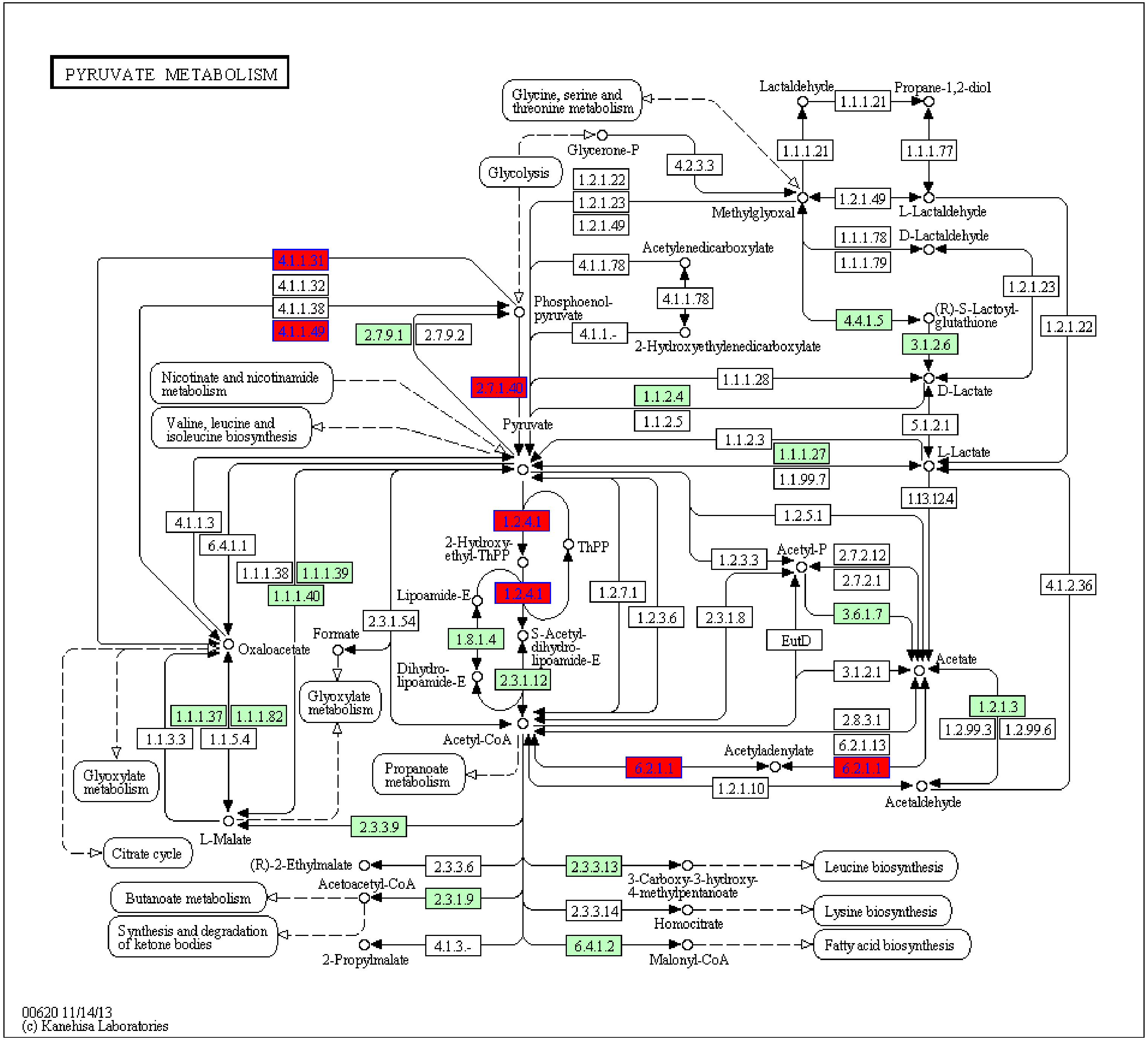
PYRUVATE METABOLISM
Glycine, serine and threonine metabolism
Lactaldehyde
Propane-1,2-diol
1.1.1.21
1.1.1.77
Glycerone-P
Glycolysis
4.2.3.3
1.2.1.22
1.2.1.23
1.2.1.49
Methylglyoxal
L-Lactaldehyde
1.1.1.78
1.1.1.79
D-Lactaldehyde
Acetylenedicarboxylate
4.1.1.31
4.1.1.32
4.1.1.38
4.1.1.49
4.1.1.78
Phosphoenol-pyruvate
4.4.1.5
(R)-S-Lactoyl-glutathione
2.7.9.1
2.7.9.2
4.1.1.-
2-Hydroxyethylenedicarboxylate
3.1.2.6
Nicotinate and nicotinamide metabolism
2.7.1.40
1.1.1.28
1.1.2.4
D-Lactate
Valine, leucine and isoleucine biosynthesis
Pyruvate
1.1.2.5
5.1.2.1
1.1.2.3
1.1.1.27
L-Lactate
1.1.99.7
1.13.12.4
4.1.1.3
6.4.1.1
1.2.4.1
2-Hydroxy-ethyl-ThPP
ThPP
1.2.5.1
1.2.3.3
Acetyl-P
2.7.2.12
2.7.2.1
1.1.1.38
1.1.1.39
1.1.1.40
Lipoamide-E
1.2.7.1
1.2.3.6
2.3.1.8
3.6.1.7
4.1.2.36
2.3.1.54
Formate
1.8.1.4
S-Acetyl-dihydro-lipoamide-E
EutD
Oxaloacetate
Acetate
Dihydro-lipoamide-E
2.3.1.12
3.1.2.1
Glyoxylate metabolism
1.2.1.3
1.1.1.37
1.1.1.82
2.8.3.1
1.2.99.3
1.2.99.6
1.1.3.3
1.1.5.4
Acetyl-CoA
6.2.1.13
Acetyladenylate
Propanoate metabolism
6.2.1.1
1.2.1.10
Acetaldehyde
2.3.3.9
L-Malate
Citrate cycle
(R)-2-Ethylmalate
2.3.3.6
2.3.3.13
Leucine biosynthesis
3-Carboxy-3-hydroxy-4-methylpentanoate
Acetoacetyl-CoA
Butanoate metabolism
2.3.1.9
2.3.3.14
Lysine biosynthesis
Homocitrate
Synthesis and degradation of ketone bodies
4.1.3.-
6.4.1.2
Fatty acid biosynthesis
2-Propylmalate
Malonyl-CoA
00620 11/14/13
(c) Kanehisa Laboratories

O

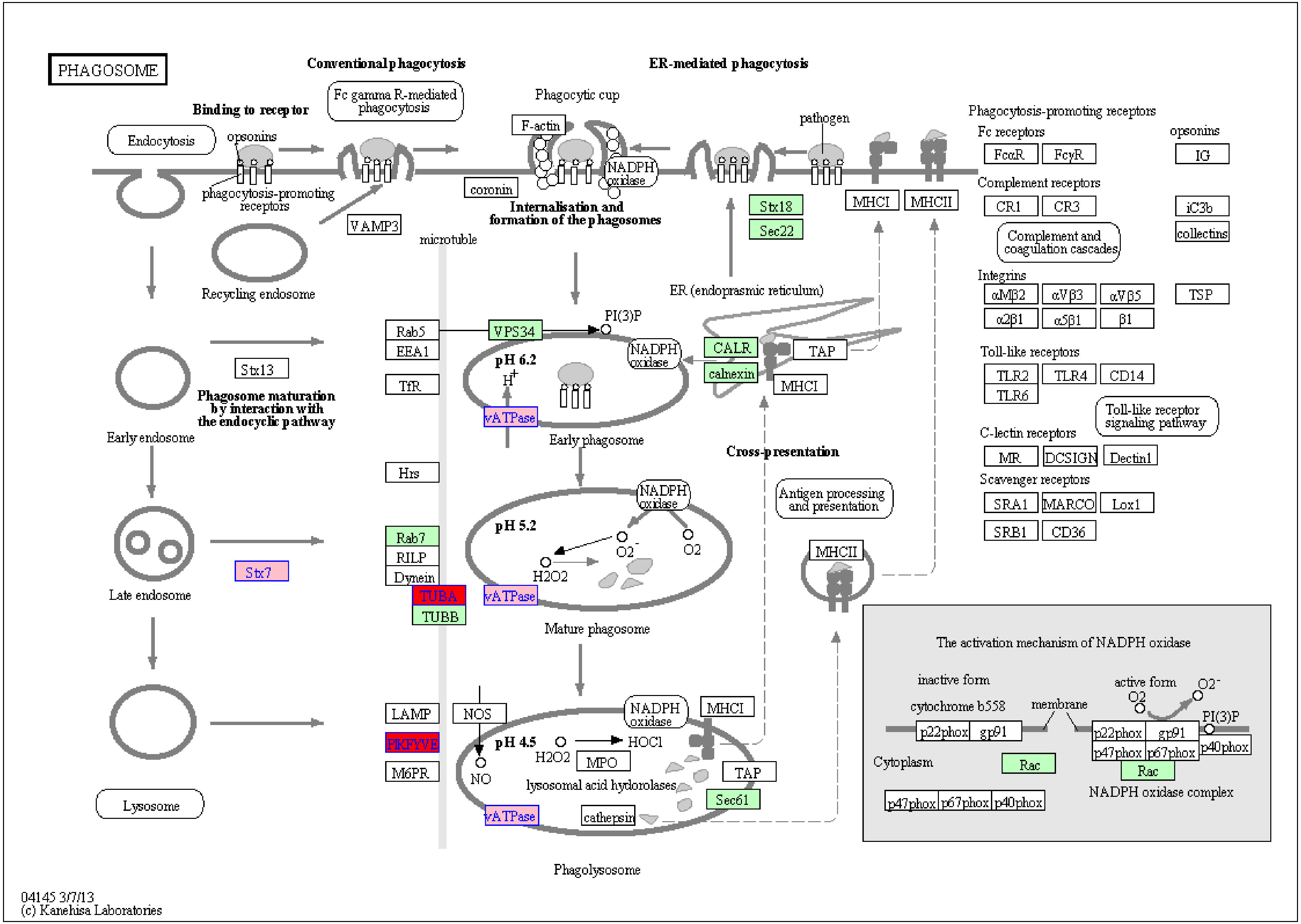

PHAGOSOME
Conventional phagocytosis
ER-mediated phagocytosis
Fc gamma R-mediated phagocytosis
Binding to receptor
Phagocytic cup
F-actin
pathogen
Endocytosis
opsonins
NADPH oxidase
phagocytosis-promoting receptors
coronin
VAMP3
Internalisation and formation of the phagosomes
Stx18
Sec22
MHCI
MHCII
microtuble
Recycling endosome
ER (endoplasmic reticulum)
Phagocytosis-promoting receptors
Fc receptors
FcαR
FcγR
opsonins
IG
Complement receptors
CR1
CR3
iC3b
collectins
Complement and coagulation cascades
Integrins
αMβ2
αVβ3
αVβ5
TSP
α2β1
α5β1
β1
Rab5
EEA1
VPS34
PI(3)P
CALR
calnexin
TAP
Stx13
TfR
pH 6.2
H+
vATPase
Early phagosome
Phagosome maturation by interaction with the endocyclic pathway
Early endosome
Toll-like receptors
TLR2
TLR4
CD14
TLR6
Toll-like receptor signaling pathway
C-lectin receptors
MR
DCSIGN
Dectin1
Scavenger receptors
SRA1
MARCO
Lox1
SRB1
CD36
Cross-presentation
Hrs
Antigen processing and presentation
pH 5.2
O2-
O2
H2O2
Rab7
RILP
Dynein
TUBA
TUBB
Stx7
Late endosome
Mature phagosome
The activation mechanism of NADPH oxidase
inactive form
active form
cytochrome b558
membrane
p22phox
gp91
p47phox
p67phox
p40phox
Cytoplasm
Rac
NADPH oxidase complex
LAMP
NOS
PIKFYVE
M6PR
pH 4.5
HOCl
NO
MPO
lysosomal acid hydrolases
cathepsin
Sec61
Lysosome
Phagolysosome
04145 3/7/13
(c) Kanehisa Laboratories

P

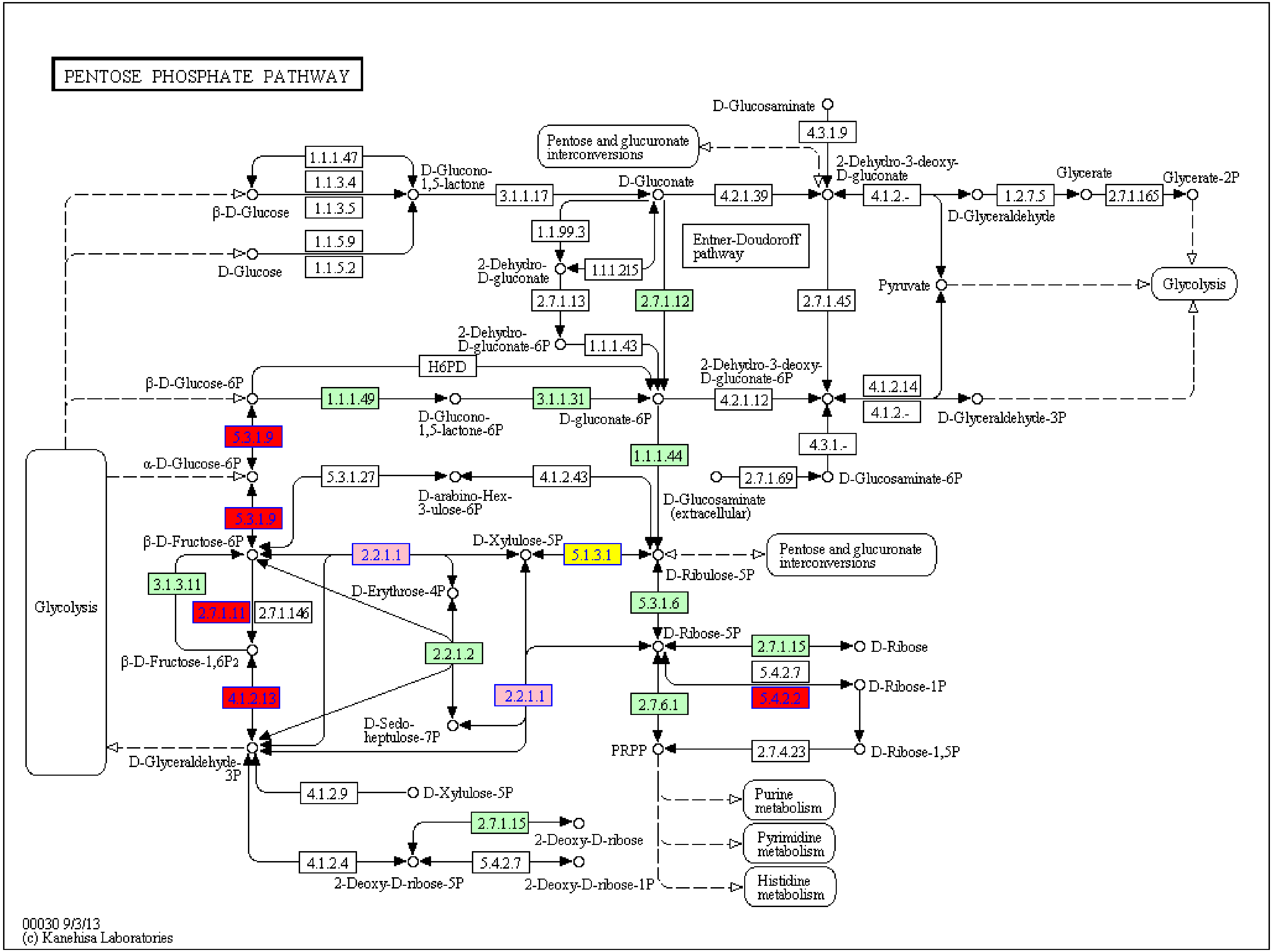
PENTOSE PHOSPHATE PATHWAY
D-Glucosaminate
4.3.1.9
Pentose and glucuronate interconversions
2-Dehydro-3-deoxy-D-gluconate
Glycerate
Glycerate-2P
1.1.1.47
1.1.3.4
1.1.3.5
1.1.5.9
1.1.5.2
D-Glucono-1,5-lactone
3.1.1.17
D-Gluconate
4.2.1.39
4.1.2.-
1.2.7.5
2.7.1.165
D-Glyceraldehyde
β-D-Glucose
D-Glucose
1.1.99.3
1.1.1.215
2-Dehydro-D-gluconate
Entner-Doudoroff pathway
2.7.1.13
2.7.1.12
2.7.1.45
Pyruvate
Glycolysis
2-Dehydro-D-gluconate-6P
1.1.1.43
H6PD
β-D-Glucose-6P
1.1.1.49
3.1.1.31
2-Dehydro-3-deoxy-D-gluconate-6P
4.2.1.12
4.1.2.14
4.1.2.-
D-Glyceraldehyde-3P
D-Glucono-1,5-lactone-6P
D-gluconate-6P
5.3.1.9
4.3.1.-
α-D-Glucose-6P
5.3.1.27
4.1.2.43
1.1.1.44
2.7.1.69
D-Glucosaminate-6P
D-arabino-Hex-3-ulose-6P
D-Glucosaminate (extracellular)
5.3.1.9
β-D-Fructose-6P
2.2.1.1
D-Xylulose-5P
5.1.3.1
Pentose and glucuronate interconversions
D-Ribulose-5P
3.1.3.11
D-Erythrose-4P
5.3.1.6
Glycolysis
2.7.1.11
2.7.1.146
D-Ribose-5P
2.7.1.15
D-Ribose
β-D-Fructose-1,6P2
2.2.1.2
5.4.2.7
D-Ribose-1P
4.1.2.13
2.2.1.1
5.4.2.2
2.7.6.1
D-Sedo-heptulose-7P
PRPP
2.7.4.23
D-Ribose-1,5P
D-Glyceraldehyde-3P
4.1.2.9
D-Xylulose-5P
Purine metabolism
2.7.1.15
2-Deoxy-D-ribose
Pyrimidine metabolism
4.1.2.4
5.4.2.7
2-Deoxy-D-ribose-5P
2-Deoxy-D-ribose-1P
Histidine metabolism
00030 9/3/13
(c) Kanehisa Laboratories

Q

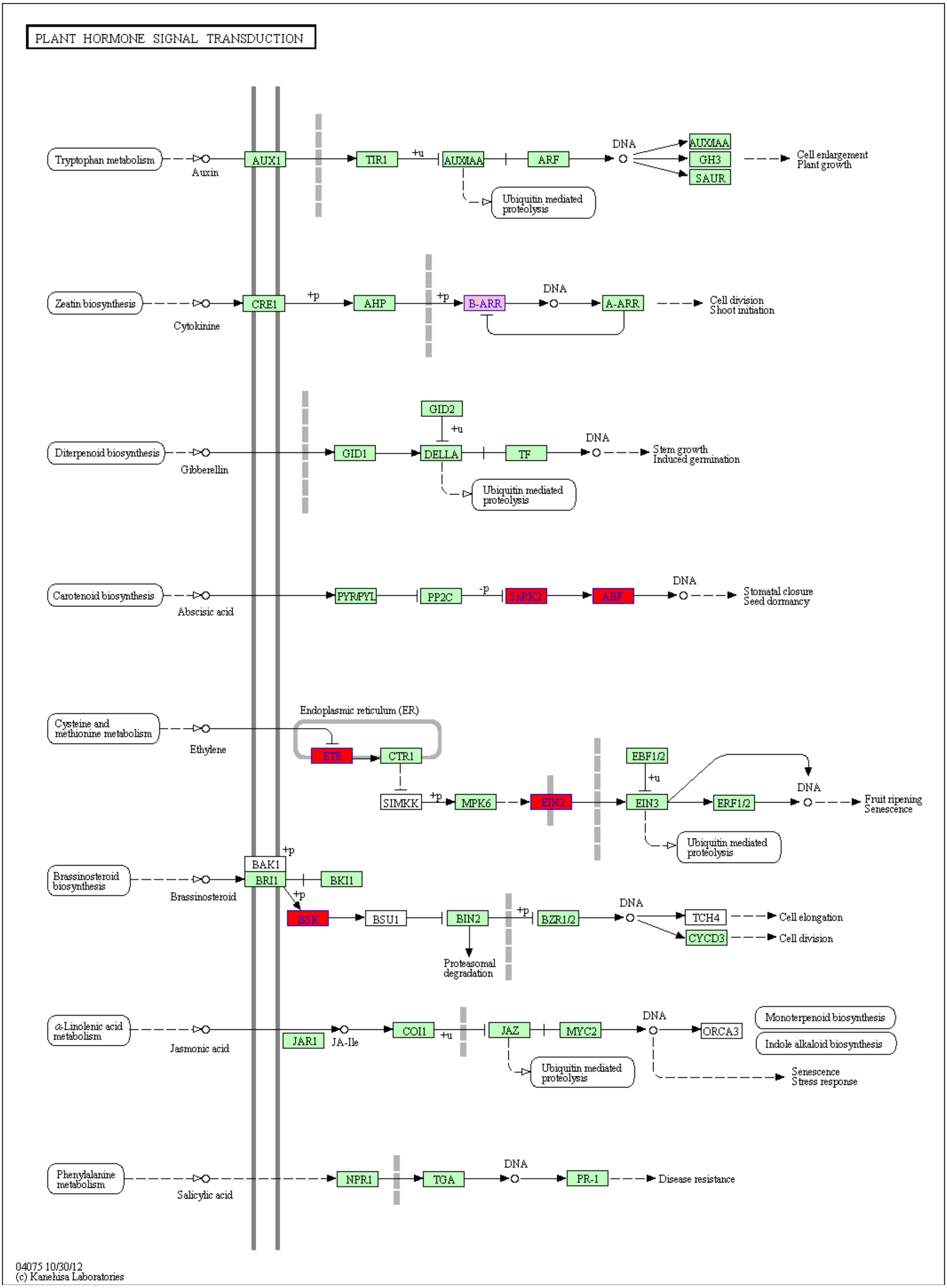
PLANT HORMONE SIGNAL TRANSDUCTION
Tryptophan metabolism
Auxin
AUX1
TIR1
+u
AUX/IAA
ARF
DNA
AUX/IAA
GH3
SAUR
Cell enlargement
Plant growth
Ubiquitin mediated proteolysis
Zeatin biosynthesis
Cytokinine
CRE1
+p
AHP
+p
B-ARR
DNA
A-ARR
Cell division
Shoot initiation
Diterpenoid biosynthesis
Gibberellin
GID2
+u
GID1
DELLA
TF
DNA
Stem growth
Induced germination
Ubiquitin mediated proteolysis
Carotenoid biosynthesis
Abscisic acid
PYR/PYL
PP2C
-p
SnRK2
ABF
DNA
Stomatal closure
Seed dormancy
Cysteine and methionine metabolism
Ethylene
Endoplasmic reticulum (ER)
ETR
CTR1
SIMKK
+p
MPK6
EIN2
EBF1/2
+u
EIN3
ERF1/2
DNA
Fruit ripening
Senescence
Ubiquitin mediated proteolysis
Brassinosteroid biosynthesis
Brassinosteroid
BAK1
+p
BRI1
BKI1
+p
BSK
BSU1
BIN2
+p
BZR1/2
DNA
TCH4
Cell elongation
CYCD3
Cell division
Proteasomal degradation
α-Linolenic acid metabolism
Jasmonic acid
JAR1
JA-Ile
COI1
+u
JAZ
MYC2
DNA
ORCA3
Monoterpenoid biosynthesis
Indole alkaloid biosynthesis
Ubiquitin mediated proteolysis
Senescence
Stress response
Phenylalanine metabolism
Salicylic acid
NPR1
TGA
DNA
PR-1
Disease resistance
04075 10/30/12
(c) Kanehisa Laboratories

R

OXIDATIVE PHOSPHORYLATION

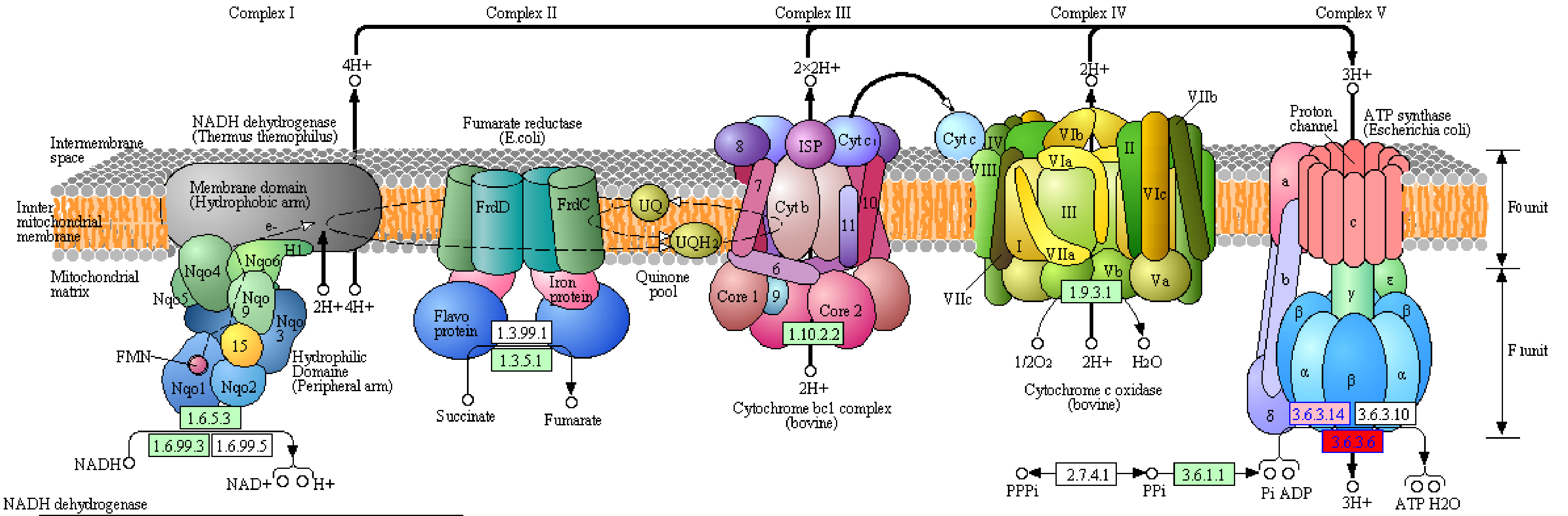

NADH dehydrogenase

E	ND1	ND2	ND3	ND4	ND4L	ND5	ND6										
E	Ndufs1	Ndufs2	Ndufs3	Ndufs4	Ndufs5	Ndufs6	Ndufs7	Ndufs8	Ndufv1	Ndufv2	Ndufv3						
B/A	NuoA	NuoB	NuoC	NuoD	NuoE	NuoF	NuoG	NuoH	NuoI	NuoJ	NuoK	NuoL	NuoM	NuoN			
B/A	NdhC	NdhK	NdhJ	NdhH	NdhA	NdhI	NdhG	NdhE	NdhF	NdhD	NdhB	NdhL	NdhM	NdhN	HoxE	HoxF	HoxU
E	Ndufa1	Ndufa2	Ndufa3	Ndufa4	Ndufa5	Ndufa6	Ndufa7	Ndufa8	Ndufa9	Ndufa10	Ndufab1	Ndufa11	Ndufa12	Ndufa13			
E	Ndufb1	Ndufb2	Ndufb3	Ndufb4	Ndufb5	Ndufb6	Ndufb7	Ndufb8	Ndufb9	Ndufb10	Ndufb11	Ndufc1	Ndufc2				

Succinate dehydrogenase / Fumarate reductase

E	SDHC	SDHD	SDHA	SDHB		
B/A	SdhC	SdhD	SdhA	SdhB		
			FrdA	FrdB	FrdC	FrdD

Cytochrome c reductase

E/B/A	ISP	Cytb	Cyt1						
E				COR1	QCR2	QCR6	QCR7	QCR8	QCR9	QCR10

Cytochrome c oxidase

E	COX10		COX3	COX1	COX2	COX4	COX5A	COX5B	COX6A	COX6B	COX6C	COX7A	COX7B	COX7C	COX8	COX17
E/B/A	COX11	COX15														
B/A	CyoE	CyoD	CyoC	CyoB	CyoA											
		CoxD	CoxC	CoxA	CoxB											
		QoxD	QoxC	QoxB	QoxA											

Cytochrome c oxidase , cbb3-type

B	I	II	IV	III

Cytochrome bd complex

B/A	CydA	CydB

F-type ATPase (Bacteria)

beta	alpha	gamma	delta	epsilon	c	a	b

F-type ATPase (Eukaryotes)

beta	alpha	gamma	OSCP	delta	epsilon	c	a
b	e	f6	f	8			
d	f	h	j	k	g		

V-type ATPase (Prokaryotes)

A	B	C	D	E	F	I	K

V-type ATPase (Eukaryotes)

A	B	C	D	E	F	G	H
I	AC39	54kD	S1	lipid			

00190 6/1/12
(c) Kanehisa Laboratories

S

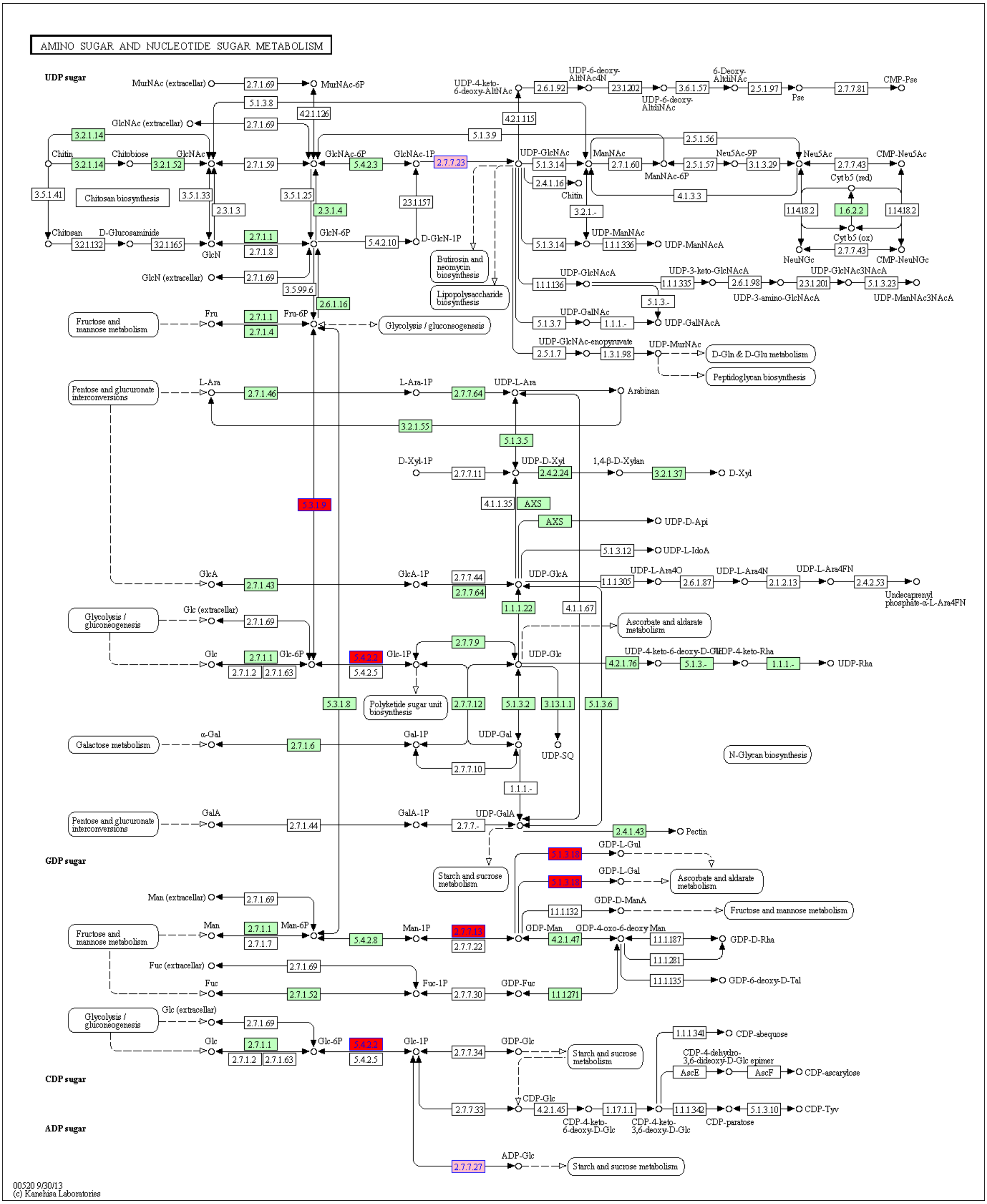
AMINO SUGAR AND NUCLEOTIDE SUGAR METABOLISM
UDP sugar
GDP sugar
CDP sugar
ADP sugar
MurNAc (extracellar)
MurNAc-6P
GlcNAc (extracellar)
Chitin
Chitobiose
GlcNAc
GlcNAc-6P
GlcNAc-1P
UDP-GlcNAc
ManNAc
ManNAc-6P
Neu5Ac-9P
Neu5Ac
CMP-Neu5Ac
Cyt b5 (red)
Cyt b5 (ox)
NeuNGc
CMP-NeuNGc
UDP-4-keto-6-deoxy-AltNAc
UDP-6-deoxy-AltNAc4N
UDP-6-deoxy-AltdiNAc
6-Deoxy-AltdiNAc
Pse
CMP-Pse
Chitosan biosynthesis
Chitosan
D-Glucosaminide
GlcN
GlcN-6P
D-GlcN-1P
GlcN (extracellar)
Butirosin and neomycin biosynthesis
Lipopolysaccharide biosynthesis
UDP-ManNAc
UDP-ManNAcA
UDP-GlcNAcA
UDP-3-keto-GlcNAcA
UDP-3-amino-GlcNAcA
UDP-GlcNAc3NAcA
UDP-ManNAc3NAcA
UDP-GalNAc
UDP-GalNAcA
UDP-GlcNAc-enolpyruvate
UDP-MurNAc
D-Gln & D-Glu metabolism
Peptidoglycan biosynthesis
Fructose and mannose metabolism
Fru
Fru-6P
Glycolysis / gluconeogenesis
Pentose and glucuronate interconversions
L-Ara
L-Ara-1P
UDP-L-Ara
Arabinan
D-Xyl-1P
UDP-D-Xyl
1,4-β-D-Xylan
D-Xyl
UDP-D-Api
UDP-L-IdoA
GlcA
GlcA-1P
UDP-GlcA
UDP-L-Ara4O
UDP-L-Ara4N
UDP-L-Ara4FN
Undecaprenyl phosphate-α-L-Ara4FN
Glc (extracellar)
Ascorbate and aldarate metabolism
Glc
Glc-6P
Glc-1P
UDP-Glc
UDP-4-keto-6-deoxy-D-Glc
UDP-4-keto-Rha
UDP-Rha
Polyketide sugar unit biosynthesis
UDP-SQ
Galactose metabolism
α-Gal
Gal-1P
UDP-Gal
N-Glycan biosynthesis
GalA
GalA-1P
UDP-GalA
Pectin
Starch and sucrose metabolism
GDP-L-Gul
GDP-L-Gal
GDP-D-ManA
Man (extracellar)
Man
Man-6P
Man-1P
GDP-Man
GDP-4-oxo-6-deoxy Man
GDP-D-Rha
GDP-6-deoxy-D-Tal
Fuc (extracellar)
Fuc
Fuc-1P
GDP-Fuc
GDP-Glc
CDP-Glc
CDP-4-keto-6-deoxy-D-Glc
CDP-4-keto-3,6-dideoxy-D-Glc
CDP-paratose
CDP-abequose
CDP-4-dehydro-3,6-dideoxy-D-Glc epimer
CDP-ascarylose
CDP-Tyv
ADP-Glc
AscE
AscF
AXS
2.7.1.69
5.1.3.8
4.2.1.126
4.2.1.115
3.2.1.14
3.2.1.52
2.7.1.59
5.4.2.3
2.7.7.23
5.1.3.9
2.5.1.56
5.1.3.14
2.7.1.60
2.5.1.57
3.1.3.29
2.7.7.43
2.6.1.92
2.3.1.202
3.6.1.57
2.5.1.97
2.7.7.81
3.5.1.41
3.5.1.33
3.5.1.25
2.3.1.3
2.3.1.4
2.3.1.157
2.4.1.16
4.1.3.3
3.2.1.-
1.14.18.2
1.6.2.2
3.2.1.132
3.2.1.165
2.7.1.1
2.7.1.8
5.4.2.10
1.1.1.336
1.1.1.136
1.1.1.335
2.6.1.98
2.3.1.201
5.1.3.23
3.5.99.6
2.6.1.16
2.7.1.4
5.1.3.7
1.1.1.-
5.1.3.-
2.5.1.7
1.3.1.98
2.7.1.46
2.7.7.64
3.2.1.55
5.1.3.5
2.7.7.11
2.4.2.24
3.2.1.37
5.3.1.9
4.1.1.35
5.1.3.12
2.7.1.43
2.7.7.44
1.1.1.22
4.1.1.67
1.1.1.305
2.6.1.87
2.1.2.13
2.4.2.53
2.7.7.9
5.4.2.2
5.4.2.5
2.7.1.2
2.7.1.63
4.2.1.76
5.1.3.-
1.1.1.-
5.3.1.8
2.7.7.12
5.1.3.2
3.13.1.1
5.1.3.6
2.7.1.6
2.7.7.10
2.7.1.44
2.7.7.-
2.4.1.43
5.1.3.18
1.1.1.132
2.7.1.7
5.4.2.8
2.7.7.13
2.7.7.22
4.2.1.47
1.1.1.187
1.1.1.281
1.1.1.135
2.7.1.52
2.7.7.30
1.1.1.271
2.7.7.34
1.1.1.341
2.7.7.33
4.2.1.45
1.17.1.1
1.1.1.342
5.1.3.10
2.7.7.27
00520 9/30/13
(c) Kanehisa Laboratories

T

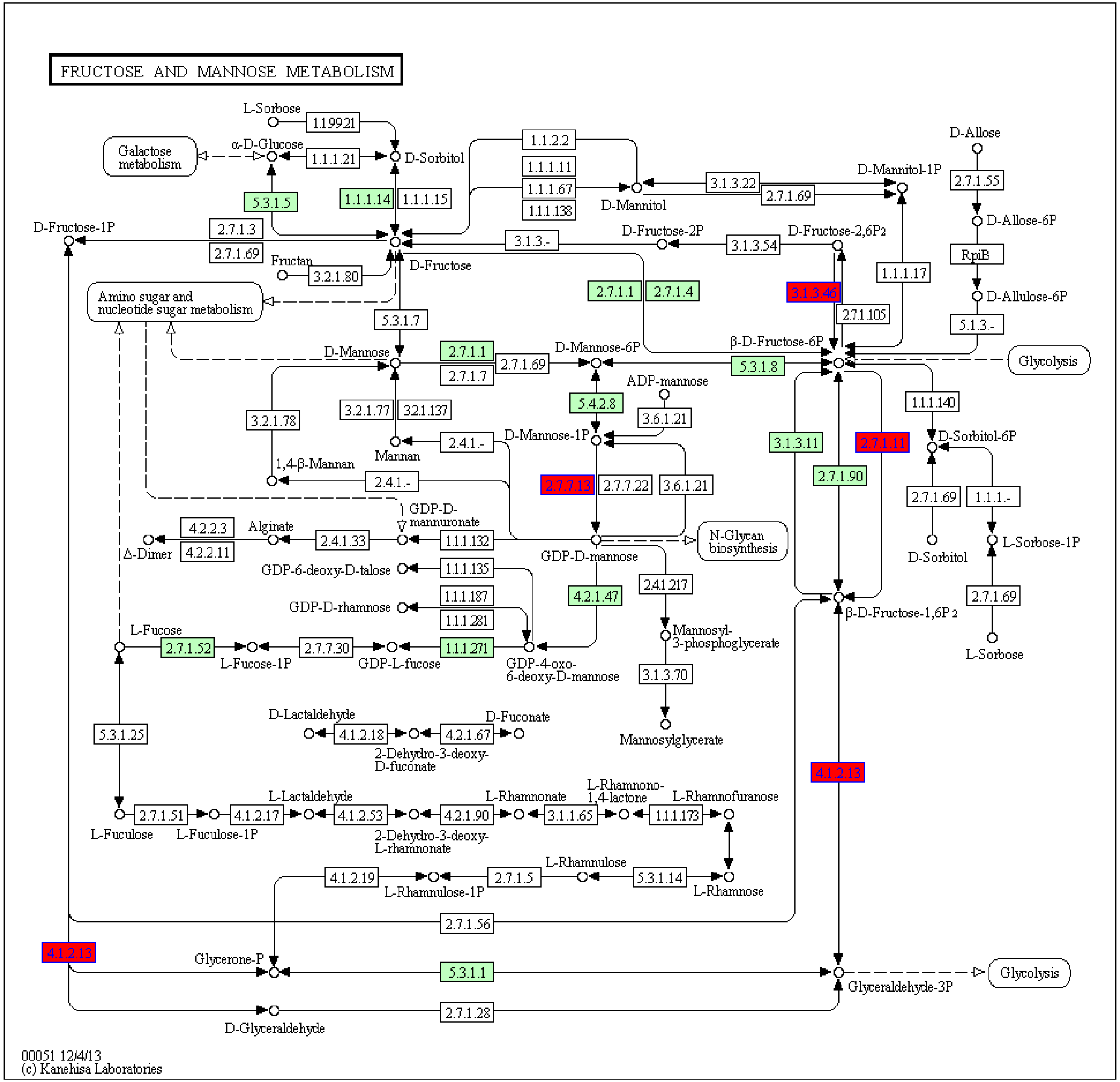
FRUCTOSE AND MANNOSE METABOLISM
L-Sorbose
1.1.99.21
Galactose metabolism
α-D-Glucose
1.1.1.21
D-Sorbitol
1.1.2.2
1.1.1.11
1.1.1.67
1.1.1.138
D-Mannitol
3.1.3.22
2.7.1.69
D-Mannitol-1P
D-Allose
2.7.1.55
D-Allose-6P
RpiB
D-Allulose-6P
5.1.3.-
5.3.1.5
1.1.1.14
1.1.1.15
D-Fructose-1P
2.7.1.3
2.7.1.69
3.1.3.-
D-Fructose-2P
3.1.3.54
D-Fructose-2,6P2
Fructan
3.2.1.80
D-Fructose
2.7.1.1
2.7.1.4
3.1.3.46
2.7.1.105
1.1.1.17
Amino sugar and nucleotide sugar metabolism
5.3.1.7
D-Mannose
2.7.1.1
2.7.1.7
2.7.1.69
D-Mannose-6P
β-D-Fructose-6P
5.3.1.8
Glycolysis
ADP-mannose
5.4.2.8
3.6.1.21
3.2.1.78
3.2.1.77
3.2.1.137
2.4.1.-
D-Mannose-1P
1.1.1.140
D-Sorbitol-6P
3.1.3.11
2.7.1.11
Mannan
1,4-β-Mannan
2.4.1.-
2.7.7.13
2.7.7.22
3.6.1.21
2.7.1.90
2.7.1.69
1.1.1.-
GDP-D-mannuronate
Alginate
4.2.2.3
4.2.2.11
Δ-Dimer
2.4.1.33
1.1.1.132
GDP-D-mannose
N-Glycan biosynthesis
D-Sorbitol
L-Sorbose-1P
GDP-6-deoxy-D-talose
1.1.1.135
1.1.1.187
1.1.1.281
GDP-D-rhamnose
4.2.1.47
2.4.1.217
2.7.1.69
β-D-Fructose-1,6P2
L-Fucose
2.7.1.52
L-Fucose-1P
2.7.7.30
GDP-L-fucose
1.1.1.271
GDP-4-oxo-6-deoxy-D-mannose
Mannosyl-3-phosphoglycerate
L-Sorbose
3.1.3.70
Mannosylglycerate
5.3.1.25
D-Lactaldehyde
4.1.2.18
4.2.1.67
D-Fuconate
2-Dehydro-3-deoxy-D-fuconate
4.1.2.13
L-Fuculose
2.7.1.51
L-Fuculose-1P
4.1.2.17
L-Lactaldehyde
4.1.2.53
2-Dehydro-3-deoxy-L-rhamnonate
4.2.1.90
L-Rhamnonate
3.1.1.65
L-Rhamnono-1,4-lactone
1.1.1.173
L-Rhamnofuranose
4.1.2.19
L-Rhamnulose-1P
2.7.1.5
L-Rhamnulose
5.3.1.14
L-Rhamnose
2.7.1.56
4.1.2.13
Glycerone-P
5.3.1.1
Glyceraldehyde-3P
Glycolysis
D-Glyceraldehyde
2.7.1.28
00051 12/4/13
(c) Kanehisa Laboratories

U

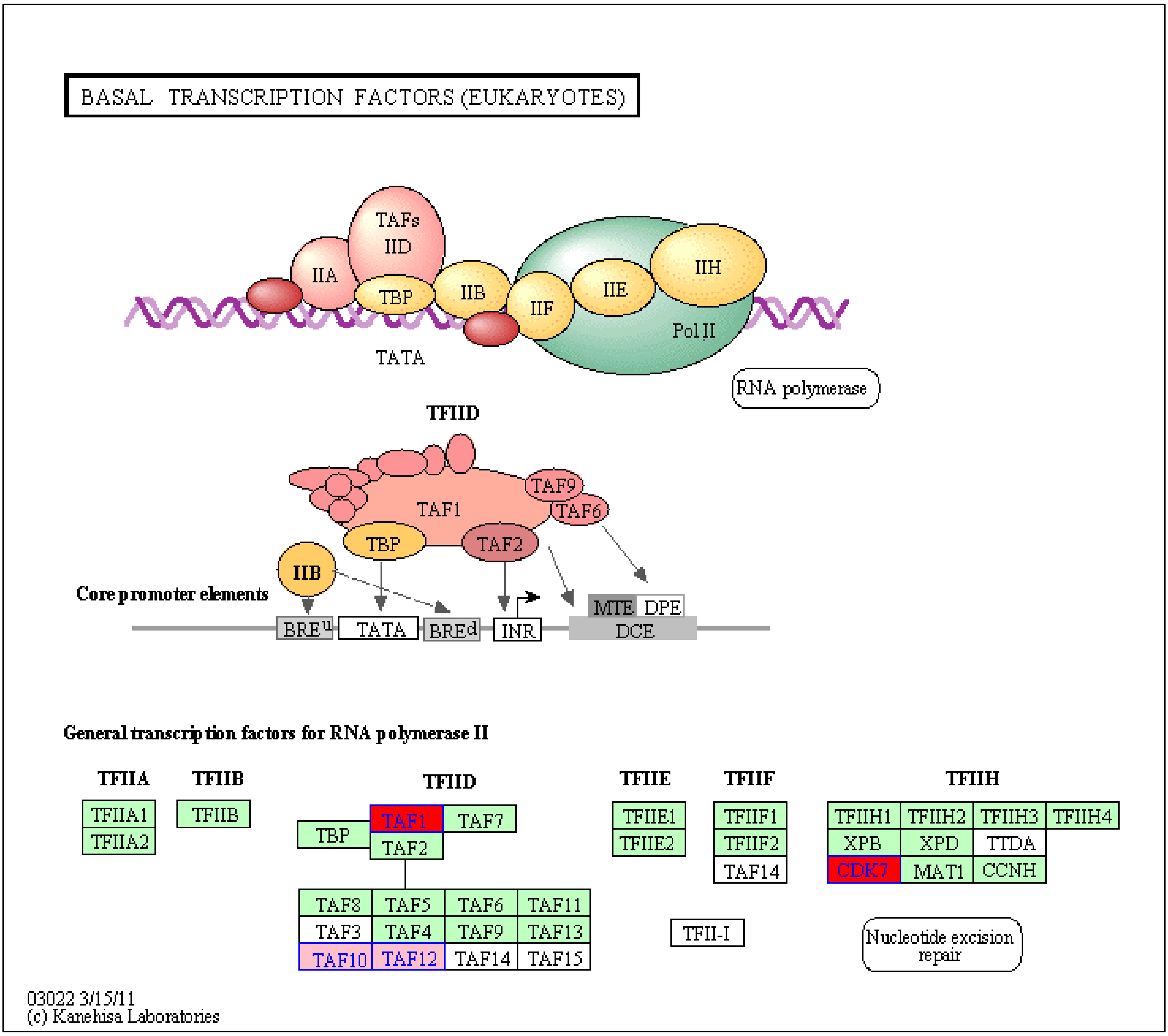
BASAL TRANSCRIPTION FACTORS (EUKARYOTES)
TAFs
IID
IIA
TBP
IIB
IIF
IIE
IIH
Pol II
TATA
RNA polymerase
TFIID
TAF9
TAF1
TAF6
TBP
TAF2
IIB
Core promoter elements
BRE u
TATA
BRE d
INR
MTE
DPE
DCE
General transcription factors for RNA polymerase II
TFIIA
TFIIB
TFIID
TFIIE
TFIIF
TFIIH
TFIIA1
TFIIA2
TFIIB
TBP
TAF1
TAF7
TAF2
TAF8
TAF5
TAF6
TAF11
TAF3
TAF4
TAF9
TAF13
TAF10
TAF12
TAF14
TAF15
TFIIE1
TFIIE2
TFIIF1
TFIIF2
TAF14
TFII-I
TFIIH1
TFIIH2
TFIIH3
TFIIH4
XPB
XPD
TTDA
CDK7
MAT1
CCNH
Nucleotide excision repair
03022 3/15/11
(c) Kanehisa Laboratories

V

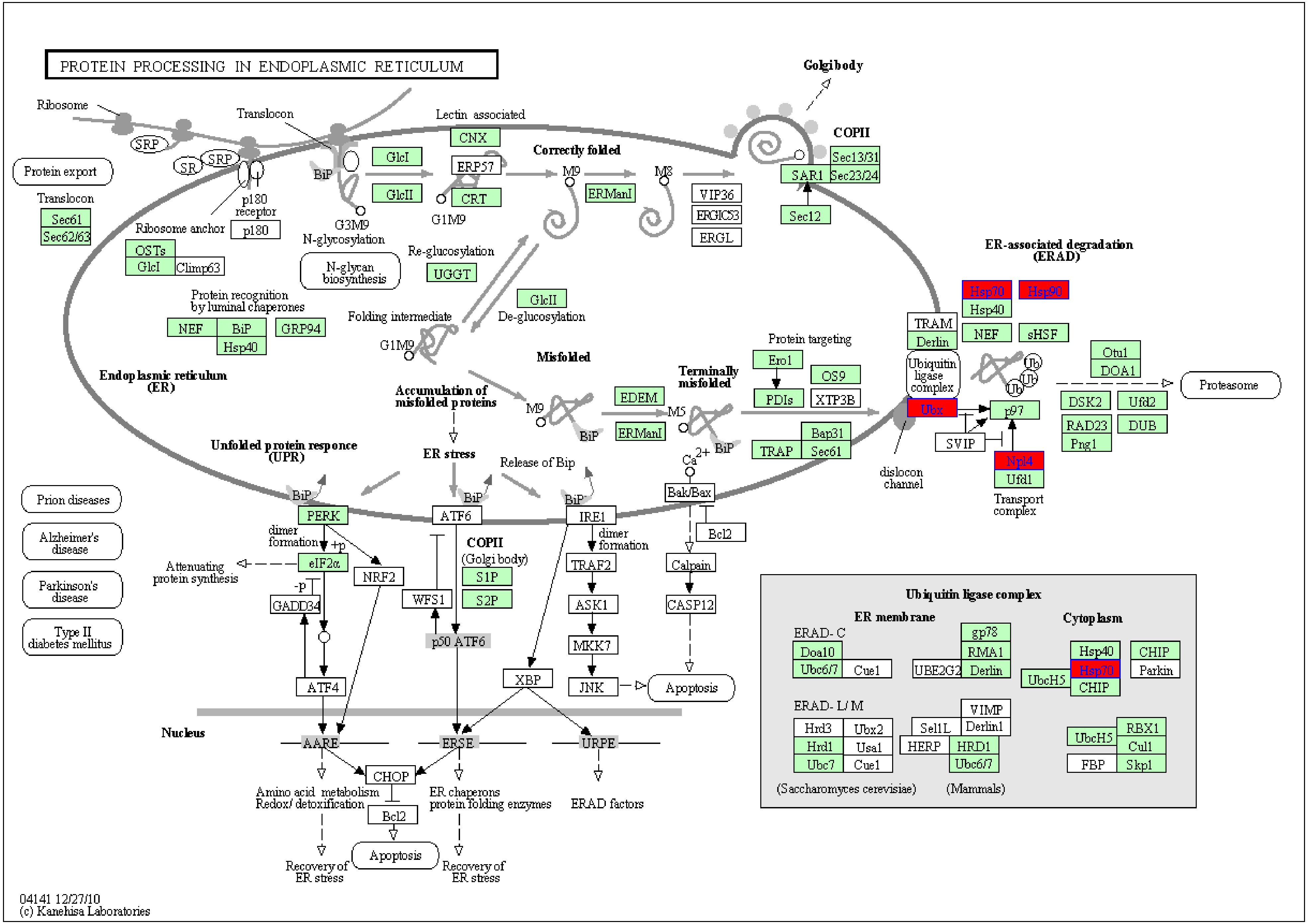
PROTEIN PROCESSING IN ENDOPLASMIC RETICULUM
Ribosome
Translocon
SRP
SR
p180 receptor
Protein export
Sec61
Sec62/63
Ribosome anchor
p180
OSTs
GlcI
Climp63
BiP
G3M9
N-glycosylation
N-glycan biosynthesis
Lectin associated
CNX
GlcI
GlcII
ERP57
CRT
G1M9
Correctly folded
M9
M8
ERManI
VIP36
ERGIC53
ERGL
Golgi body
COPII
Sec13/31
SAR1
Sec23/24
Sec12
Re-glucosylation
UGGT
GlcII
De-glucosylation
Protein recognition by luminal chaperones
NEF
BiP
Hsp40
GRP94
Folding intermediate
Endoplasmic reticulum (ER)
Misfolded
Accumulation of misfolded proteins
Terminally misfolded
Protein targeting
Ero1
PDIs
OS9
XTP3B
EDEM
ERManI
M5
Bap31
TRAP
Sec61
Ca2+
ER-associated degradation (ERAD)
Hsp70
Hsp90
Hsp40
NEF
sHSF
TRAM
Derlin
Ubiquitin ligase complex
Ub
Otu1
DOA1
Proteasome
Ubx
p97
SVIP
Npl4
Ufd1
Transport complex
DSK2
Ufd2
RAD23
DUB
Png1
dislocon channel
Unfolded protein responce (UPR)
ER stress
Release of Bip
Prion diseases
Alzheimer's disease
Parkinson's disease
Type II diabetes mellitus
PERK
dimer formation
+p
eIF2α
Attenuating protein synthesis
NRF2
-p
GADD34
ATF4
ATF6
WFS1
COPII (Golgi body)
S1P
S2P
p50 ATF6
IRE1
dimer formation
TRAF2
ASK1
MKK7
JNK
XBP
Apoptosis
Bak/Bax
Bcl2
Calpain
CASP12
Nucleus
AARE
ERSE
URPE
CHOP
Amino acid metabolism Redox/ detoxification
Bcl2
Apoptosis
ER chaperons protein folding enzymes
ERAD factors
Recovery of ER stress
Recovery of ER stress
Ubiquitin ligase complex
ER membrane
Cytoplasm
ERAD- C
Doa10
Ubc6/7
Cue1
gp78
RMA1
UBE2G2
Derlin
Hsp40
Hsp70
UbcH5
CHIP
CHIP
Parkin
ERAD- L/ M
Hrd3
Ubx2
Hrd1
Usa1
Ubc7
Cue1
VIMP
Sel1L
Derlin1
HERP
HRD1
Ubc6/7
UbcH5
RBX1
Cul1
FBP
Skp1
(Saccharomyces cerevisiae)
(Mammals)
04141 12/27/10
(c) Kanehisa Laboratories

W

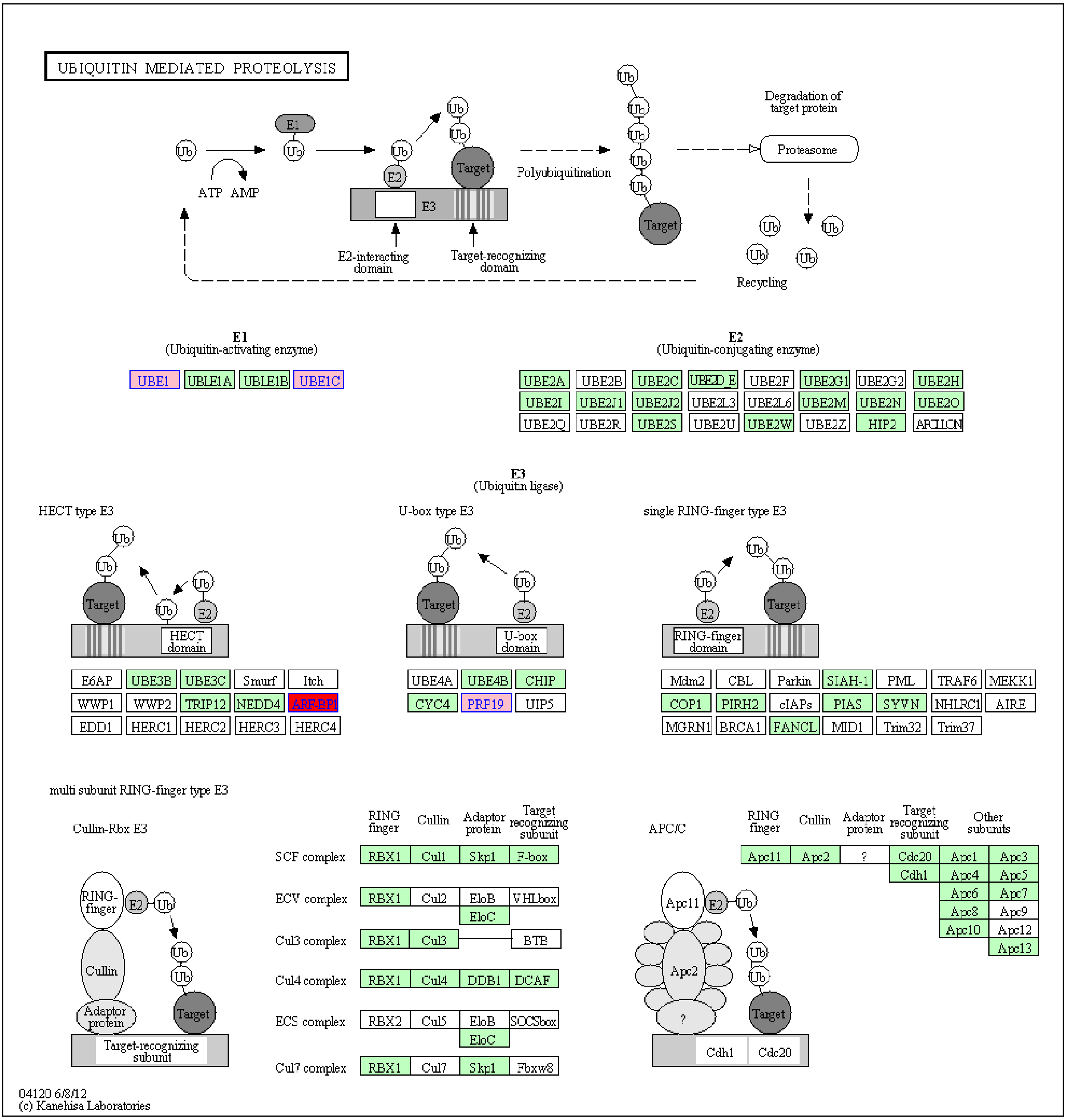
UBIQUITIN MEDIATED PROTEOLYSIS
E1
Ub
ATP AMP
E2
E3
Target
E2-interacting domain
Target-recognizing domain
Polyubiquitination
Degradation of target protein
Proteasome
Recycling
E1
(Ubiquitin-activating enzyme)
UBE1
UBLE1A
UBLE1B
UBE1C
E2
(Ubiquitin-conjugating enzyme)
UBE2A
UBE2B
UBE2C
UBE2D_E
UBE2F
UBE2G1
UBE2G2
UBE2H
UBE2I
UBE2J1
UBE2J2
UBE2L3
UBE2L6
UBE2M
UBE2N
UBE2O
UBE2Q
UBE2R
UBE2S
UBE2U
UBE2W
UBE2Z
HIP2
APOLLON
E3
(Ubiquitin ligase)
HECT type E3
HECT domain
E6AP
UBE3B
UBE3C
Smurf
Itch
WWP1
WWP2
TRIP12
NEDD4
ARFBP1
EDD1
HERC1
HERC2
HERC3
HERC4
U-box type E3
U-box domain
UBE4A
UBE4B
CHIP
CYC4
PRP19
UIP5
single RING-finger type E3
RING-finger domain
Mdm2
CBL
Parkin
SIAH-1
PML
TRAF6
MEKK1
COP1
PIRH2
cIAPs
PIAS
SYVN
NHLRC1
AIRE
MGRN1
BRCA1
FANCL
MID1
Trim32
Trim37
multi subunit RING-finger type E3
Cullin-Rbx E3
RING-finger
Cullin
Adaptor protein
Target-recognizing subunit
RING finger
Cullin
Adaptor protein
Target recognizing subunit
SCF complex
RBX1
Cul1
Skp1
F-box
ECV complex
RBX1
Cul2
EloB
VHLbox
EloC
Cul3 complex
RBX1
Cul3
BTB
Cul4 complex
RBX1
Cul4
DDB1
DCAF
ECS complex
RBX2
Cul5
EloB
SOCSbox
EloC
Cul7 complex
RBX1
Cul7
Skp1
Fbxw8
APC/C
RING finger
Cullin
Adaptor protein
Target recognizing subunit
Other subunits
Apc11
Apc2
?
Cdc20
Apc1
Apc3
Cdh1
Apc4
Apc5
Apc6
Apc7
Apc8
Apc9
Apc10
Apc12
Apc13
Cdh1
Cdc20
04120 6/8/12
(c) Kanehisa Laboratories

X

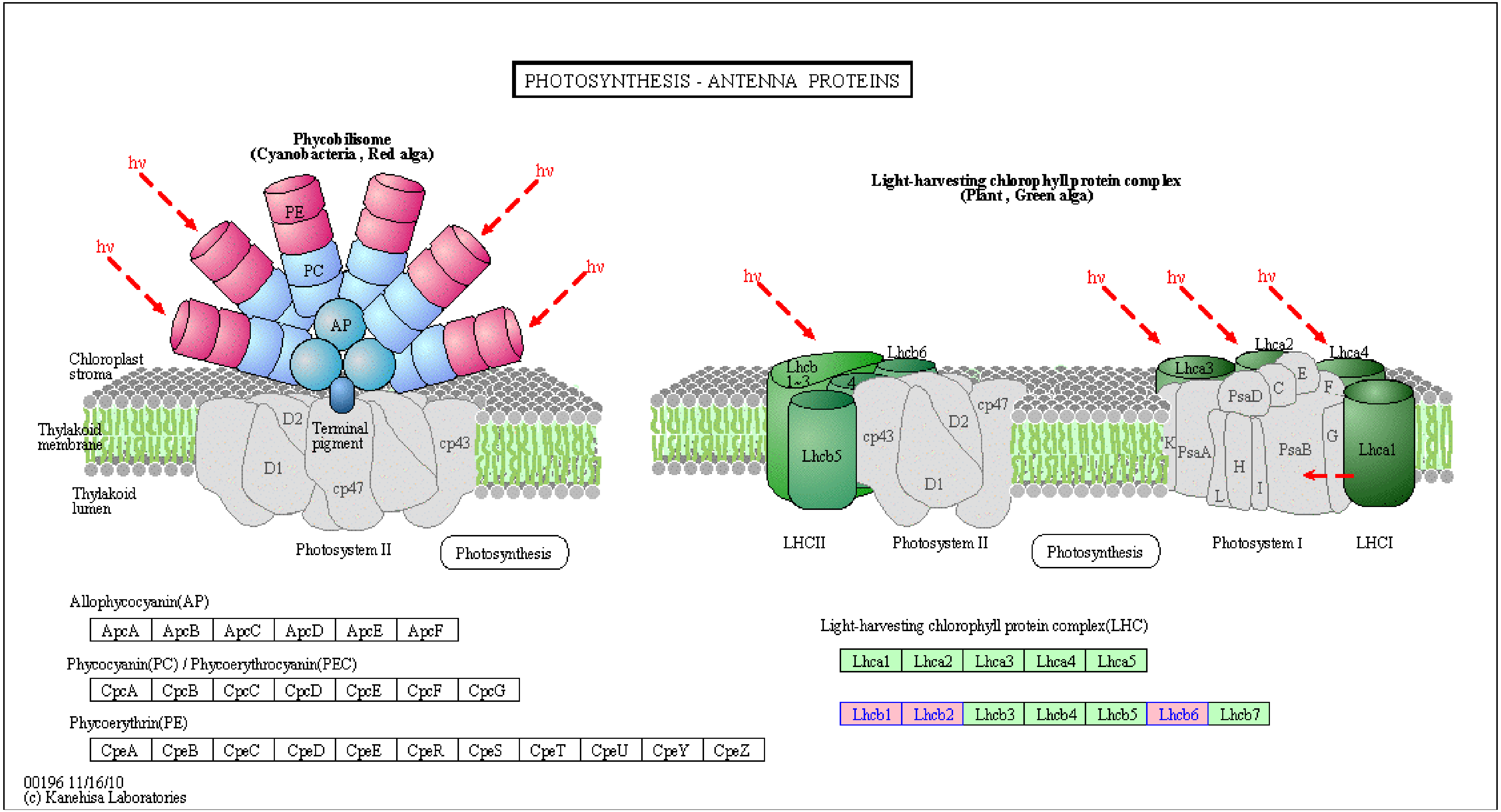
PHOTOSYNTHESIS - ANTENNA PROTEINS
Phycobilisome
(Cyanobacteria, Red alga)
hv
PE
PC
AP
Chloroplast stroma
Thylakoid membrane
Thylakoid lumen
D2
D1
Terminal pigment
cp47
cp43
Photosystem II
Photosynthesis
Light-harvesting chlorophyll protein complex
(Plant, Green alga)
Lhcb 1~3
4
Lhcb6
Lhcb5
cp43
cp47
D2
D1
Lhca3
Lhca2
Lhca4
PsaD
C
E
F
K
PsaA
H
L
I
PsaB
G
Lhca1
LHCII
Photosystem II
Photosynthesis
Photosystem I
LHCI
Allophycocyanin(AP)
ApcA ApcB ApcC ApcD ApcE ApcF
Phycocyanin(PC) / Phycoerythrocyanin(PEC)
CpcA CpcB CpcC CpcD CpcE CpcF CpcG
Phycoerythrin(PE)
CpeA CpeB CpeC CpeD CpeE CpeR CpeS CpeT CpeU CpeY CpeZ
Light-harvesting chlorophyll protein complex(LHC)
Lhca1 Lhca2 Lhca3 Lhca4 Lhca5
Lhcb1 Lhcb2 Lhcb3 Lhcb4 Lhcb5 Lhcb6 Lhcb7
00196 11/16/10
(c) Kanehisa Laboratories

Y

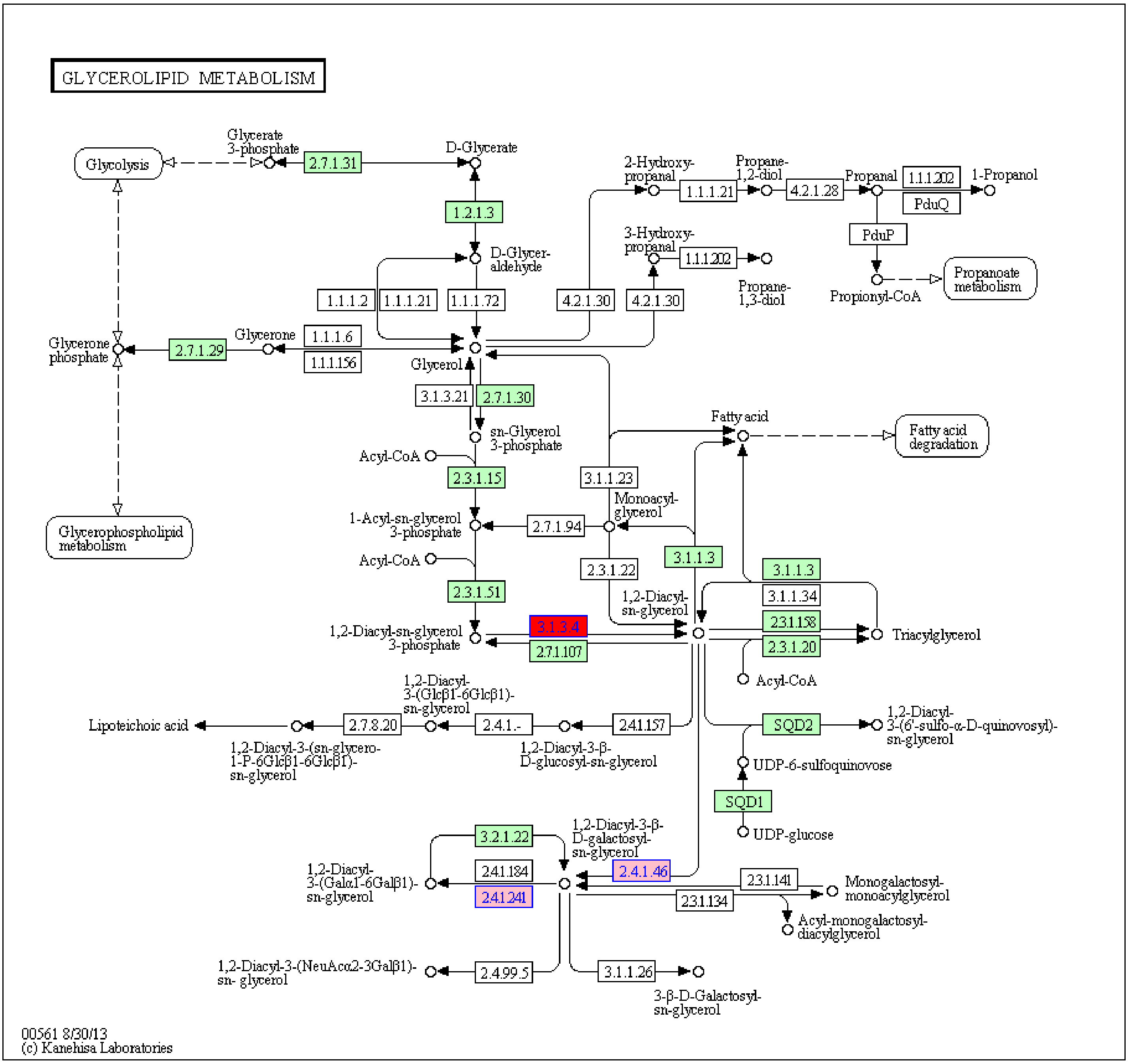
GLYCEROLIPID METABOLISM
Glycolysis
Glycerate 3-phosphate
2.7.1.31
D-Glycerate
1.2.1.3
D-Glycer-aldehyde
1.1.1.2
1.1.1.21
1.1.1.72
4.2.1.30
4.2.1.30
2-Hydroxy-propanal
1.1.1.21
Propane-1,2-diol
4.2.1.28
Propanal
1.1.1.202
PduQ
1-Propanol
PduP
Propionyl-CoA
Propanoate metabolism
3-Hydroxy-propanal
1.1.1.202
Propane-1,3-diol
Glycerone phosphate
2.7.1.29
Glycerone
1.1.1.6
1.1.1.156
Glycerol
3.1.3.21
2.7.1.30
sn-Glycerol 3-phosphate
Acyl-CoA
2.3.1.15
1-Acyl-sn-glycerol 3-phosphate
2.7.1.94
Monoacyl-glycerol
3.1.1.23
Fatty acid
Fatty acid degradation
Glycerophospholipid metabolism
Acyl-CoA
2.3.1.51
2.3.1.22
3.1.1.3
3.1.1.3
3.1.1.34
2.3.1.158
2.3.1.20
1,2-Diacyl-sn-glycerol
1,2-Diacyl-sn-glycerol 3-phosphate
3.1.3.4
2.7.1.107
Triacylglycerol
Acyl-CoA
1,2-Diacyl-3-(Glcβ1-6Glcβ1)-sn-glycerol
Lipoteichoic acid
2.7.8.20
2.4.1.-
2.4.1.157
1,2-Diacyl-3-(sn-glycero-1-P-6Glcβ1-6Glcβ1)-sn-glycerol
1,2-Diacyl-3-β-D-glucosyl-sn-glycerol
SQD2
1,2-Diacyl-3-(6'-sulfo-α-D-quinovosyl)-sn-glycerol
UDP-6-sulfoquinovose
SQD1
UDP-glucose
3.2.1.22
1,2-Diacyl-3-β-D-galactosyl-sn-glycerol
2.4.1.46
2.4.1.184
2.4.1.241
1,2-Diacyl-3-(Galα1-6Galβ1)-sn-glycerol
2.3.1.141
2.3.1.134
Monogalactosyl-monoacylglycerol
Acyl-monogalactosyl-diacylglycerol
1,2-Diacyl-3-(NeuAcα2-3Galβ1)-sn-glycerol
2.4.99.5
3.1.1.26
3-β-D-Galactosyl-sn-glycerol
00561 8/30/13
(c) Kanehisa Laboratories

Z

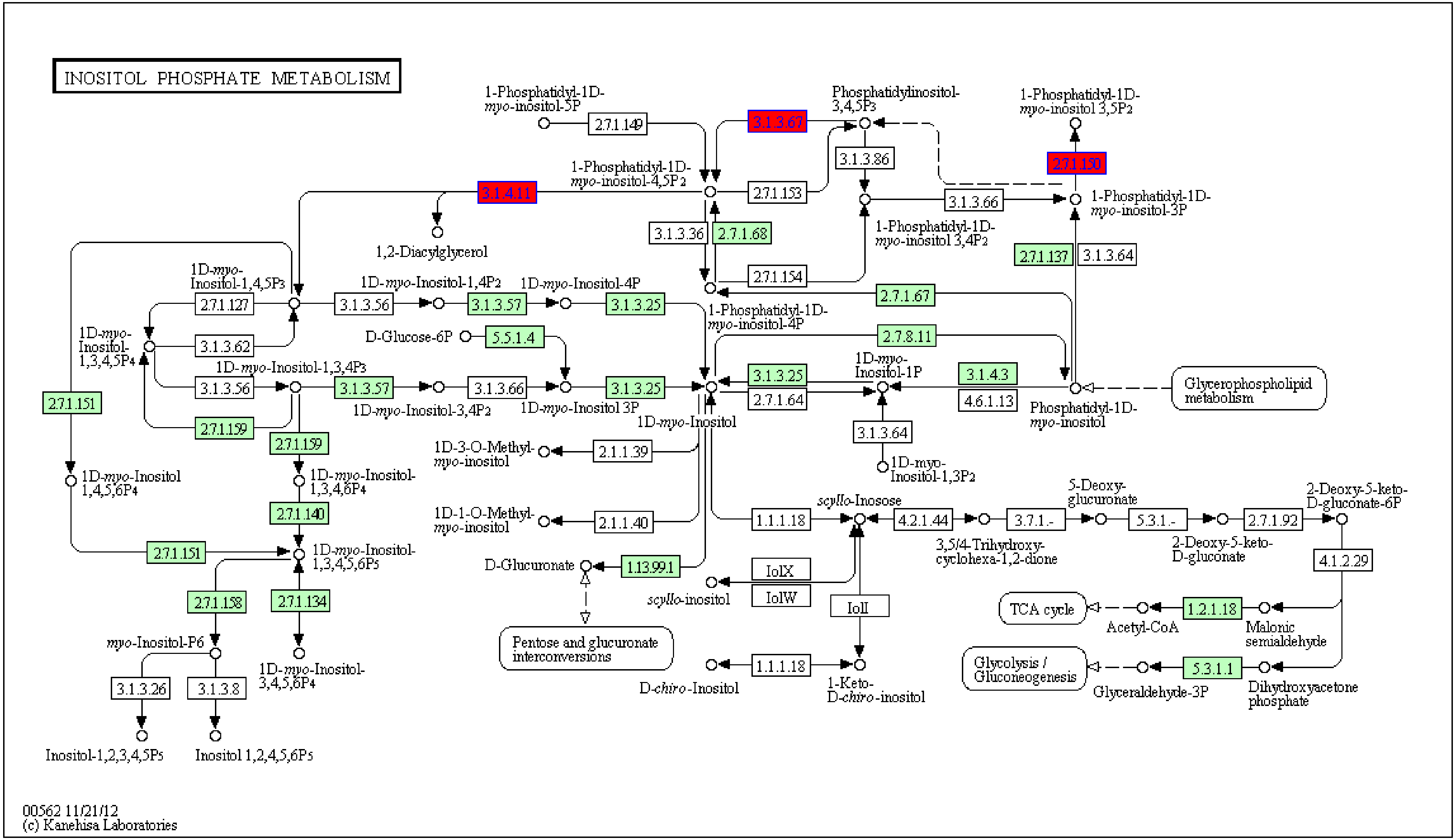
INOSITOL PHOSPHATE METABOLISM
1-Phosphatidyl-1D-myo-inositol-5P
2.7.1.149
3.1.3.67
Phosphatidylinositol-3,4,5P3
1-Phosphatidyl-1D-myo-inositol 3,5P2
2.7.1.150
3.1.3.86
1-Phosphatidyl-1D-myo-inositol-4,5P2
3.1.4.11
2.7.1.153
3.1.3.66
1-Phosphatidyl-1D-myo-inositol-3P
3.1.3.36
2.7.1.68
1-Phosphatidyl-1D-myo-inositol 3,4P2
2.7.1.137
3.1.3.64
1,2-Diacylglycerol
2.7.1.154
1D-myo-Inositol-1,4,5P3
1D-myo-Inositol-1,4P2
1D-myo-Inositol-4P
2.7.1.67
2.7.1.127
3.1.3.56
3.1.3.57
3.1.3.25
1-Phosphatidyl-1D-myo-inositol-4P
1D-myo-Inositol-1,3,4,5P4
3.1.3.62
D-Glucose-6P
5.5.1.4
2.7.8.11
1D-myo-Inositol-1,3,4P3
3.1.3.25
1D-myo-Inositol-1P
3.1.4.3
Glycerophospholipid metabolism
2.7.1.151
3.1.3.56
3.1.3.57
3.1.3.66
3.1.3.25
2.7.1.64
4.6.1.13
1D-myo-Inositol-3,4P2
1D-myo-Inositol 3P
Phosphatidyl-1D-myo-inositol
2.7.1.159
1D-myo-Inositol
2.7.1.159
3.1.3.64
1D-3-O-Methyl-myo-inositol
2.1.1.39
1D-myo-Inositol 1,4,5,6P4
1D-myo-Inositol-1,3,4,6P4
1D-myo-Inositol-1,3P2
5-Deoxy-glucuronate
2-Deoxy-5-keto-D-gluconate-6P
2.7.1.140
1D-1-O-Methyl-myo-inositol
2.1.1.40
scyllo-Inosose
1.1.1.18
4.2.1.44
3.7.1.-
5.3.1.-
2.7.1.92
2.7.1.151
1D-myo-Inositol-1,3,4,5,6P5
3,5/4-Trihydroxy-cyclohexa-1,2-dione
2-Deoxy-5-keto-D-gluconate
4.1.2.29
D-Glucuronate
1.13.99.1
IolX
IolW
scyllo-inositol
IolI
2.7.1.158
2.7.1.134
TCA cycle
Acetyl-CoA
1.2.1.18
Malonic semialdehyde
myo-Inositol-P6
Pentose and glucuronate interconversions
1D-myo-Inositol-3,4,5,6P4
1.1.1.18
1-Keto-D-chiro-inositol
D-chiro-Inositol
Glycolysis / Gluconeogenesis
5.3.1.1
Glyceraldehyde-3P
Dihydroxyacetone phosphate
3.1.3.26
3.1.3.8
Inositol-1,2,3,4,5P5
Inositol 1,2,4,5,6P5
00562 11/21/12
(c) Kanehisa Laboratories

AA

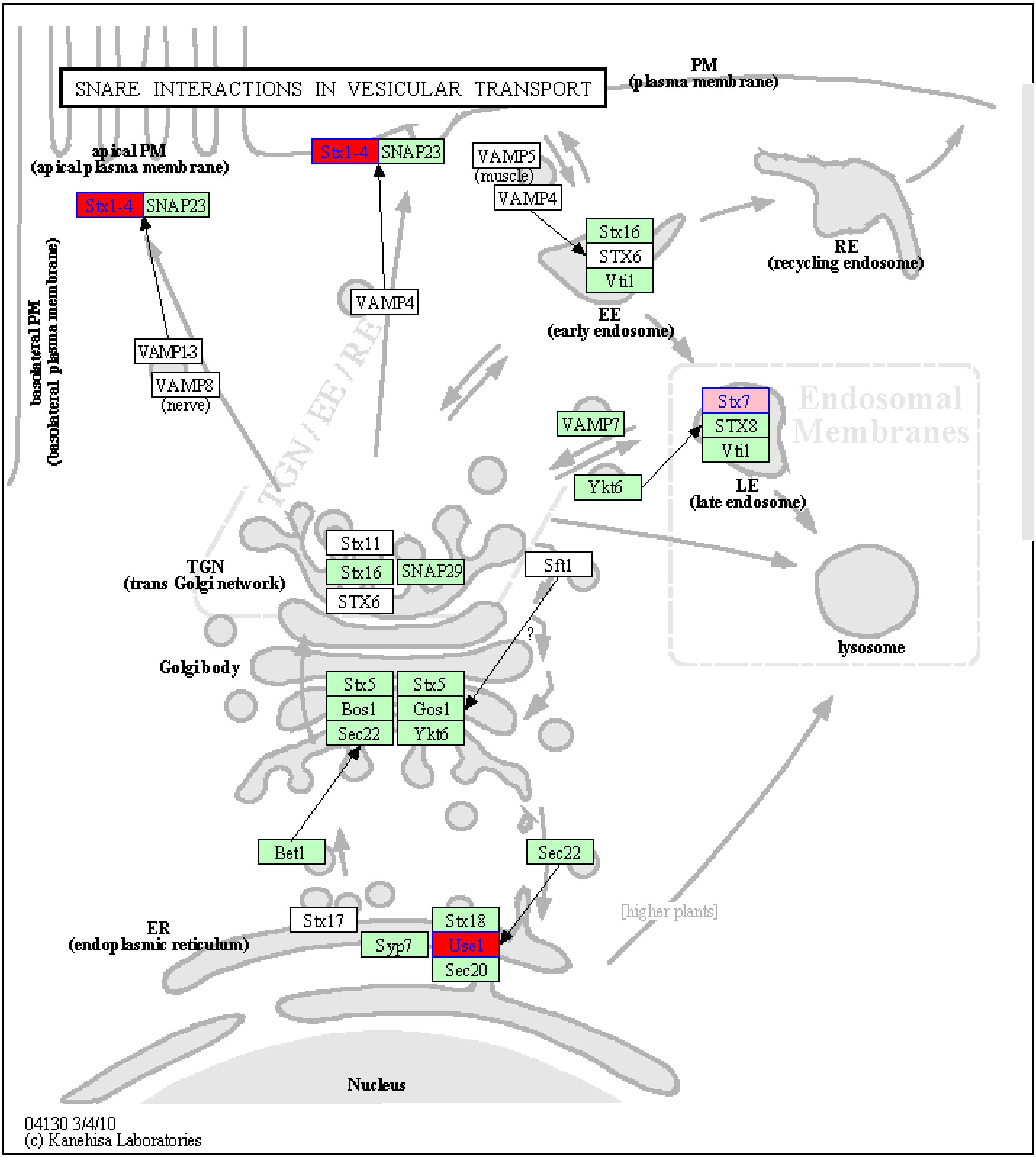
SNARE INTERACTIONS IN VESICULAR TRANSPORT
PM
(plasma membrane)
apical PM
(apical plasma membrane)
basolateral PM
(basolateral plasma membrane)
Stx1-4
SNAP23
Stx1-4
SNAP23
VAMP5
(muscle)
VAMP4
Stx16
STX6
Vti1
RE
(recycling endosome)
EE
(early endosome)
VAMP4
VAMP13
VAMP8
(nerve)
TGN / EE / RE
Endosomal Membranes
Stx7
STX8
Vti1
VAMP7
Ykt6
LE
(late endosome)
Stx11
Stx16
SNAP29
STX6
Sft1
TGN
(trans Golgi network)
?
lysosome
Golgi body
Stx5
Bos1
Sec22
Stx5
Gos1
Ykt6
Bet1
Sec22
[higher plants]
Stx17
Stx18
Use1
Sec20
Syp7
ER
(endoplasmic reticulum)
Nucleus
04130 3/4/10
(c) Kanehisa Laboratories

AB

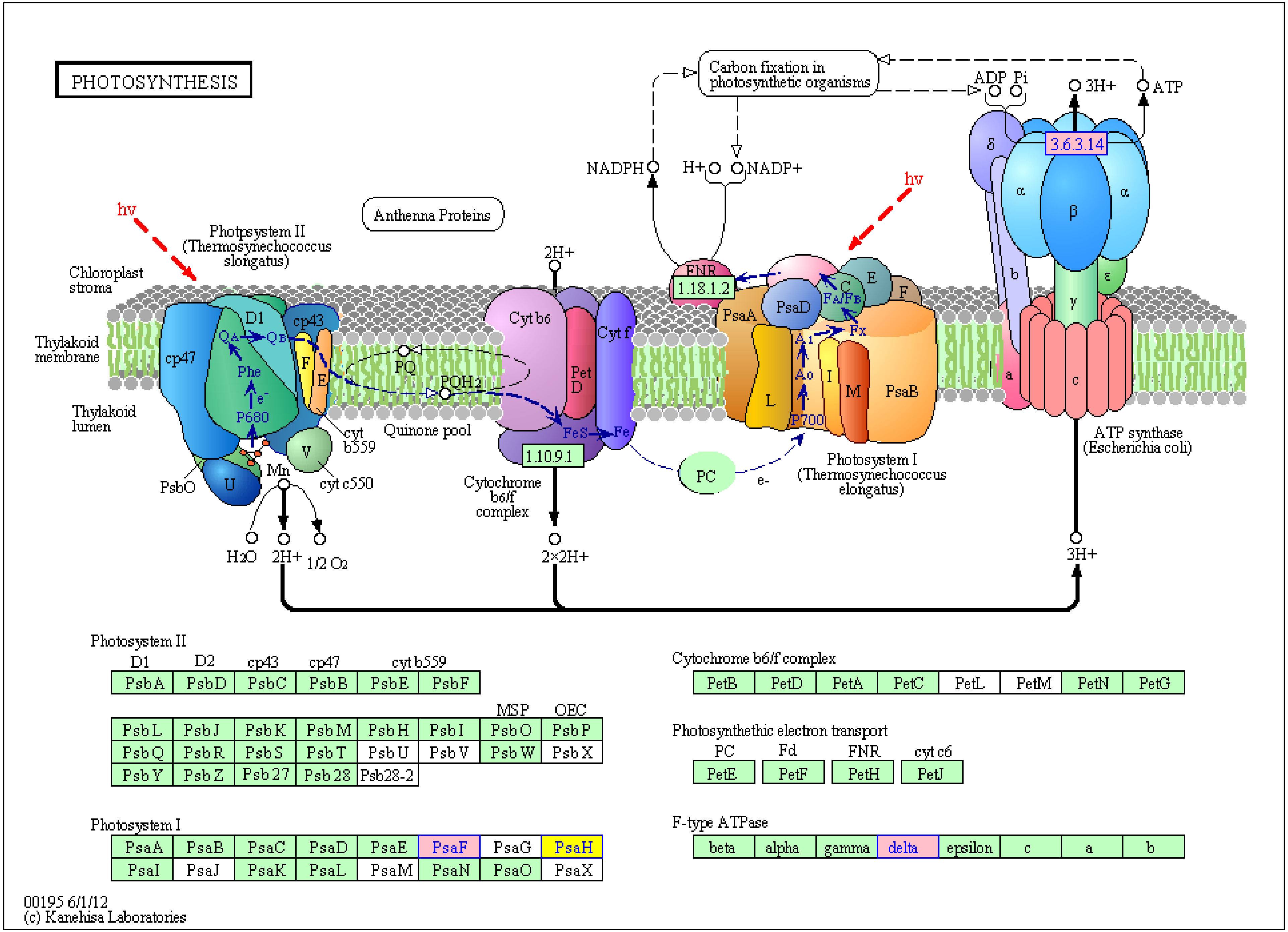
PHOTOSYNTHESIS
Carbon fixation in photosynthetic organisms
ADP Pi
3H+
ATP
NADPH
H+
NADP+
hv
Anthenna Proteins
Photpsystem II (Thermosynechococcus elongatus)
Chloroplast stroma
Thylakoid membrane
Thylakoid lumen
D1
cp43
cp47
QA
QB
Phe
e-
P680
F
E
V
U
PsbO
Mn
cyt b559
cyt c550
PQ
PQH2
Quinone pool
2H+
Cyt b6
Cyt f
Pet D
FeS
Fe
1.10.9.1
Cytochrome b6/f complex
FNR
1.18.1.2
PsaA
PsaD
C
E
F
FA/FB
Fx
A1
A0
P700
L
I
M
PsaB
PC
e-
Photosystem I (Thermosynechococcus elongatus)
3.6.3.14
δ
α
β
α
b
ε
γ
a
c
ATP synthase (Escherichia coli)
H2O
2H+
1/2 O2
2×2H+
3H+
Photosystem II
D1 D2 cp43 cp47 cyt b559
PsbA PsbD PsbC PsbB PsbE PsbF
MSP OEC
PsbL PsbJ PsbK PsbM PsbH PsbI PsbO PsbP
PsbQ PsbR PsbS PsbT PsbU PsbV PsbW PsbX
PsbY PsbZ Psb27 Psb28 Psb28-2
Photosystem I
PsaA PsaB PsaC PsaD PsaE PsaF PsaG PsaH
PsaI PsaJ PsaK PsaL PsaM PsaN PsaO PsaX
Cytochrome b6/f complex
PetB PetD PetA PetC PetL PetM PetN PetG
Photosynthethic electron transport
PC Fd FNR cyt c6
PetE PetF PetH PetJ
F-type ATPase
beta alpha gamma delta epsilon c a b
00195 6/1/12
(c) Kanehisa Laboratories

AC

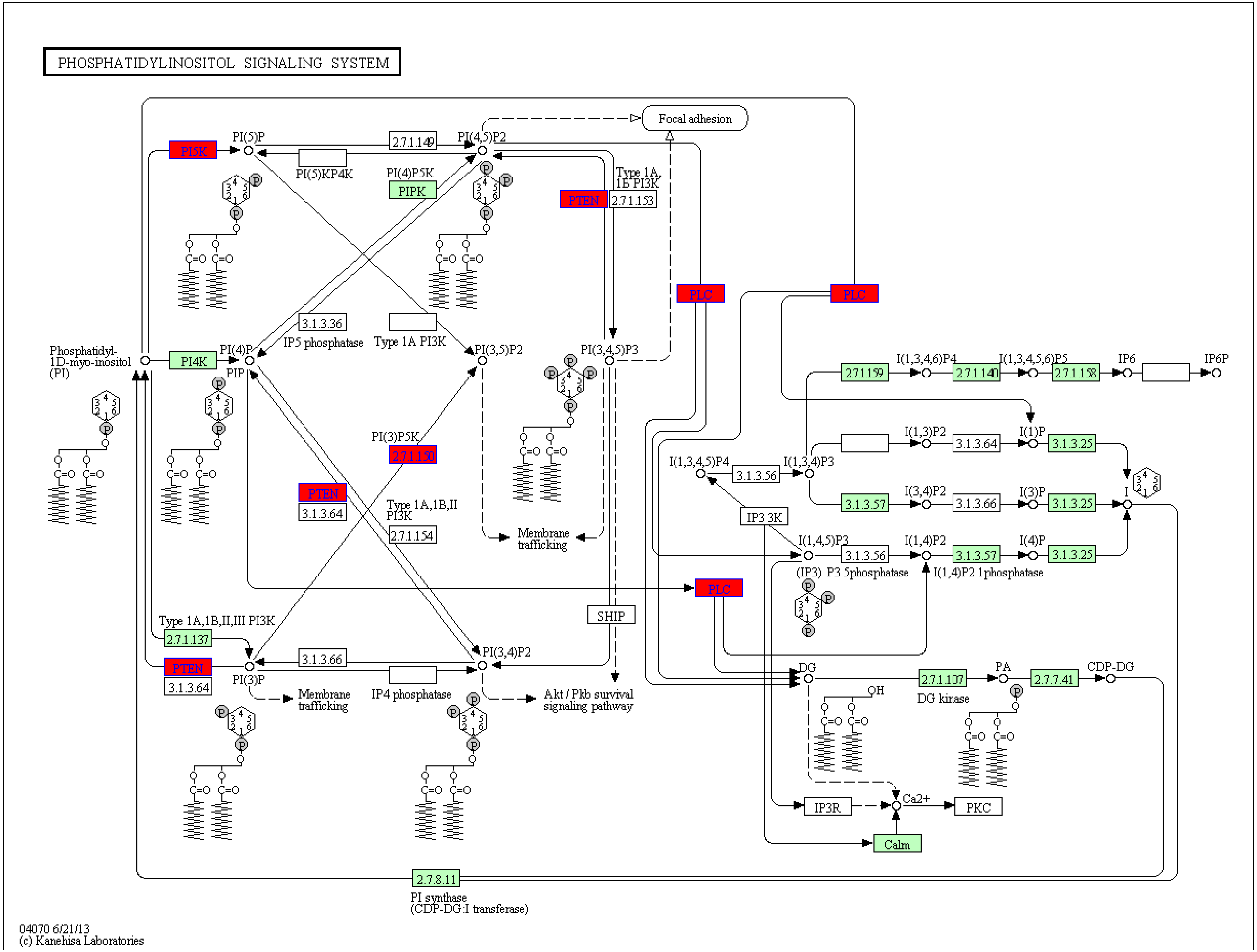

PHOSPHATIDYLINOSITOL SIGNALING SYSTEM
Focal adhesion
PI5K
PI(5)P
2.7.1.149
PI(4,5)P2
PI(5)P4K
PI(4)P5K
PIPK
Type 1A, 1B PI3K
PTEN
2.7.1.153
PLC
PLC
3.1.3.36
IP5 phosphatase
Type 1A PI3K
Phosphatidyl-1D-myo-inositol (PI)
PI4K
PI(4)P
PIP
PI(3,5)P2
PI(3,4,5)P3
2.7.1.159
I(1,3,4,6)P4
2.7.1.140
I(1,3,4,5,6)P5
2.7.1.158
IP6
IP6P
PI(3)P5K
2.7.1.150
I(1,3)P2
3.1.3.64
I(1)P
3.1.3.25
I(1,3,4,5)P4
3.1.3.56
I(1,3,4)P3
PTEN
3.1.3.64
Type 1A,1B,II PI3K
2.7.1.154
3.1.3.57
I(3,4)P2
3.1.3.66
I(3)P
3.1.3.25
I
IP3 3K
Membrane trafficking
I(1,4,5)P3
3.1.3.56
I(1,4)P2
3.1.3.57
I(4)P
3.1.3.25
(IP3) P3 5phosphatase
I(1,4)P2 1phosphatase
PLC
SHIP
Type 1A,1B,III PI3K
2.7.1.137
3.1.3.66
PI(3,4)P2
DG
2.7.1.107
PA
2.7.7.41
CDP-DG
PTEN
3.1.3.64
PI(3)P
OH
DG kinase
Membrane trafficking
IP4 phosphatase
Akt / Pkb survival signaling pathway
Ca2+
IP3R
PKC
Calm
2.7.8.11
PI synthase (CDP-DG:I transferase)
04070 6/21/13
(c) Kanehisa Laboratories

AD

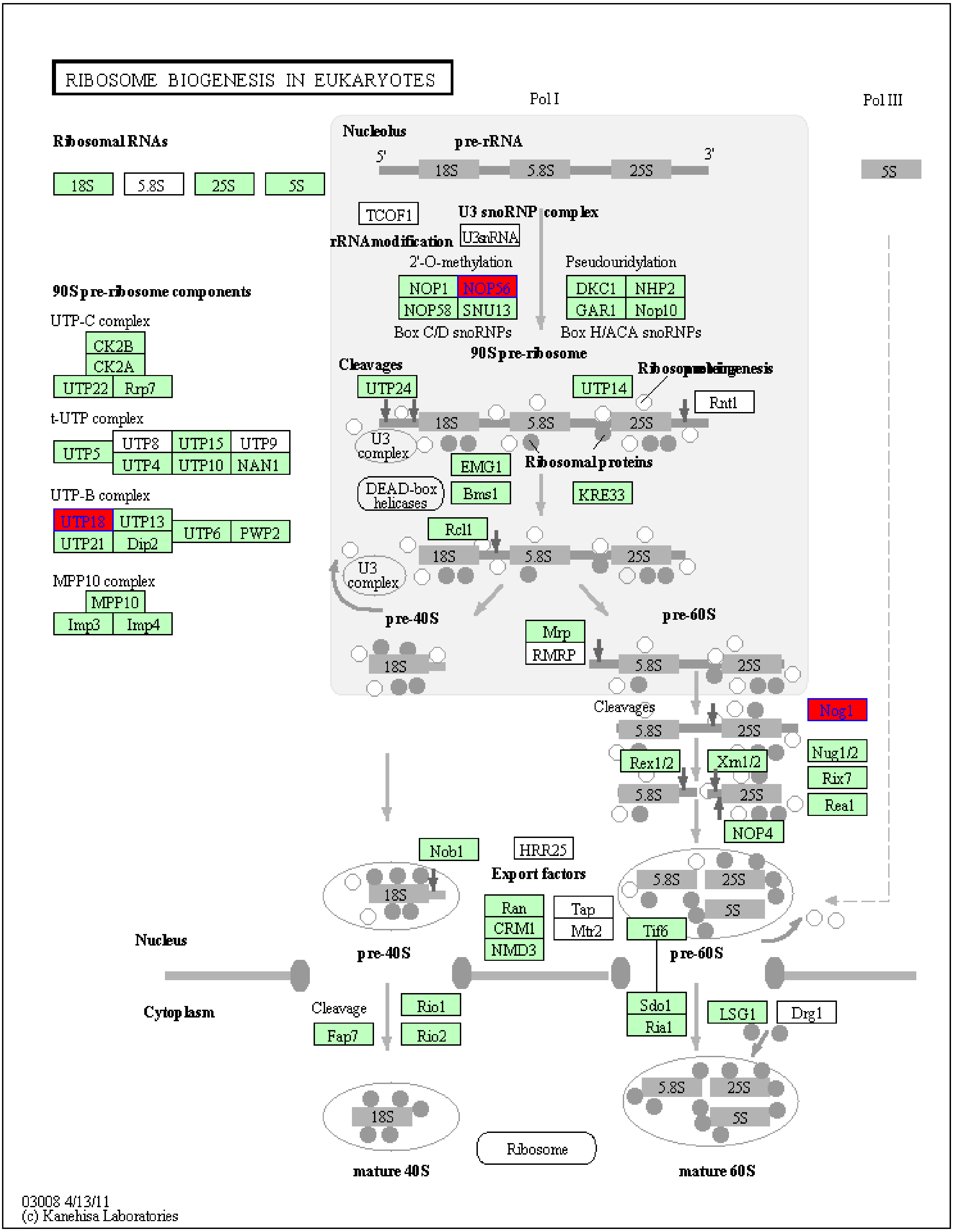
RIBOSOME BIOGENESIS IN EUKARYOTES
Pol I
Pol III
Ribosomal RNAs
18S
5.8S
25S
5S
Nucleolus
pre-rRNA
5'
3'
TCOF1
U3 snoRNP complex
U3snRNA
rRNA modification
2'-O-methylation
Pseudouridylation
NOP1
NOP56
NOP58
SNU13
DKC1
NHP2
GAR1
Nop10
Box C/D snoRNPs
Box H/ACA snoRNPs
90S pre-ribosome components
UTP-C complex
CK2B
CK2A
UTP22
Rrp7
t-UTP complex
UTP5
UTP8
UTP15
UTP9
UTP4
UTP10
NAN1
UTP-B complex
UTP18
UTP13
UTP21
Dip2
UTP6
PWP2
MPP10 complex
MPP10
Imp3
Imp4
90S pre-ribosome
Cleavages
UTP24
UTP14
Rnt1
U3 complex
EMG1
Ribosomal proteins
DEAD-box helicases
Bms1
KRE33
Rcl1
pre-40S
pre-60S
Mrp
RMRP
Nog1
Nug1/2
Rix7
Rea1
Rex1/2
Xrn1/2
NOP4
Nob1
HRR25
Export factors
Ran
CRM1
NMD3
Tap
Mtr2
Tif6
Nucleus
Cytoplasm
Cleavage
Rio1
Fap7
Rio2
Sdo1
Ria1
LSG1
Drg1
mature 40S
Ribosome
mature 60S
03008 4/13/11
(c) Kanehisa Laboratories

AE

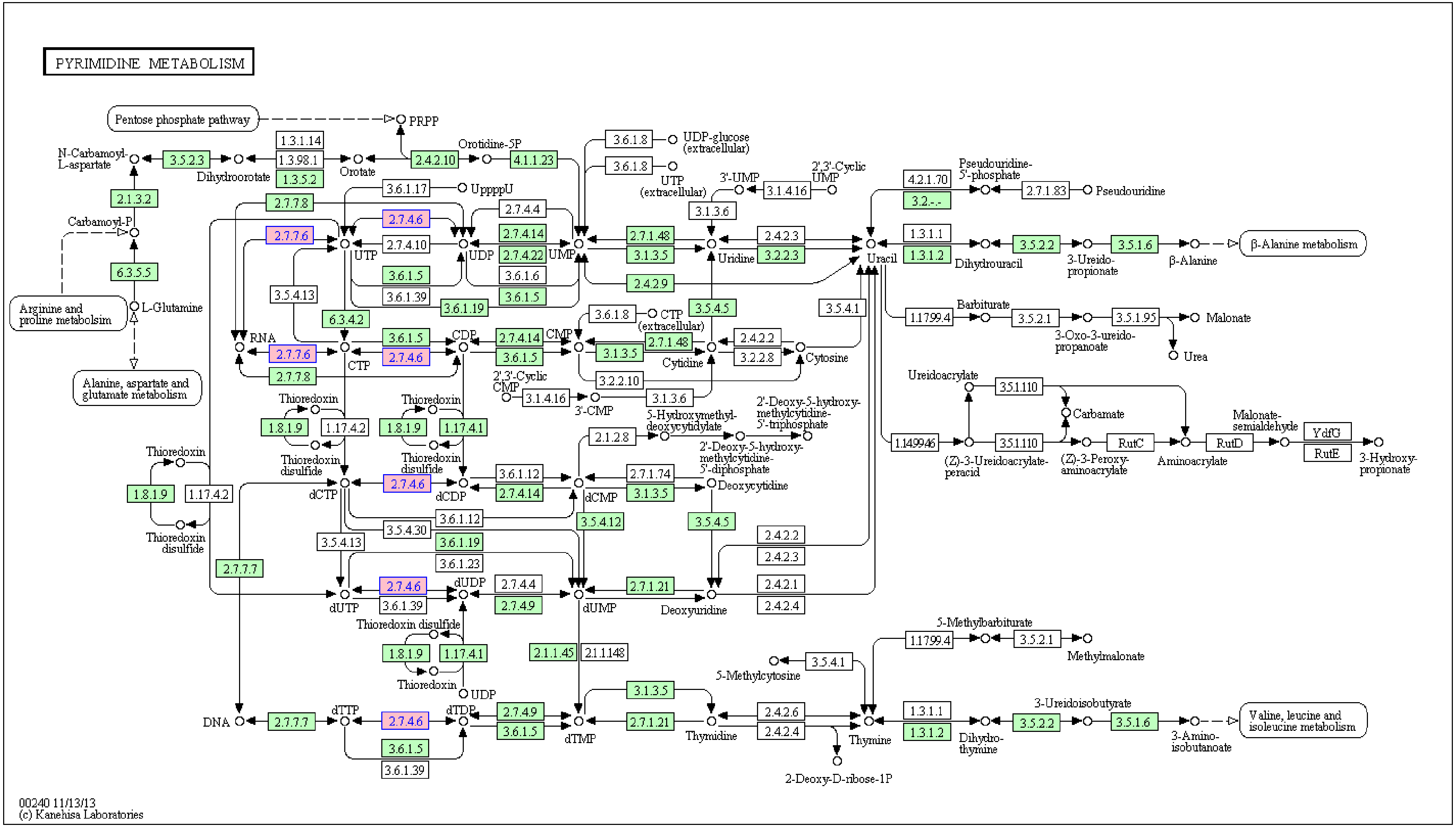
PYRIMIDINE METABOLISM
Pentose phosphate pathway
PRPP
1.3.1.14
1.3.98.1
1.3.5.2
Orotidine-5P
2.4.2.10
4.1.1.23
N-Carbamoyl-L-aspartate
3.5.2.3
Dihydroorotate
Orotate
2.1.3.2
Carbamoyl-P
6.3.5.5
L-Glutamine
Arginine and proline metabolsim
Alanine, aspartate and glutamate metabolism
2.7.7.8
3.6.1.17
UppppU
2.7.4.6
2.7.7.6
UTP
2.7.4.10
UDP
2.7.4.4
2.7.4.14
2.7.4.22
UMP
3.6.1.5
3.6.1.6
3.6.1.39
3.6.1.19
3.5.4.13
6.3.4.2
RNA
CTP
CDP
CMP
3.6.1.8
UDP-glucose (extracellular)
UTP (extracellular)
3'-UMP
3.1.4.16
2',3'-Cyclic UMP
3.1.3.6
2.7.1.48
3.1.3.5
Uridine
2.4.2.3
3.2.2.3
2.4.2.9
3.5.4.5
CTP (extracellular)
2.4.2.2
3.2.2.8
Cytidine
Cytosine
3.2.2.10
2',3'-Cyclic CMP
3'-CMP
5-Hydroxymethyl-deoxycytidylate
2'-Deoxy-5-hydroxy-methylcytidine-5'-triphosphate
2'-Deoxy-5-hydroxy-methylcytidine-5'-diphosphate
2.1.2.8
Thioredoxin
Thioredoxin disulfide
1.8.1.9
1.17.4.2
1.17.4.1
dCTP
dCDP
3.6.1.12
2.7.1.74
dCMP
Deoxycytidine
3.5.4.12
3.5.4.30
3.6.1.23
dUTP
dUDP
2.7.4.9
dUMP
2.7.1.21
Deoxyuridine
2.4.2.1
2.4.2.4
2.7.7.7
2.1.1.45
2.1.1.148
DNA
dTTP
dTDP
dTMP
Thymidine
2.4.2.6
Thymine
2-Deoxy-D-ribose-1P
5-Methylcytosine
3.5.4.1
Uracil
Pseudouridine-5'-phosphate
4.2.1.70
3.2.-.-
2.7.1.83
Pseudouridine
1.3.1.1
1.3.1.2
Dihydrouracil
3.5.2.2
3-Ureido-propionate
3.5.1.6
β-Alanine
β-Alanine metabolism
Barbiturate
1.17.99.4
3.5.2.1
3-Oxo-3-ureido-propanoate
3.5.1.95
Malonate
Urea
Ureidoacrylate
3.5.1.110
Carbamate
1.14.99.46
(Z)-3-Ureidoacrylate-peracid
(Z)-3-Peroxy-aminoacrylate
RutC
Aminoacrylate
RutD
Malonate-semialdehyde
YdfG
RutE
3-Hydroxy-propionate
5-Methylbarbiturate
Methylmalonate
3-Ureidoisobutyrate
Dihydro-thymine
3-Amino-isobutanoate
Valine, leucine and isoleucine metabolism
00240 11/13/13
(c) Kanehisa Laboratories
